# Identification and characterization of known and novel microRNAs in strawberry fruits induced by *Botrytis cinerea*

**DOI:** 10.1038/s41598-018-29289-7

**Published:** 2018-07-19

**Authors:** Yaoxin Liang, Yuhan Guan, Shaoxi Wang, Yanjun Li, Zhihong Zhang, He Li

**Affiliations:** Liaoning Key Laboratory of Strawberry Breeding and Cultivation, College of Horticulture, Shenyang Agricultural University, 120 Dongling Road, Shenyang, 110866 China

## Abstract

MicroRNAs are endogenous small non-coding RNAs that negatively regulate mRNAs, mainly at the post-transcriptional level, and play an important role in resistance response of plants. To date, there are few reports on resistance response of strawberry miRNAs to pathogens. In this study, using high-throughput sequencing, 134 conserved and 35 novel miRNAs were identified in six libraries within the treatment of *Botrytis cinerea*. A total 497 potential target genes were predicted using *Fragaria vesca* genome. Most of the differential expressed miRNAs in strawberry fruits were up-regulated in early libraries and down-regulated in late libraries. *PIRL*, the target gene of miR5290a, showed the opposite expressed trend compared with miR5290 from T1 to T3 libraries, and functional analysis of the *PIRL* gene shows that it has obvious resistance to *B*. *cinerea* in the strawberry fruits with overexpressed *PIRL* gene. We speculate that miR5290a negatively regulates its target gene *PIRL* to increase resistance to pathogen infection, and further analysis of *PIRL* function is meaningful for studying the plant-pathogen relationship and improving strawberry fruit quality and yield.

## Introduction

MicroRNAs (miRNAs) are endogenous small, 21–24 nt, non-coding RNAs that negatively regulate mRNAs, mainly at the post-transcriptional level, by degradation or translational repression^1^. Plant miRNAs are produced from a long primary transcript called pri-miRNA and then processed by a series of nuclease enzymes. Pri-miRNA length ranges from hundreds to thousands of bases, with a 5′-methylated cap, a 3′-poly (A) tail and 1 to several hairpin loop structures^2,3^. The hairpin structure within pri-miR is recognized by DCL1 proteins to generate precursor miRNA (pre-miR), which is approximately 70 bases in length^4^. Pre-miRNA is a sequence of a single hairpin structure, which is further cut into a single stranded mature miRNA with a length of approximately 21 bases^5^. Then, miRNA binds to argonaute (AGO) proteins to form an RNA-induced silencing complex (RISC), and then the RISC binds to target mRNAs to silence them.

MiRNAs can cleave mRNA by binding to complementary regions of mRNAs. In plants, miRNAs mainly either perfectly or near-perfectly cleave their targets by binding to them and therefore repress the expression of target genes. However, when a miRNA imperfectly binds to a mRNA, it inhibits the translation of target genes^6^. Therefore, the types and expression levels of target mRNAs could detected to analyze the function of miRNAs. In previous studies, plant miRNAs have been discovered to be involved in almost all biological and metabolic processes^7,8^, especially as key players in plant-pathogen interactions^9^. Recent studies have shown that during the invasion of *Botrytis cinerea*, miRNAs are involved in the defense response in herbaceous peony^10^. The transcription factor *NAC4* has been found to promote pathogen-induced cell death in *Arabidopsis* under negative regulation by miRNA164^11^. The *Arabidopsis* miR396 has been discovered to mediate pathogen-associated molecular pattern-triggered immune responses against fungal pathogens^12^. In addition, miRNAs have been shown to mediate the regulation of gene expression in the response of rice plants to fungal elicitors^13^. Thus, to understand comprehensively about plant miRNAs, it is critical to identify miRNAs and further analyze the relationship between miRNA and target genes in response to fungal pathogens.

Initially, cloning is used to discover miRNAs^14^. Then, more miRNAs from a wider range of plant species were identified^15^. Currently, high-throughput sequencing has become the most widely used method to furtherly explore miRNAs and their biological function^16^. High-throughput sequencing can sequence hundreds of thousands to millions of DNA molecules at one time, but the average read length is short. So far, many new discoveries have been made by high-throughput sequencing, e.g., in cotton^17^, tomato^18^, and rice^19,20^. In strawberry, there have been some reports of miRNAs using high-throughput sequencing, but mainly, studies have focused on the identification and expression of miRNAs in different strawberry varieties or different strawberry organs^5,21–25^. For example, strawberry miRNAs have been found to be involved in the abiotic stress response^26^. The miRNA-mediated mechanism of senescence of strawberry fruit was studied by analyzing the content of plant hormones and the expression of miRNAs and their target genes^25^. However, to date, few studies have been completed on the role of pathogen-induced miRNAs in the defense response of strawberry.

The cultivated strawberry (*Fragaria* × *ananassa*) is a perennial herb, and it has a strong ornamental and unique taste, with high economic value^5,27,28^. It consumed for both its unique flavor and its nutrient content throughout the world^27^. Global annual production has reached four million tons in the last few years (http://faostat.fao.org). At present, protected cultivation is the main cultivation form of strawberry production, and diseases spread easily in cultivated strawberries in protected fields. Strawberries in protected areas have been discovered to be susceptible to gray mold, powdery mildew, rotten fruit disease and so on. These diseases will seriously affect the growth, development, yield and quality of strawberry. Among them, strawberry gray mold is an important disease in strawberry production caused by *B*. *cinerea*. *B*. *cinerea*, a typical necrotrophic fungal pathogen^29^, causes a class of fungal diseases of plant flowers, leaves and fruits. It infects almost all vegetable and fruit crops^30^, such as *Solanum lycopersicum*^31^, *Prunus avium*^32^, *Vitis vinifera*^33^, *Lactuca sativa*^34^. So far, there have been many reports about the disease resistance of strawberry^35–45^. Most of them studied the resistance of different strawberry varieties to a variety of plant diseases or control methods to strawberry diseases. Nevertheless, only a few studies on the resistance response of small RNA to pathogens in plants have been reported^30,46^, especially less in strawberries^47^. In this study, six small RNA libraries were constructed from red ripening fruits taken at 48 h (T1), 72 h (T2), 96 h (T3) and 120 h (T4) after *B*. *cinerea* treatment, and two controls (fruits treated with sterile water, CK1; *B*. *cinerea*, CK2). The differential expression of miRNAs and their target genes in strawberry fruit after pathogen treatment were studied by high-throughput sequencing technology to reveal the miRNA response to pathogen stress and the miRNA regulation mechanism.

## Results

### High-throughput sequencing of small RNA

Six small RNA libraries from infected fruits (T1/T2/T3/T4) of the strawberry cultivar ‘Yanli’ and two controls (CK1 and CK2) were generated. High-throughput sequencing of small RNA libraries yielded 18 to 27 million raw reads *per* library, which had 20,547,608 (T1), 19,447,590 (T2), 26,043,435 (T3), 24,024,124 (T4), 21,199,911 (CK1) and 18,984,247 (CK2) high quality reads for six libraries (Table 1). After discarding some contaminant reads containing sequences shorter than 18 nt, adaptor and poly A sequences, etc., 12,459,104 (T1:60.64%), 12,019,783 (T2:65.16%), 19,394,365 (T3:74.47%), 18,742,399 (T4:78.01%), 12,927,907 (CK1:60.98%) and 15,110,298 (CK2:79.59%) reliable clean reads remained for analysis.

**Table 1:** Summary of total sRNAs sequencing from six libraries.

Category	CK1		T1		T2		T3		T4		CK2	
	Number	Percent (%)	Number	Percent (%)	Number	Percent (%)	Number	Percent (%)	Number	Percent (%)	Number	Percent (%)
total_reads	21750527		21282022		19122236		26754613		24583971		19450616	
high_quality	21199911	100	20547608	100	18447590	100	26043435	100	24024124	100	18984247	100
3′ adapter_null	480033	2.26	187212	0.91	168122	0.91	345399	1.33	212689	0.89	437851	2.31
insert_null	23640	0.11	6392	0.03	12199	0.07	20561	0.08	23047	0.10	38485	0.20
5′ adapter_contaminants	17532	0.08	15641	0.08	18995	0.10	51034	0.20	22246	0.09	44281	0.23
smaller_than_18nt	671781	3.17	863116	4.20	1711660	9.28	2173575	8.35	1269318	5.28	1061825	5.59
polyA	1733	0.01	995	0.00	825	0.00	1710	0.01	1321	0.01	468	0.00
expressed less than 5	7077285	33.38	7015148	34.14	4516006	24.48	4056791	15.58	3753104	15.62	2291039	12.07
clean_reads	12927907	60.98	12459104	60.64	12019783	65.16	19394365	74.47	18742399	78.01	15110298	79.59

Note: T1, T2, T3, and T4 indicated that after inoculation, red ripening fruits were chose and took at 48 h, 72 h, 96 h and 120 h, respectively. CK1 indicated that red ripening fruits were took from strawberry plants treated with sterile water. CK2 indicates *B*. *cinerea*. The same applies to the following figures and tables.

**Figure 1:**
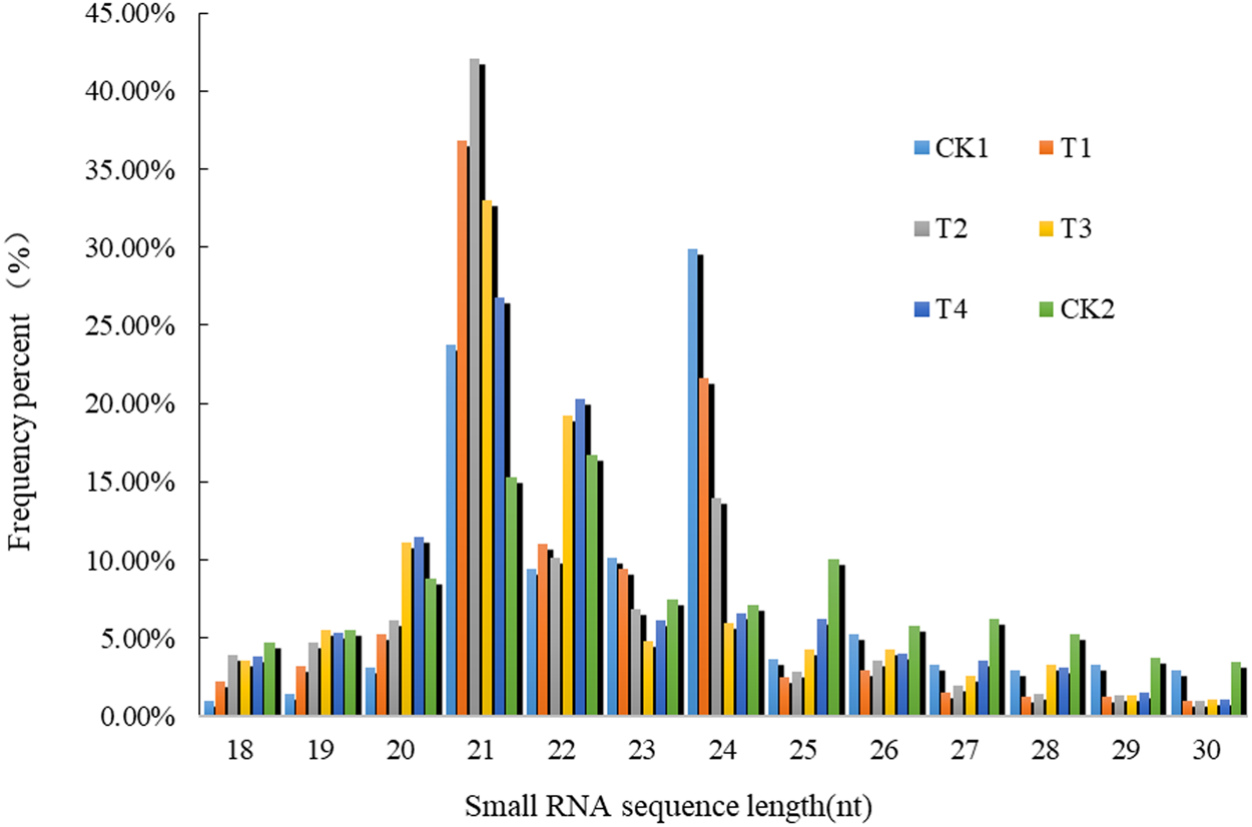
Size distribution of small RNAs in six libraries. T1, T2, T3, and T4 indicated that after inoculation, red ripening fruits were took at 48 h, 72 h, 96 h and 120 h, respectively. CK1 indicated that red ripening fruits were took from strawberry plants treated with sterile water. CK2 indicates *B*. *cinerea*. The same applies to the following figures and tables.

The size frequency distribution of the unique small RNAs was uneven (Fig. 1). The overall distribution pattern was similar in six libraries. The length of small RNA varied from 18 to 30 nt, but small RNAs were 21–24 nt in length, with 21 nt as the major size classes for five libraries, while 24 nt was the most abundant class in the CK1 library (Fig. 1). In the six libraries, the most abundant class was 21 nt, which accounted for 23.77%, 36.83%, 42.06%, 33.04%, 26.81%, 15.26% in CK1, T1, T2, T3, T4, CK2 libraries, respectively. This result is consistent with previous studies^22,48,49^. The second most abundant class in the CK1 (29.87%) and T1 (21.59%) libraries was 24 nt, and this is the same as that in the study of Li *et al*.^5^, who found that the 21 nt and 24 nt sRNAs were the most and the second most abundant classes, respectively, in wild type and white-flesh mutant strawberry libraries. Interestingly, the 22 nt length of small RNAs also had a large proportion in the T3 (19.23%), T4 (20.27%) and CK2 (16.75%) libraries, respectively.

Furtherly, the unique sRNAs were classified into different RNA categories (Table 2). The data showed that 38.03% (T1), 36.97% (T2), 12.99% (T3), 8.88% (T4), 37.90% (CK1) and 1.44% (CK2) of the unique sRNAs matched the genome of woodland strawberry (*Fragaria vesca*). Further analysis of six libraries with sRNA sequences searched against the Rfam database and GenBank revealed that 12% to 24% of the sequences matched non-coding RNAs, including rRNAs, snRNAs, snoRNAs and tRNA. Less than 1.00% of the unique sequences matched known miRNAs in the CK1, T3 and T4 libraries, respectively. Only 1.67% and 2.41% of the T1 and T2 libraries matched known miRNAs by searching against miRDeep2. There were no sequences detected to match known miRNAs in the CK2 library.

**Table 2:** Distribution of sRNAs among different categories from six libraries.

Category	Total RNAs						Unique sRNAs					
	CK1	T1	T2	T3	T4	CK2	CK1	T1	T2	T3	T4	CK2
Total	12927907 (100.00%)	12459104 (100.00%)	12019783 (100.00%)	19394365 (100.00%)	18742399 (100.00%)	15110298 (100.00%)	261989 (100.00%)	241618 (100.00%)	176773 (100.00%)	283491 (100.00%)	271741 (100.00%)	234452 (100.00%)
Mapping to genome	6901797 (53.39%)	7334509 (58.87%)	7417912 (61.71%)	2972744 (15.33%)	1752644 (9.35%)	153637 (1.02%)	99291 (37.90%)	91890 (38.03%)	65360 (36.97%)	36837 (12.99%)	24127 (8.88%)	3377 (1.44%)
rRNA	2655686 (20.54%)	1694394 (13.60%)	2070793 (17.23%)	3872395 (19.97%)	5138616 (27.42%)	5814161 (38.48%)	35638 (13.60%)	24230 (10.03%)	27726 (15.68%)	34652 (12.22%)	28664 (10.55%)	48093 (20.51%)
snRNA	74502 (0.58%)	42547 (0.34%)	39037 (0.32%)	67099 (0.35%)	94537 (0.50%)	74156 (0.49%)	1497 (0.57%)	1250 (0.52%)	1139 (0.64%)	1435 (0.51%)	1404 (0.52%)	1476 (0.63%)
snoRNA	60660 (0.47%)	46930 (0.38%)	50385 (0.42%)	51735 (0.27%)	51114 (0.27%)	77904 (0.52%)	1507 (0.58%)	1293 (0.54%)	1367 (0.77%)	835 (0.29%)	600 (0.22%)	885 (0.38%)
tRNA	1303429 (10.08%)	593504 (4.76%)	524645 (4.36%)	1004784 (5.18%)	903795 (4.82%)	543269 (3.60%)	7098 (2.71%)	6518 (2.70%)	4753 (2.69%)	5118 (1.81%)	3569 (1.31%)	5470 (2.33%)
Matching known miRNAs	1101757 (8.52%)	1543596 (12.39%)	3194405 (26.58%)	456281 (2.35%)	163388 (0.87%)	0(0.00%)	2523 (0.96%)	4040 (1.67%)	4266 (2.41%)	1320 (0.47%)	632 (0.23%)	0(0.00%)
Unannotated sRNAs	7731873 (59.81%)	8538133 (68.53%)	6140518 (51.09%)	13942071 (71.89%)	12390949 (66.11%)	8600808 (56.92%)	213726 (81.58%)	204287 (84.55%)	137522 (77.80%)	240131 (84.70%)	236872 (87.17%)	178528 (76.15%)

### Conserved miRNAs in strawberry fruits treated by *B*. *cinerea*

To identify conserved miRNAs in strawberry, all small RNA sequences were searched against the currently known miRNAs in miRDeep2. As a result, a total 134 conserved miRNAs were identified in six libraries. These identified miRNAs cover 51 miRNA families generated by high-throughput sequencing (Tables 3 and S1).

**Table 3:** Summary information on conserved miRNAs found in six libraries.

miRNA family	Members	Normalized MiR read						Ratio	Sequence of a miRNA who has the highest count in the whole family	length (nt)
		CK1	T1	T2	T3	T4	CK2	T4/CK1		
miR156	7	5720.73	17890.14	9590.57	10471.83	2236.52	0	0.39	UUGACAGAAGAGAGUGAGCAC	21
miR157	1	431.03	1146.59	1086.19	925.39	218.77	0	0.51	UUGACAGAAGAUAGUGAGCAC	21
miR159	17	11768.65	39493	53347.57	5715.15	1591.91	0.69	0.14	UUUGGAUUGAAGGGAGCUCUA	21
miR162	1	187.89	196.16	202.58	7.57	3.04	0	0.02	UCGAUAAACCUCUGCAUCCAG	21
miR164	1	182.13	1197.41	1405.64	97.59	32.17	0	0.18	UGGAGAAGCAGGGCACGUGCA	21
**miR166**	**3**	**1567**.**61**	**4487**.**97**	**5471**.**58**	**1736**.**89**	**315**.**3**	**0**.**59**	**0**.**2**	**UCGGACCAGGCUUCAUUCCCC**	**21**
**miR167**	**1**	**1128**.**77**	**2491**.**2**	**3045**.**56**	**198**.**59**	**25**.**88**	**0**	**0**.**02**	**UGAAGCUGCCAGCAUGAUCUG**	**21**
miR319	2	977.88	3066.39	2165.65	742.77	161.38	0	0.17	UUGGACUGAAGGGAGCUCCUC	21
miR395	1	155.19	67.37	131.39	15.72	9.28	0	0.06	CUGAAGUGUUUGGGGGAACUC	21
miR396	3	1801.89	2768.82	2987.61	426.62	169.27	0	0.09	UUCCACAGCUUUCUUGAACUU	21
miR398	1	1296.14	3922.91	1053.05	0	79.2	0	0.06	GGUGCGACCUGAGAUCACAUG	21
miR399	1	42.62	70.85	120.88	3.58	2.27	0	0.05	UGCCAAAGGAGAGUUGCCCUG	21
miR408	1	296.69	717.67	759.28	104.58	63.01	0	0.21	ACAGGGACGAGGUAGAGCAUG	21
miR477	1	53.8	130.03	93.91	21.05	14.39	0	0.27	CUCUCCCUCAAGGGCUUCUC	20
miR482	1	138.85	401.23	562	23.21	3.15	0	0.02	UCUUUCCAAUUCCUCCCAUGCC	22
miR1026	1	0	42.31	101.21	0	0	0	Both 0	UUGUGAAAUGACUUGAGAGGUG	22
miR1511	32	65121.9	138635.14	142694.28	31493.51	7107.36	3.79	0.11	AACCGGCUCUGAUACCAAUUG	21
miR1861	1	0	0	0	0	379.49	0	≫1	UCGAUCUUGUGAGCAGACUGU	21
miR3437	1	369.28	516.09	308.37	146.84	10	0	0.03	CCUGGACUUGUAUUUUUGUAC	21
miR3522	1	0	0	0	416.65	0	0	Both 0	UAGACCAAUUGACAGCUCUGU	21
miR3633	1	80.41	156.02	164.38	23.96	0	0	≫1	UCCCUAUUCCACCUAUUCCCCA	22
miR3708	1	0	0	0	126.04	0	0	Both 0	UCACAAAGAUGUCGUCGUAUA	21
miR4380	1	0	142.79	0	0	0	0	Both 0	CGAUUGUUGAUCCGAAUGUUGAUC	24
miR4398	1	0	0	0	122.13	0	0	Both 0	UCUCAGCGGAGGAGAAAGGAC	21
miR4414	1	0	0	0	0	146.18	0	≫1	UCGCAAGGAUGCGGAGCGUGA	21
miR5213	1	441.55	882.59	677.5	273.22	32.33	0	0.07	UGCGAGUGUCUUCACCUCUGAA	22
miR5258	1	0	0	0	173.21	0	0	Both 0	UCAAGCUGACAAAGAAGACUG	21
miR5260	1	0	0	0	828.3	0	65.36	Both 0	UUUGACUGUUGCUCAUGGCCU	21
miR5289	2	296.23	352.18	440.48	311.57	0	0	≫1	CGGAAAACUGAAAUUCGGCGG	20
**miR5290**	**3**	**1474**.**3**	**4791**.**19**	**6292**.**27**	**665**.**32**	**241**.**15**	**0**	**0**.**16**	**UUGGAGAGAGAGUAGACAAUG**	**21**
miR5642	1	0	0	0	103.41	0	0	Both 0	UCUCGCAGCUUGUAGGUGCU	20
miR5643	1	0	0	0	237.53	0	0	Both 0	UGGCUCUUUAAGAUCGGCUGG	21
miR5656	1	0	0	0	501.42	0	0	Both 0	UCGAAGUGGAGAUUGUGUUUU	21
miR5663	1	0	0	0	103.41	0	0	Both 0	UGAGAAAAUGCAACUCUUAGCG	22
miR5813	1	29.45	75.5	156.75	34.69	6.45	0	0.22	AAGACAGCAGGACGGUGGUCAUG	23
miR6103	1	0	0	0	129.29	0	0	Both 0	UUGUUCAGACAACCCUGGGAAC	22
miR6437	1	0	0	0	219.39	0	0	Both 0	UCACGGACGGUAGGCUUGAAGC	22
miR7708	1	46.72	104.35	26.33	32.36	17.99	0	0.39	UGUAAUGACUGCACAAGACUGC	22
miR7732	1	0	0	0	0	142.62	0	≫1	UCGAGAUCUUGGAGGGGACCC	21
miR7767	1	54.66	209.01	422.34	33.11	28.77	0	0.53	CCAAGAUGAGUGCUCUCCU	19
miR7782	23	32787.52	82247.59	103253.18	15917.77	2591.57	1.33	0.08	ACCUAGCUCUGAUACCAUGUG	21
miR7984	1	440.89	1007.59	1125.6	403.58	137.57	0	0.31	UCCGACUUUGUGAAAUGACUU	21
miR8122	1	0	0	0	0	151.44	0	≫1	UUCCGACAAGACUUUUCUCUU	21
miR8175	1	9.46	55.46	103.06	152.91	41.66	0	4.4	UUCCCGGCAAUGGAACCA	18
miR8183	1	0	0	0	314.07	10.72	27.96	≫1	UUUCAGUUCGAAGGAUUGUG	20
miR8581	1	148.91	116.26	0	0	0	0	≫1	UGGAAAUUCUUGUAUGCACGACGU	24
miR8666	1	0	0	0	338.19	0	0	Both 0	UUUGGUGAUAUAGACGUAAAU	21
miR8781	1	114.76	0	0	0	0	0	≫1	UUUGAUUGUGAAGUUUGACGGAGA	24
miR9472	1	0	0	0	0	230.99	0	≫1	UUCCAAUCUCUGAUACAAUG	20
miR9653	1	0	131.73	206.76	0	0	0	Both 0	ACCAAGAUCUCUGAGGUCU	19
miR9767	1	0	0	247.85	0	0	0	Both 0	UGAAAAGGACUUUGAAAAAAG	21
Total:	134	127165.91	307513.54	338243.82	73592.42	16201.83	99.72			

Among these miRNA families, miR1511 (32 members), miR7782 (23 members) and miR159 (17 members) were larger families, with 17 or more members than others. In addition, miR156 (7 members), miR166 (3 members), miR396 (3 members), miR5290 (3 members), miR319 (2 members) and miR5289 (2 members) also had multiple family members. Except for these 9 larger miRNA families, the other 42 miRNA families only had one member, such as miR157, miR164, miR167 and so on. The expression levels of these larger miRNA families, such as miR156, miR159, miR1511 and miR7782, were extraordinarily high in the T1, T2, T3, T4, and CK1 libraries (Table 3). For example, reads of the miR1511 family accounted for 45.08%, 42.19%, 42.80%, 43.87% and 51.21% of the reads in the T1, T2, T3, T4, and CK1 libraries, respectively. Meanwhile, the expression levels of some miRNAs with fewer family members were also very high, such as miR166, miR167 and miR5290.

Through next generation high-throughput sequencing, the differential expressions were also detected among different members in a family, which facilitates us to further understand the role of miRNAs in plant growth and development. In miRNA families with multiple members, miRNA names containing “a” tended to expressed far more than other members do, such as miR166a, whose expression level was much higher than that of other family members. Similarly, the expression levels of miR156a, miR159a, miR396a and miR5290a in their families were significantly higher than that of other family members.

The conservation of these 134 miRNAs was also detected in other plants, such as *A*. *thaliana*, *O*. *sativa*, *V*. *vinifera*, *C*. *sinensi*, and *M*. *domestic*. Among these miRNAs, 41 miRNAs belonging to 14 miRNA families showed that they were conservative in a variety of plant species. Among them, the sequences of miR156a, miR164, and miR166a in strawberry were identical to those in other species. In addition, miR159a, miR167, miR319, miR395, miR396, and miR399 in strawberry were conserved in a variety of plant species. However, the conservation of some miRNA families was very low in other plants, especially miR1511, which had 32 family members and each of its family members had poor conservation in other plants. In this study, we also tried to identify the precursor sequences for the 134 conserved strawberry miRNAs. Approximately 23 pre-miRNAs and their secondary structures of miRNAs were identified according to the genome of *F*. *vesca*. A single copy in the *F*. *vesca* genome encoded nineteen of these miRNA sequences while the other 4 sequences had 2, 3, 4 and 5 loci in the genome, respectively (Table S1).

### Novel miRNAs in strawberry fruits treated by *B*. *cinerea*

**Figure 2:**
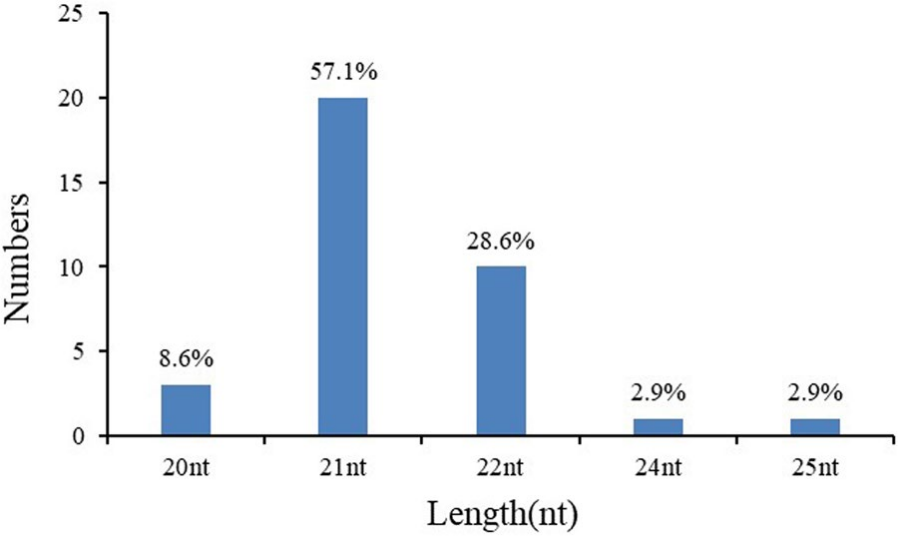
Different size distributions of novel miRNAs in six libraries.

Not only were the conserved miRNAs identified, but some new miRNA sequences were also found in six libraries from the remaining sRNA sequences after excluding the rRNAs, tRNAs, snRNAs, snoRNAs and known miRNAs. Based on the criteria for plant miRNAs^50^, 35 unique sRNA sequences were identified in six libraries (Table 4). The class of 21 nt sequences was the most dominant in novel miRNAs, and the second most abundant class was 22 nt sequences (Fig. 2). A total of 35 sRNA sequences were coded by 55 loci, and most of them were only generated from one locus, but there were 7 miRNAs produced from several loci, including fan-novel-007, 009, 010, 014, 016, 022, and 023. Among these 7 miRNAs, fan-novel-007 came from 15 loci, while the others were generated from 2 loci. The length of the potential novel miRNA precursors varied from 80 to 342 nt. Low free energy was also detected as an important characteristic of novel miRNAs. The minimum free energy (MFE) value of these miRNA precursors was from −289.01 to −40.2 kcal/mol. Additionally, the structures of the 35 novel miRNA precursors are shown Fig. S1.

**Table 4:** Novel miRNAs identified in six libraries.

Novel miRNA Name	Len (nt)	Sequence (5′-3′)	Pre len	MFE	miRNA location in the *F*.*vesca* genome	Actual MiR read						Normalized MiR read						Ratio	Actual MiR* reads					
			(nt)	(Kcal/mol)		CK1	T1	T2	T3	T4	CK2	CK1	T1	T2	T3	T4	CK2	(T4/CK1)	CK1	T1	T2	T3	T4	CK2
fan-novel-001	21	AAGUUUGGCUUCGAAUCUGGA	80	−40.2	LG5:2486945:2487024:-	852	1818	1074	0	49	0	56.39	140.63	86.2	0	2.53	0	0.04	0	0	0	0	0	0
fan-novel-002	20	AGGGCGAAUACACUCUCAGA	103	−50.02	LG0:1732172:1732274:+	0	0	0	1660	0	0	0	0	0	138.11	0	0	Both 0	0	0	0	32	0	0
fan-novel-003	20	AGUGGUAUCAGGGCUAUGUU	101	−41.9	LG5:1343331:1343431:-	2241	6322	2390	1413	1932	0	148.31	489.02	191.83	117.56	99.62	0	0.67	0	0	0	0	45	0
fan-novel-004	21	AUUGGAUUGAAGGGAGCUCUC	194	−96.33	LG7:2338693:2338886:-	4528	15811	19330	2384	0	0	299.66	1223.01	1551.48	198.34	0	0	0	6	12	0	6	0	0
fan-novel-005	21	CAGGGCGAAUACACUCUCAGA	103	−50.02	LG0:1732172:1732274:+	739	2562	2331	0	0	0	48.91	198.18	187.09	0	0	0	0	13	5	36	0	0	0
fan-novel-006	21	CAUGGGAAGUUUGGAAAGAAU	87	−30.4	LG4:464031:464117:-	1290	5028	1590	438	0	0	85.37	388.93	127.62	36.44	0	0	0	0	0	0	0	0	0
fan-novel-007	21	CGGAAAACUGGAAUUUGGCGG	101	−63.4	LG2:24236074:24236174:+	2675	0	0	0	450	0	177.03	0	0	0	23.2	0	0.13	14	0	0	0	0	0
			101	−53.75	LG0:1372856:1372956:-	2626	1941	1743	1449	450	0	173.79	150.14	139.9	120.55	23.2	0	0.13	0	0	0	0	0	0
			101	−68.8	LG6:29806366:29806466:+	2675	0	1751	0	450	0	177.03	0	140.54	0	23.2	0	0.13	0	0	0	0	0	0
			101	−69.5	LG6:25384574:25384674:+	2670	0	1751	0	450	0	176.7	0	140.54	0	23.2	0	0.13	0	0	0	0	0	0
			122	−80.6	LG5:14442383:14442504:+	2670	0	0	0	450	0	176.7	0	0	0	23.2	0	0.13	0	0	0	0	0	0
			121	−80.33	LG5:17078464:17078584:-	2670	0	1751	0	450	0	176.7	0	140.54	0	23.2	0	0.13	0	0	0	0	0	0
			101	−58.96	LG1:9465096:9465196:-	2668	1958	1751	1454	450	0	176.57	151.46	140.54	120.97	23.2	0	0.13	0	0	0	0	0	0
			100	−72.4	LG4:9975328:9975427:-	2434	0	1674	0	444	0	161.08	0	134.36	0	22.89	0	0.14	0	0	0	0	0	0
			100	−68.5	LG3:16718652:16718751:+	2626	1941	1743	1449	450	0	173.79	150.14	139.9	120.55	23.2	0	0.13	0	0	0	0	0	0
			101	−76.5	LG2:13285529:13285629:+	2668	1958	1751	1454	450	0	176.57	151.46	140.54	120.97	23.2	0	0.13	0	0	0	0	0	0
			100	−58.3	LG6:21746845:21746944:+	2427	1831	1674	0	0	0	160.62	141.63	134.36	0	0	0	0	0	0	0	0	0	0
			270	−100.3	LG6:21746904:21747173:+	0	0	0	1397	444	0	0	0	0	116.23	22.89	0	≫1	0	0	0	0	0	0
			342	−289.01	LG6:10959096:10959437:-	0	0	1751	0	450	0	0	0	140.54	0	23.2	0	≫1	0	0	0	0	0	0
			101	−68.8	LG6:30942704:30942804:+	2675	0	1751	0	450	0	177.03	0	140.54	0	23.2	0	0.13	0	0	0	0	0	0
			100	−67.2	LG1:18104293:18104392:+	2670	0	1751	0	450	0	176.7	0	140.54	0	23.2	0	0.13	0	0	0	0	0	0
fan-novel-008	25	CGGUAUAGUCUUUCCUGAGCUCUUG	90	−46	LG4:6036334:6036423:+	0	0	0	0	2577	0	0	0	0	0	132.87	0	≫1	0	0	0	0	12	0
fan-novel-009	21	CGUGUUCUCAGGUCGCCCCUG	123	−52.9	LG3:21882009:21882131:-	15830	22991	9051	0	0	0	1047.63	1778.4	726.46	0	0	0	0	2737	8503	2278	0	0	0
			170	−80.2	LG3:21882084:21882253:-	15847	22999	9056	0	0	0	1048.75	1779.02	726.86	0	0	0	0	2737	8503	2278	0	0	0
fan-novel-010	22	CUUUGAUGACUGUGUGAUGAUG	109	−26.8	LG5:26821965:26822073:-	742	1303	709	193	0	0	49.11	100.79	56.91	16.06	0	0	0	0	0	0	0	0	0
			109	−27.3	LG2:8056778:8056886:-	742	1303	709	193	0	0	49.11	100.79	56.91	16.06	0	0	0	0	0	0	0	0	0
fan-novel-011	21	GGAGCGACCUGAGACCACAUG	184	−82.22	LG3:21882081:21882264:-	0	0	0	2646	0	0	0	0	0	220.14	0	0	Both 0	0	0	0	0	0	0
fan-novel-012	22	GGAGUGACCUUGAGAACACAGG	100	−39.3	LG2:21103074:21103173:-	0	17534	0	0	1064	0	0	1356.29	0	0	54.86	0	≫1	0	0	0	0	0	0
fan-novel-013	24	GUCGAGGAUCUUGACGAGUUCAUC	171	−51.6	LG3:4529757:4529927:+	0	0	1651	0	0	0	0	0	132.51	0	0	0	Both 0	0	0	125	0	0	0
fan-novel-014	22	UCAUCCAACACAUCAUCGGCAU	175	−63.53	LG5:12409802:12409976:-	0	0	1774	796	243	0	0	0	142.39	66.22	12.53	0	≫1	0	0	33	7	9	0
			190	−67.53	LG5:12409792:12409981:-	655	2029	0	0	0	0	43.35	156.95	0	0	0	0	0	32	58	0	0	0	0
fan-novel-015	22	UCCCUAUUCCACCUAUUCCCCA	120	−51.4	LG4:24750968:24751087:-	1039	1601	1672	197	53	0	68.76	123.84	134.2	16.39	2.73	0	0.04	35	108	47	112	43	0
fan-novel-016	21	UCCGUUGUAGUCUAGUUGGUC	190	−53.19	LG1:8108613:8108802:+	0	5397	0	0	0	0	0	417.47	0	0	0	0	Both 0	0	0	0	0	0	0
			217	−40.12	LG5:12849297:12849513:-	0	5405	0	0	0	0	0	418.09	0	0	0	0	Both 0	0	0	0	0	0	0
fan-novel-017	21	UCGAGGAUCUUGACGAGUUCA	188	−52	LG3:4529750:4529937:+	0	0	1651	0	0	0	0	0	132.51	0	0	0	Both 0	0	0	125	0	0	0
fan-novel-018	22	UCUAUUCAAAGAGAUGACUGUU	108	−58.3	LG4:2714635:2714742:+	4343	8023	3561	4108	0	0	287.42	620.6	285.82	341.77	0	0	0	0	0	0	0	0	0
fan-novel-019	22	UCUUGCCGAUACCUCCCAUUCC	119	−54.7	LG5:13604440:13604558:-	621	1338	1244	83	0	0	41.1	103.5	99.85	6.91	0	0	0	25	68	10	17	0	0
fan-novel-020	22	UCUUUCCUAGUCCUGCCAUUCC	125	−57.5	LG6:2405315:2405439:+	1002	2734	2209	210	0	0	66.31	211.48	177.3	17.47	0	0	0	745	1433	862	180	0	0
fan-novel-021	22	UCUUUCCUAUUCCUCCCAUCCC	105	−55.2	LG5:13604730:13604834:-	0	0	1991	0	0	0	0	0	159.8	0	0	0	Both 0	0	0	611	0	0	0
fan-novel-022	21	UGAAGUGGGAUUUGGCGAAUU	114	−46.8	LG3:25359222:25359335:+	9593	14427	14441	0	0	0	634.87	1115.96	1159.07	0	0	0	0	61	78	31	0	0	0
			92	−41.6	LG3:25359232:25359323:+	0	0	0	2405	0	0	0	0	0	200.09	0	0	Both 0	0	0	0	29	0	0
fan-novel-023	21	UGAAUUGGGAUUUGGCGAAUU	320	−81.72	LG3:25355964:25356283:+	0	2121	0	0	111	0	0	164.06	0	0	5.72	0	≫1	0	0	0	0	0	0
			105	−48.7	LG3:25355900:25356004:+	828	0	1438	1153	0	0	54.8	0	115.42	95.93	0	0	0	0	0	0	0	0	0
fan-novel-024	20	UGACAGAAGAGAGUGAGCUC	127	−56.5	LG1:192269:192395:+	558	1305	538	133	19	0	36.93	100.94	43.18	11.07	0.98	0	0.03	9	7	5	0	0	0
fan-novel-025	21	UGGAGCCUGCGAGGGGGAAUG	144	−62.9	LG5:2676771:2676914:+	2217	4770	1701	417	621	0	146.72	368.97	136.53	34.69	32.02	0	0.22	0	0	0	0	0	0
fan-novel-026	21	UGGGAUUGGGCGAAUUUUGGU	122	−40.2	LG3:25352640:25352761:+	870	1488	1018	358	0	0	57.58	115.1	81.71	29.78	0	0	0	27	60	70	17	0	0
fan-novel-027	21	UGGGAUUUGGCGAAUUGUGGU	108	−46.3	LG3:25352218:25352325:+	974	1930	1860	479	325	0	64.46	149.29	149.29	39.85	16.76	0	0.26	0	7	8	0	0	0
fan-novel-028	21	UUACCCUUGCAAUAUCCGUUG	124	−60.3	LG1:17600630:17600753:-	1986	5049	5280	0	0	0	131.43	390.55	423.79	0	0	0	0	77	217	121	0	0	0
fan-novel-029	21	UUCGUGAUCUGCGAAAGGCUC	119	−54.2	LG2:20038976:20039094:+	6386	14059	6073	3358	844	0	422.63	1087.49	487.43	279.37	43.52	0	0.1	157	614	342	385	268	0
fan-novel-030	22	UUGCGGUCUUGUCUCUUCCAAU	131	−57	LG7:15528979:15529109:-	1266	1730	2511	504	399	0	83.78	133.82	201.54	41.93	20.57	0	0.25	7	29	11	0	0	0
fan-novel-031	21	UUGGACUGAAGGGAGCUCCCC	197	−86.87	LG5:2300704:2300900:-	4432	14788	10222	2212	0	0	293.31	1143.88	820.44	184.03	0	0	0	0	0	0	0	0	0
fan-novel-032	21	UUGGACUGAAGGGAGCUCCCU	194	−80	LG3:28637412:28637605:+	4728	13414	9869	0	954	0	312.9	1037.6	792.11	0	49.19	0	0.16	0	0	0	0	0	0
fan-novel-033	21	UUGGACUGAAGGGAGCUCCUC	205	−81.51	LG7:18365232:18365436:+	0	0	0	3598	753	0	0	0	0	299.34	38.83	0	≫1	0	0	0	0	0	0
fan-novel-034	21	UUGGAGAGAGAGUAGACAAUG	111	−69.1	LG6:12165619:12165729:+	24476	0	0	8200	4790	0	1619.82	0	0	682.21	246.98	0	0.15	3456	0	0	295	268	0
fan-novel-035	22	UUGUCUACUCUCUCUCGAAAGG	93	−61.2	LG6:12165627:12165719:+	0	4284	5978	0	0	0	0	331.38	479.81	0	0	0	Both 0	0	159	259	0	0	0

The actual read span of novel miRNAs was large in the six libraries, varying from 0 to 22,999. Among these novel miRNAs, the reads of fan-novel-004, 009, 012, 022, 029, 031 and 032 were more than 10,000 in some of the six libraries. However, the actual reads of all novel miRNA* strands were clearly less than those for their corresponding mature miRNAs because of the rapid degradation in the process of mature miRNA formation, and their counts were mostly 0. Besides, 15 novel miRNA* strands were not detected which need further confirmation.

### Cluster analysis of differentially expressed miRNAs in strawberry fruits induced by *B*. *cinerea*

**Figure 3:**
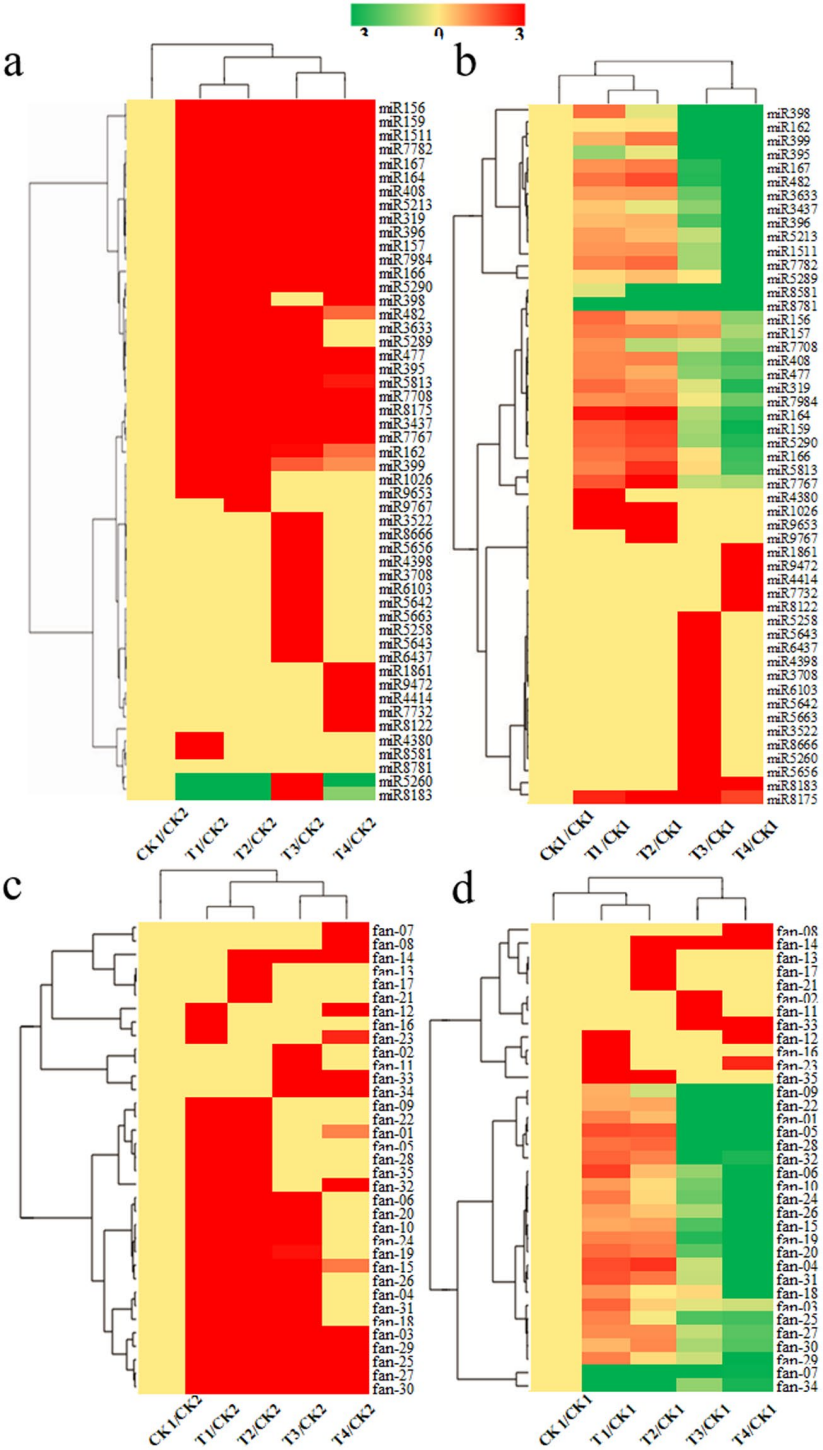
Cluster analysis chart of miRNAs. (**a**) Differential expression of the 51 most abundant conserved miRNAs between different infected libraries and CK2, (**b**) differential expression of the 51 most abundant conserved miRNAs between different infected libraries and CK1, (**c**) differential expression of 35 novel miRNAs between different infected libraries and CK2, (**d**) differential expression of 35 novel miRNAs between different infected libraries and CK1. The miRNA expression levels are shown as Z-scores. The column represents different samples, and rows represent different miRNAs; the results are clustered by log10 (TPM+1) value. Red means high miRNA expression and green means low miRNA expression.

Based on qvalue < 0.005 and |log2 (foldchange)| ≥ 1 criteria, differential expressions of 51 most abundant conserved miRNAs and 35 novel miRNAs in six libraries were analyzed (Fig. 3). To determine the differential expression of miRNAs caused by *B*. *cinerea* treatment, the expression levels in the T1, T2, T3, and T4 libraries were compared to those in the CK1 and CK2 libraries. We found that most of the 51 conserved miRNAs from the T1 to T4 libraries were up-regulated compared to CK2. However, two conserved miRNAs (miR5260 and miR8183) were down-regulated in the T1, T2 and T4 libraries compared to CK2 (Fig. 3a). In addition, all of novel miRNAs were up-regulated or showed no change compared to CK2 (Fig. 3c).

Compared with the expression in the CK1 library, approximately 28 conserved miRNAs were down-regulated in the T3 and T4 libraries, and most of them were up-regulated in the T1 and T2 libraries, except miR395, miR8581 and miR8781 (Fig. 3b). For example, the T1/CK1 and T2/CK1 ratios of miR5290a were 3.25 and 4.27, respectively; however, the T3/CK1 and T4/CK1 ratios were 0.45 and 0.16, respectively (Table S2). In addition to these 28 conserved miRNAs, the rest of the miRNAs were up-regulated only at a certain stage or in two libraries. Moreover, miR8175 was up-regulated in the T1, T2, T3, and T4 libraries (Fig. 3b). The novel miRNAs were similar to conserved miRNAs in that approximately 23 novel miRNAs were up-regulated in the T1 and T2 libraries and down-regulated in the T3 and T4 libraries compared to the CK1 stage. The other 12 novel miRNAs were up-regulated or showed no changes (Fig. 3d and Table S3). These results suggest that their expression levels were inhibited with prolonged infection time, and these miRNAs may play a major role in the late infected libraries.

**Figure 4:**
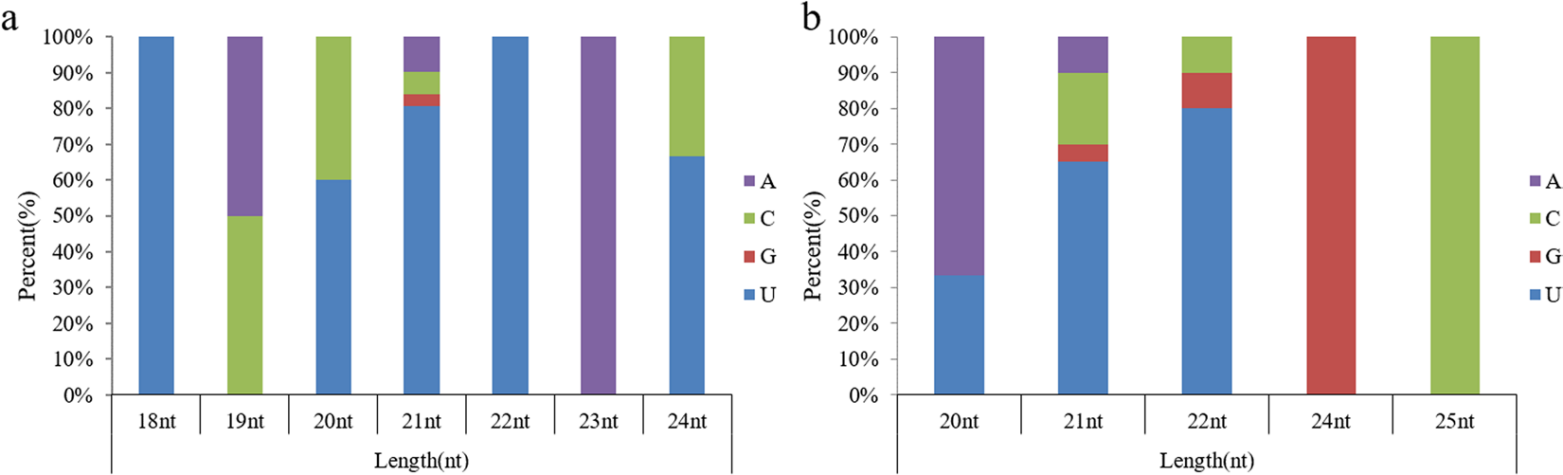
The first nucleotide at the 5′ terminal of the miRNA sequences. (**a**) The 51 most abundant conserved miRNAs and (**b**) 35 novel miRNAs. The abscissa represents sequences of different lengths; ordinates denote the percentage of the first miRNA bases in each length.

MiRNA has a strong base preference; therefore, the first nucleotide at the 5′ terminal of the 51 most abundant conserved miRNA and the 35 novel miRNA sequences was analyzed (Fig. 4). In the conserved miRNAs, the results showed that most had a predominance of U at the 5′ terminal (Fig. 4a). This is in line with previous reports^51^. We also found that miRNAs with a length of 21 nt had the largest base species at the 5′ terminus, and the lowest number of G bases existed in 21-nt miRNAs, which is consistent with the characteristics of miRNA base preferences (Fig. 4a). In the novel miRNAs, miRNAs with lengths of 21 and 22 nt had more base species at the 5′ terminus. As with the conserved miRNA, among the 35 novel miRNA sequences, the first base of the 5′ terminal of the 22 potential novel miRNA sequences was uracil (Fig. 4b).

### qRT-PCR validation of miRNAs

**Figure 5:**
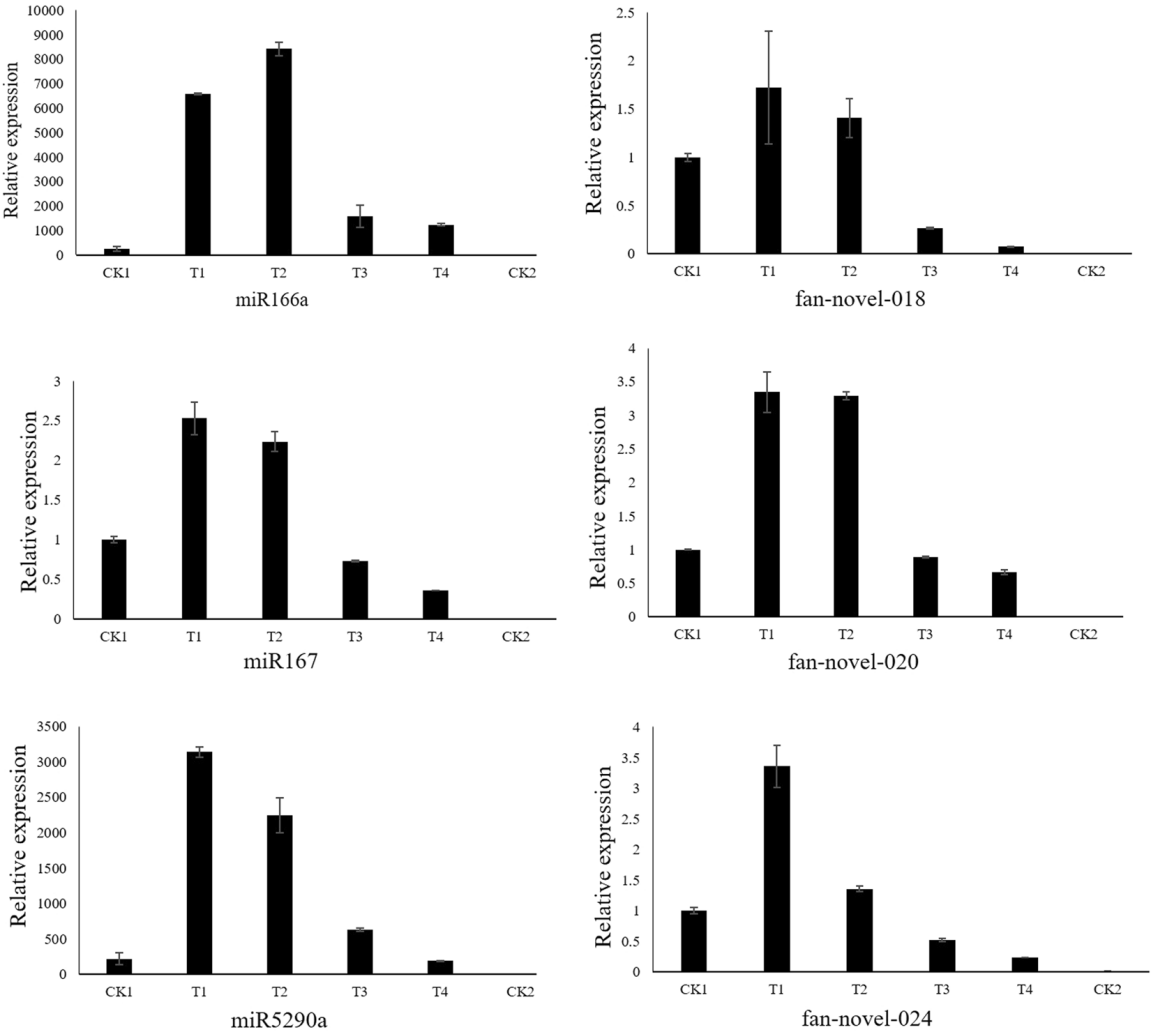
Quantitative RT-PCR validation of ‘Yanli’ miRNAs in different infected libraries (T1, T2, T3, and T4) and two controls (CK1 and CK2). The qRT-PCR reaction was repeated three times, and the template amount was corrected by strawberry 26S rRNAs. The normalized miRNA levels at CK1 were arbitrarily set to 1. The vertical bar indicates standard error.

Through internal or external standard methods, qRT-PCR can detect the expression levels of miRNAs by quantitative analysis of specific DNA sequences in the samples, and the stem-loop primers could make the reactions more sensitive and accurate to distinguish significantly the expression levels of miRNAs^52^. To confirm the results of high-throughput sequencing, we chose 6 miRNAs, including 3 conserved miRNAs (miR166a, miR167, miR5290a) and 3 novel miRNAs (fan-novel-018, fan-novel-020, fan-novel-024), with high expression levels to analyze the expression levels using stem-loop qRT-PCR. The abundance profiles of six miRNAs for qRT-PCR were similar with high-throughput sequencing (Fig. 5, Tables S2 and S3). For example, in the qRT-PCR results, the T1/CK1, T2/CK1, T3/CK1, and T4/CK1 ratios of miR167 were 2.53, 2.24, 0.73, and 0.36, respectively; this trend is line with the sequencing data. Except for miR167, most of these miRNAs were significantly up-regulated after *B*. *cinerea* infection in the early libraries and then were down-regulated after treatment for 96 h (T3). Additionally, all of these miRNAs had almost no expression in CK2. This condition illustrates that the high-throughput sequencing data of miRNAs was reliable. The qRT-PCR for miR5290a in strawberry fruits treated with sterile water was also performed due to the further study the function of its target gene *PIRL*. The qRT-PCR result showed no significant difference among CK1, T1, T2, T3, T4 libraries treated with sterile water (Fig. S2).

### Target prediction for conserved and novel miRNAs in strawberry fruits

To understand comprehensively the function of species-specific miRNAs in strawberry fruit infected with *B*. *cinerea*, it is worth predicting their target genes. In this study, a total 497 targets were predicted for the 169 miRNAs, including 134 conserved miRNAs and 35 novel miRNAs, using TargetFinder software (Tables S4 and S5).

A total 210 targets were predicted for 134 conserved miRNAs. In addition, 17 conserved miRNAs had no target in the *F*. *vesca* genome, which may caused by the different variety between octaploid strawberry ‘Yanli’ and diploid strawberry in *F*. *vesca* genome. Among the predicted targets, 62 targets were described as uncharacterized proteins. In addition, 133 and 31 target genes of miR5289 and miR5213 were predicted, respectively, which were the most and the second target gene descriptions in all of the targets. These predicted target genes were mainly derived from the *SPL*, *NAC*, *HL-Zip*, *TMV*, *LRR*, *F-box/kelch*, *MYB* and other disease resistance-related proteins, indicating that these miRNAs may be involved in the strawberry disease response (Table S4).

In addition, the potential target genes were also identified for the novel miRNAs. As shown, a total 287 target genes were predicted for 35 novel miRNAs. Similar to conserved miRNAs, 8 miRNAs still had no target in the *F*. *vesca* genome. Among the predicted targets, 38 target genes were uncharacterized proteins. The target gene descriptions of the species-specific strawberry miRNAs were mainly *F-box/kelch*, *TF*, *SPL*, *PPR*, *TMV* and disease resistance-related proteins. This pattern of disease resistance-related proteins is similar to those of conserved miRNAs, and the function of these targets mostly related to disease resistance. Based on these results, these miRNAs could be considered involved in the resistance response to *B*. *cinerea* (Table S5). Thus, study and analysis careful of these potential target genes will help us understand better about the defense response mechanism of miRNAs in strawberry.

### Gene Ontology (GO) of miRNA target genes

**Figure 6:**
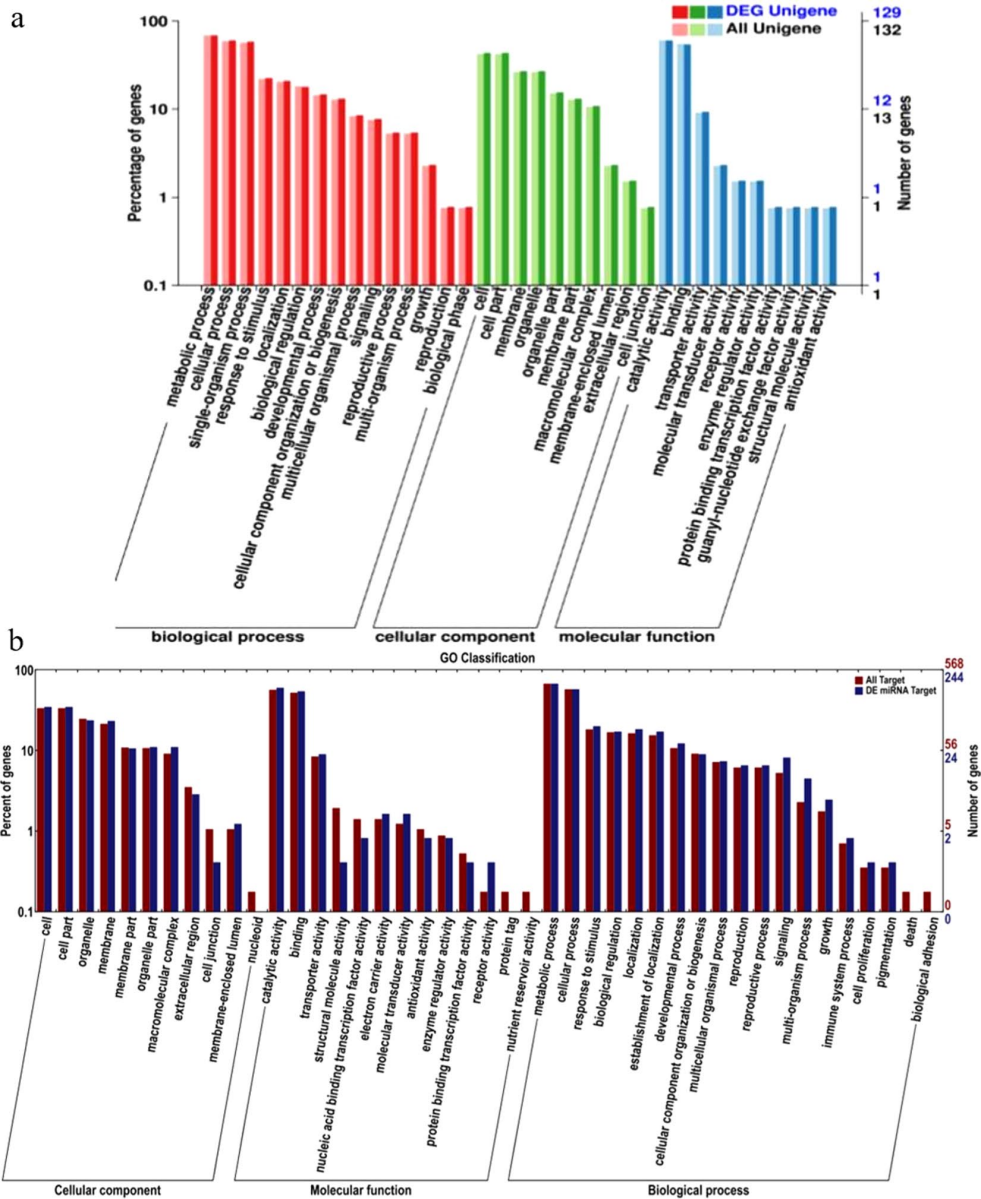
GO analysis of ‘Yanli’ strawberry miRNA targets. (**a**) The GO analysis between CK2 and T2; (**b**) The GO analysis between T2 and T4. The abscissa is classified as GO, the percentage of the number of genes on the left side of the ordinate and the number of genes on the right.

In order to furtherly analyze the biological function of miRNA targets, Gene Ontology (GO) analysis was performed on the differentially expressed genes and all targets between CK2 and T2, T2 and T4, respectively (Fig. 6). As shown in Fig. 6a, compared with T2, There is not much difference in the number of genes between the differentially expressed targets and all targets in different functions, and it may be due to that the small RNAs in *B*. *cinerea* have no direct effect to the strawberry genes.

Comparing T2 and T4, there are some specific functions with large differences in the number of target genes in the three GO terms, such as cell junction and nucleoid in cellular components (Fig. 6b). Since most of the miRNAs were up-regulated in T2 and then down-regulated in T4, the reason for the significant difference in the number of genes for these secondary functions was likely to be caused by the treatment of *B*. *cinerea*. Among them, a plant Ras-associated LRR (PIRL) protein, differentially expressed miR5290a target, there have been studies speculated that Ras proteins are involved in signal transduction^53^. Therefore, we speculated that miR5290a is likely to involve in the response mechanism of strawberry fruit against *B*. *cinerea*, and its target, *PIRL* gene may be one of the biological process signals of molecular function.

### Identification and expression analysis of *PIRL* gene

**Figure 7:**
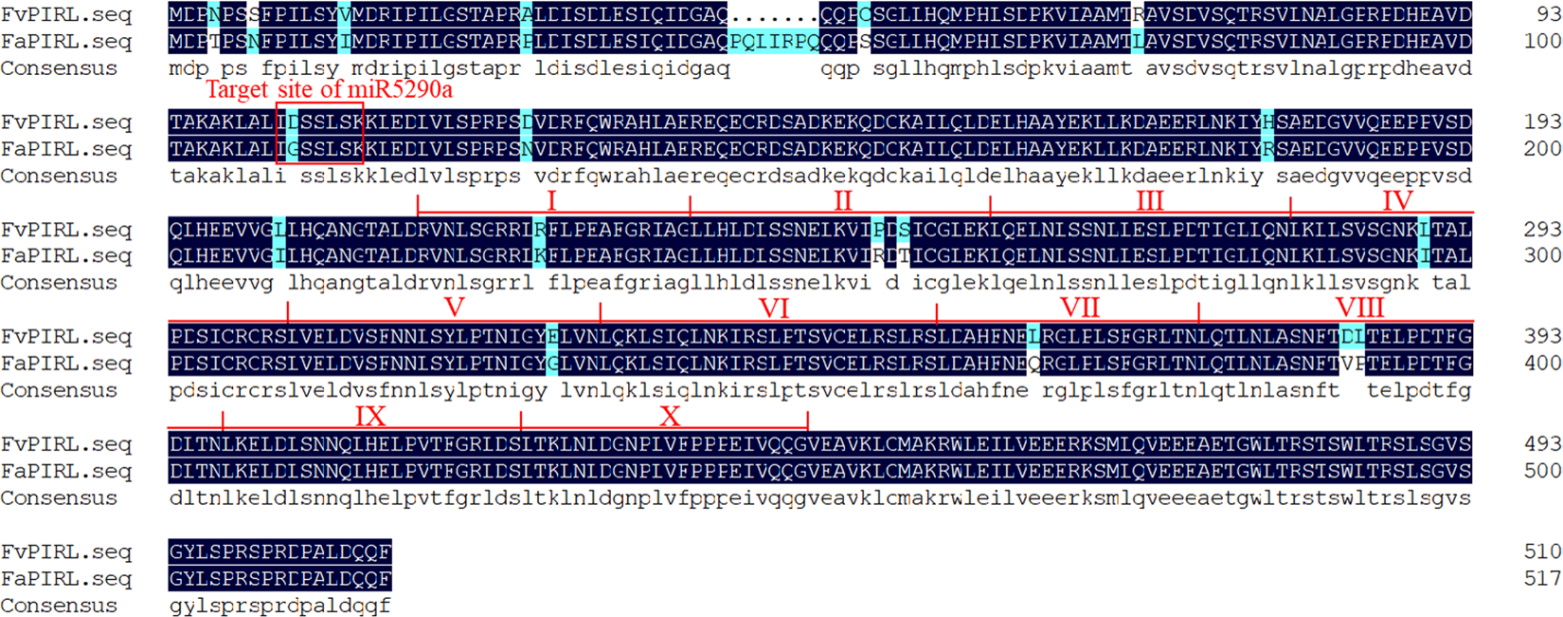
Two kinds of homologous PIRL amino acid sequence alignment and domain analysis (octoploid strawberry ‘Yanli’ and *F*. *vesca* genome). The conservative LRR domain has a red line above the sequence, and individual LRR motifs are indicated by roman numerals.

**Figure 8:**
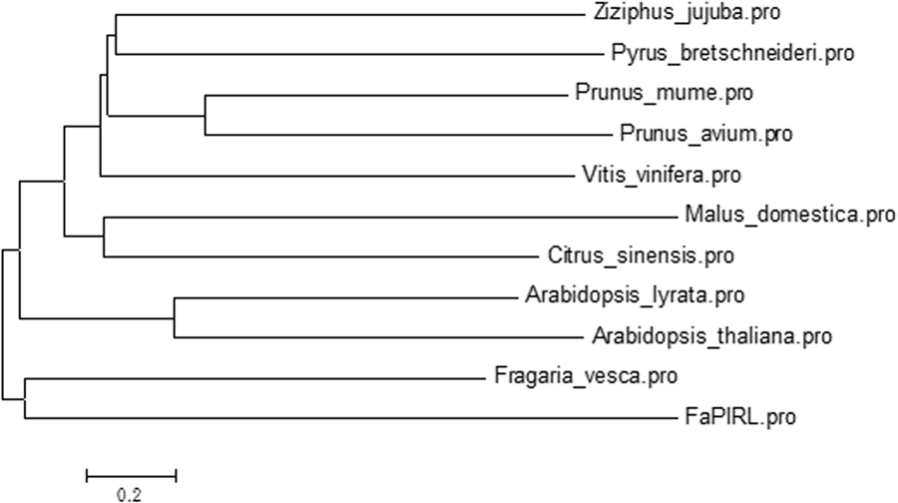
Phylogenetic tree of PIRL.

To have a clearer, deeper understanding the function of *PIRL* gene, we cloned the *PIRL* gene from the ‘Yanli’ fruits using the *PIRL* primers in Table S6. The length of *PIRL* coding region is 1554 bp and encodes 517 amino acids. To analyze the domain of PIRL protein, the amino acid sequence of octoploid strawberry ‘Yanli’ was compared with the homologous sequences of the *F*. *vesca* genome. The result showed high similarity between the two proteins, and 10 conserved LRR motifs existed in the two proteins without any other LRR class features (Fig. 7). In addition, a phylogenetic tree was constructed based on the PIRL amino acid sequences of plants to further analyze the homology of the PIRL amino acids in the ‘Yanli’ strawberry with other species. The result showed that the ‘Yanli’ strawberry PIRL (FaPIRL) was the most homologous to the diploid strawberry PIRL (FvPIRL), followed by *Arabidopsis thaliana*. This implies that this phylogenetic tree is reliable (Fig. 8).

**Figure 9:**
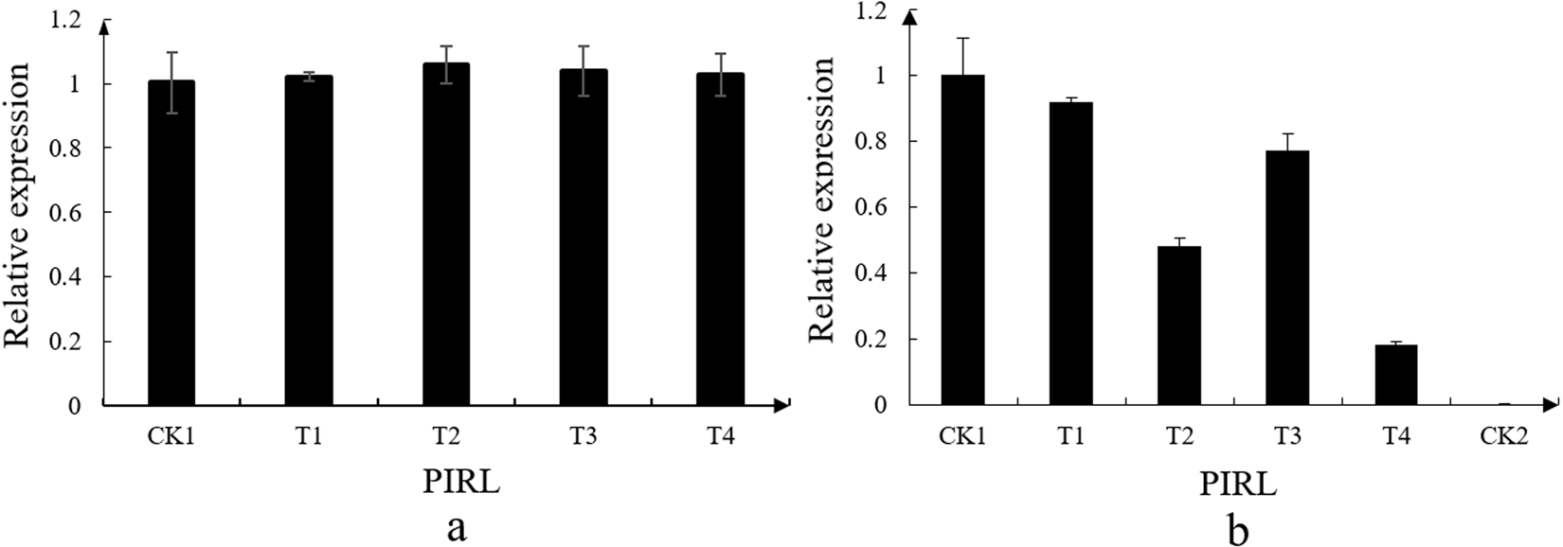
Quantitative RT-PCR validation of PIRL. (**a**) The control experiment of clean water. CK1, T1, T2, T3, and T4 indicated that after the treatment with sterile water/*B. cinerea* at 0 h, 48 h, 72 h, 96 h and 120 h, respectively. (**b**) The treatment experiment of *B. cinerea*. The qRT-PCR reaction was repeated three times, and the template amount was corrected by strawberry 26S rRNAs. The normalized miRNA levels at CK1 were arbitrarily set to 1. The vertical bar indicates standard error.

Moreover, the expressions of *PIRL* gene in the sequenced fruits were detected using qRT-PCR (Fig. 9). The qRT-PCR results showed that there was no significant difference of *PIRL* expression in strawberry fruits treated with sterile water (Fig. 9a), however, in the strawberry fruits treated with *B*. *cinerea*, the expression trend of *PIRL* was down-regulated compared to CK1, and then the expression significantly increased in T3 compared with T2 (Fig. 9b). The T1/CK1, T2/CK1, T3/CK1, and T4/CK1 ratios of *PIRL* were 0.92, 0.48, 0.77 and 0.18, respectively. However, The T1/CK1, T2/CK1, T3/CK1, and T4/CK1 ratios of miR5290a were 3.25, 4.27, 0.45 and 0.16, respectively (Table S2). This expression trend was the opposite of miR5290a from T1 to T3.

### Function analysis of ‘Yanli’ strawberry *PIRL* gene

**Figure 10:**
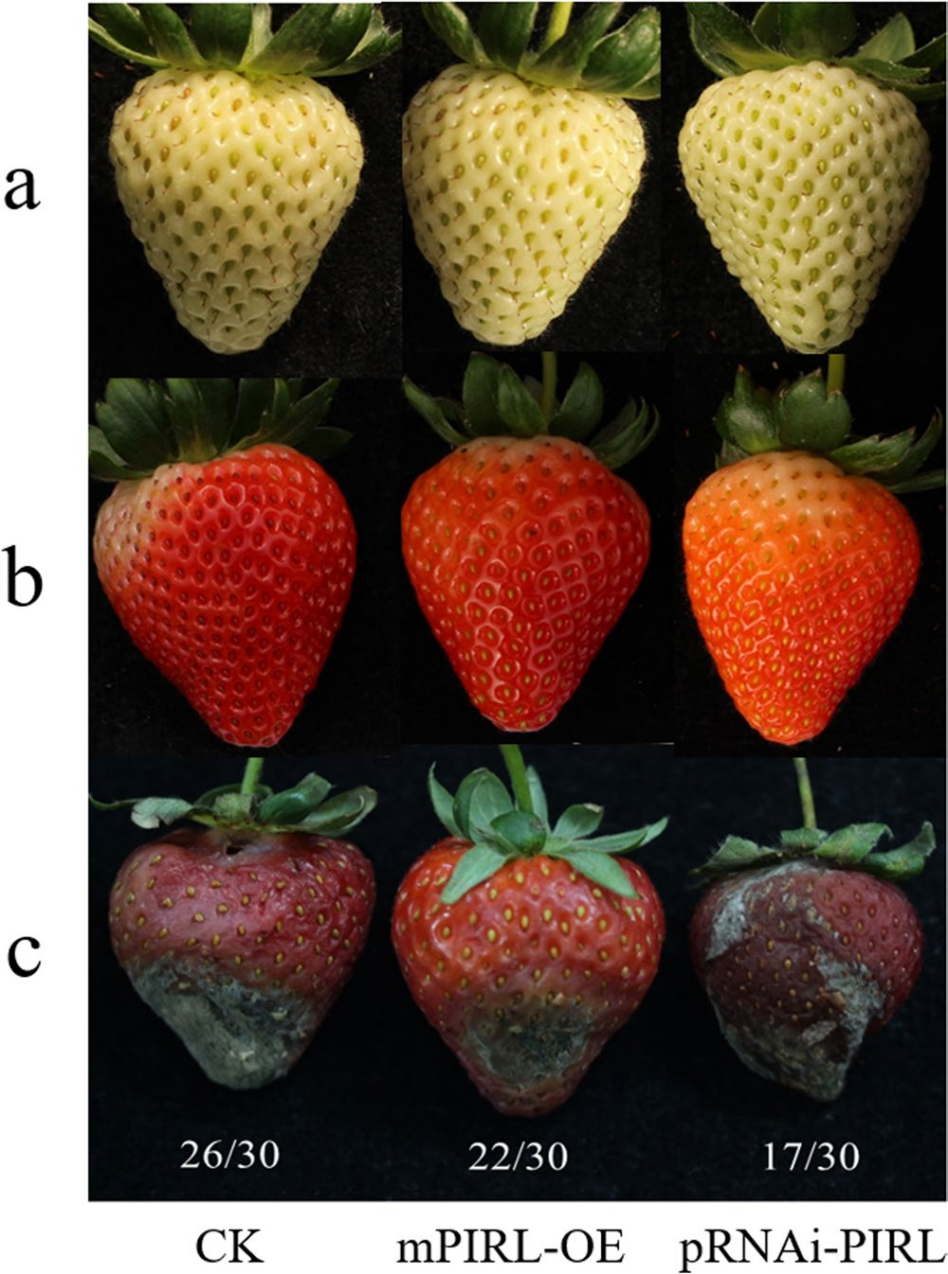
‘Yanli’ strawberry fruit phenotypes of *PIRL* gene transient expression. (**a**) The white fruits before the injection of *Agrobacterium tumefaciens* fluid; (**b**) the fruit of the turn color before the treatment of *B*. *cinerea*; (**c**) the fruit after the treatment of *B*. *cinerea*. mPIRL-OE represents fruits with overexpressed *PIRL* gene, pRNAi-PIRL represents fruits with silent *PIRL* gene. The ratios below the figure represent the fruit number of this phenotype/total fruit number.

In order to study the function of *PIRL* gene, the *PIRL* interference vector and the overexpressed *mPIRL* vector were constructed, and the transient transformation mediated by *Agrobacterium tumefaciens* was carried out using the strawberry fruits to further verify the *PIRL* function. Strawberry fruits inoculated with *B*. *cinerea* after three days of transient transformation, and the response of the fruit was observed. The fruits phenotypes showed that the fruits including interfered *PIRL* gene were more serious damaged than CK, while the fruits including overexpressed *mPIRL* (mPIRL-OE) gene were slighter (Fig. 10). Therefore, we speculated that the *PIRL* probably involved in the defense response of strawberry fruits to *B*. *cinerea*.

## Discussion

### MiRNAs and their targets in strawberry fruits induced by *B*. *cinerea*

Currently, plant miRNAs are getting more and more attention, but there are still rarely reports about the strawberry miRNAs. The analysis of miRNA target genes helped us to understand the role of miRNAs more accurately in strawberry. Based on high throughput sequencing technology, many studies have identified a number of different miRNAs from different plants^5,22,31^. In this study, there were 134 conserved miRNAs and 35 potential novel miRNAs identified under the treatment of *B*. *cinerea*. However, the numbers of identified miRNAs among the different plants are quite different, and we speculated that this may related to plant materials and the severity of the sequencing.

To deeply study the potential role of strawberry miRNAs based on the high homology of sequences between miRNAs and target genes in plants, their target genes were predicted to further analyze the function against the infection of pathogens^54^. To date, many studies have successfully predicted target genes by using this method. For example, a total 274 target genes were found in tomato for 47 miRNAs^18^. In this study, a total 497 targets for conserved and novel miRNAs were predicted in the transcripts of *F*. *vesca*. The types of target genes were mainly *TMV*, *F-box*, *NAC*, *LRR* and *MYB*. Many studies have found that these target genes are involved in disease resistance to pathogens, such as *NAC* in *Arabidopsis*^11^ and *MYB* in rice^55^. Therefore, these target genes predicted in this paper most likely to be the disease resistance related genes in the strawberry ‘Yanli’ cultivar.

### The expression of miRNA in strawberry fruit induced by *B*. *cinerea*

The identification and comparative analysis of miRNAs lay the foundation for understanding complex miRNA-mediated regulation in strawberry. In this study, most of the differential expressed 51 most abundant conserved miRNAs and 35 novel miRNAs were upregulated in the early libraries and then down-regulated in the late infected libraries compared to CK1. Except for the expression of the two comparative libraries were both 0 (Tables S2 and S3), the lowest down-regulation ratio of miRNAs was 0.02, such as the ratios of miR167 and miR482. Among the differential expressed miRNAs, miRNAs with highly expressed levels, such as miR166, miR167 and miR5290, were most representative in the conserved miRNAs, while in the novel miRNAs, fan-novel-018, fan-novel-020, and fan-novel-024 were most representative. The qRT-PCR results of these miRNAs were consistent with the sequencing results (Fig. 5, Tables S2 and S3). Moreover, the research of Zhao *et al*.^10^ proved that the expression levels of the majority of the candidate miRNAs in the herbaceous peony “Dafugui” were up-regulated^10^. Gao *et al*.^9^ discovered that most differentially expressed miRNAs were down-regulated under *B*. *elliptica* treatment in *Lilium regale* Wilson.

### The function of *PIRL* gene in strawberry fruit induced by *B*. *cinerea*

Target gene prediction could help us to understand better about the biological function of miRNAs in the defense response of fungal infection. Thus, miRNA target genes were predicted and are shown in Table S4. Many studies have shown that miRNAs participate in the regulation of plant growth and development, and negatively regulate resistance-related genes. Lee *et al*.^11^ found that under negative regulation by miRNA164, the transcription factor *NAC4* promotes pathogen-induced cell death in *Arabidopsis*^11^. Lee *et al*.^55^ found that the *MYB* gene induced by pathogens could increase disease resistance in rice^55^. Most of these target genes were resistance (R) genes. Many R genes encode pathogen proteins such as intracellular receptors, most of which are cytoplasmic nucleotide binding site (NBS) – leucine rich repeat (LRR) proteins^56^. In our results, the targets of six selected miRNAs (miR166, 167, and 5290 and fan-novel-018, 020, and 024) were *HD-Zip*, *ARF*, *PIRL*, *PPR*, *R* genes and *SPL*, respectively. In particular, the gene gi|470112365 was predicted as the target of strawberry miR5290a, which is plant intracellular Ras group-related LRR protein 9-like (*PIRL*). LRR (leucine rich repeats) domain, which is also reported to be found in disease resistant proteins by other reports^57,58^, participates involved in the interaction of proteins, ligands and proteins, which include plant immune responses and mammalian innate immune responses^59^. A motif of LRR with a structure of 20–30 amino acids (LxxLxLxxN/CxL) is unusually rich in the hydrophobic amino acid leucine. So far, there have been many reports on *LRR* gene because of the key role of the LRR domain in disease resistance, and thus the understanding of *LRR* function has been more comprehensive, especially in the *NBS-LRR* and *LRR-RLK* classes^60^. However, so far, as a class of *LRR* gene, there has rarely reports about *PIRL* gene.

PIRLs have extensive N-terminal regions and 9–11 leucine-rich unit motifs that are 23 amino acids in length, and PIRLs lack other features found in some major LRR classes in plants, such as signal or transmembrane sequences, kinase domains, or nucleotide binding sequences^61,62^. Ras-group LRR proteins, it have been categorized as a distinct class of intracellular LRR proteins that can interact with the small GTPase Ras and take part in signal transduction^54^. Forsthoefel *et al*.^62^ found that *PIRL* gene took part in developmental signaling and gene regulation^62^. To date, there were no reports about disease resistance of *PIRL* function. In this paper, we cloned and analyzed the domain of *PIRL* gene in cultivar strawberry ‘Yanli’ to study *PIRL* function in disease resistance. In addition, the *PIRL* interference vector and *mPIRL* overexpression vector were constructed to further study the transient expression of *PIRL* gene in ‘Yanli’ fruits treated with *B*. *cinerea*. The ‘Yanli’ fruits phenotype showed that the fruits were more resistant to *B*. *cinerea* when *PIRL* gene was overexpressed and more sensitive to *B*. *cinerea* when *PIRL* gene was silenced.

In summary, we have identified 134 conserved miRNAs and 35 novel miRNAs from six small RNA libraries of the strawberry cultivar ‘Yanli’ by treatment with *B*. *cinerea* through high-throughput sequencing and predicted their target genes using TargetFinder software. These results indicate the existence of specific miRNAs in the octoploid strawberry ‘Yanli’ treated by *B*. *cinerea*. According to the characteristics of miRNA, wherein they negatively regulate their target genes, down-regulated miRNA could be beneficial to improve the disease resistance of plants. Through analyzing the transient expression of *PIRL* gene in the infected ‘Yanli’ fruits, *PIRL* gene showed the characteristics of resistance to *B*. *cinerea*, and this is of great value to further study the disease resistance mechanism of plants.

## Materials and Methods

### Plant materials and Botrytis Inoculation

The plants of the strawberry (*Fragaria* × *ananassa*) cultivar ‘Yanli’ were maintained in a greenhouse at Shenyang Agricultural University and conventional cultivation and management. The cultivated form was double row per ridge with 90 plants. In the full fruiting period, 450 robust strawberry plants were selected for study. Among them, 360 plants were treated with *B*. *cinerea* and other 90 plants were treated with sterile water.

The *B*. *cinerea* strains were transplanted on the PDA plate for activation, and then the activated strains were incubated in a constant temperature incubator at 25 °C until the conidia grew. Conidia were treated with sterile water, and spore concentrations were measured with a cell count plate, and their concentrations were adjusted to 10^5^·mL^−1^. The healthy strawberry plants of ‘Yanli’ were sprayed with the pathogen suspension in the greenhouse and moisturized with plastic film. After inoculation, the red ripening fruits with the same developmental stage and similar size, were selected and taken from strawberry plants at 48 h (T1), 72 h (T2), 96 h (T3) and 120 h (T4), respectively. The red ripening fruits after sterile water treatment (CK1) and *B*. *cinerea* (CK2) were selected as controls, respectively. The selection criteria about developmental stage and size is the same with the fruits treated by *B*. *cinerea*. Each library processed at least 7 fruits per time, and the experiment was repeated three times. The fruits and *B*. *cinerea* were stored at −80 °C.

### sRNA library construction and high-throughput sequencing

To ensure the use of qualified samples for sequencing, the purity, concentration and integrity of RNA samples were detected. After the samples were qualified, a total amount of 1.5 μg of RNA per fruit sample was used as input material, and the volume was added to 6 µl with water for the RNA sample preparations. Total RNA of *B*. *cinerea* was isolated using TRIzol reagent (Tiangen, Beijing, China) according to the protocol. Libraries were constructed using a small RNA Sample Pre Kit. Because the 5′ end of small RNA had a phosphoric acid group and the 3′ end had a hydroxyl group, T4 RNA ligase 1 and T4 RNA ligase 2 (truncated) were used to connect the connectors at the small RNA 3′ and the 5′ ends, respectively. cDNAs were synthesized using reverse transcription, and PCR amplification was carried out. Gel separation technology was used to screen the target fragments, and then the rubber was cut and recycled as the pieces to obtain small RNA libraries. Sequencing libraries were generated following the manufacturer’s recommendations (Fig. S3). Finally, PCR products were purified, and library quality was assessed on an Agilent Bioanalyzer 2100 system. The purified PCR products were used directly for cluster generation. The clustering of the samples was performed on a cBot cluster generation system using TruSeq PE Cluster Kit v4-cBot-HS (Illumia) according to the manufacturer’s instructions.

### Bioinformatic analyses of sequencing data and target gene prediction of miRNAs

Raw data (raw reads) of fastq format were first processed through in-house Perl scripts. In this step, clean data (clean reads) were obtained by trimming the sequences smaller than 18 nt or longer than 30 nt and removing reads containing adapter, ploy-N and low quality reads from raw data. At the same time, Q20, Q30, GC-content and sequence duplication level of the clean data were calculated. All the downstream analyses were based on clean data with high quality. Using Bowtie tools soft^63^, the clean reads were compared with Silva database, GtRNAdb database, Rfam database and Repbase database for sequence alignment. After filtering out ribosomal RNA (rRNA), transfer RNA (tRNA), small nuclear RNA (snRNA), small nucleolar RNA (snoRNA) and other ncRNA and repeats, the remaining reads were used to detect known miRNAs, and new miRNAs were predicted from miRBase. Randfold tools were used for novel miRNA secondary structure prediction.

Target gene prediction was carried out using TargetFinder software^64^. The total scoring for an alignment was calculated based on the miRNA length, and the sequences were considered to be miRNA targets if the total score was less than 3.0 points (mismatch = 1 and G:U = 0.5)^65^. The homologous sequences of PIRL in *F*. *vesca* and *Arabidopsis* were identified at TAIR (http://www.Arabidopsis.org) and (http://www.ncbi.nlm.nih.gov), and conserved domains of the three amino acids were analyzed by blastp searches at (http://www.ncbi.nlm.nih.gov). Blast software was also used to compare predicted target gene sequences with GO database to obtain annotation information for target genes^66^.

### Quantitative reverse transcription polymerase chain reaction

According to standard protocols^67^, quantitative reverse transcription polymerase chain reaction (qRT-PCR) was performed using stem-loop primers with the TaqMan PCR protocol on an Applied Biosystems 7500 Real-Time PCR System (Applied Biosystems, Foster City, CA, USA). The 20-μl reverse transcription reactions were performed using the AMV reverse transcriptase (Takara, Dalian, China) as described by Li *et al*.^5^. Strawberry 26 s ribosomal RNA (rRNA) was used as an endogenous control, its RT-primer and cycling conditions were different from miRNAs, the reverse transcription of 26 s was performed by using a Takara reverse transcription Kit and was incubated at 37 °C for 2.5 h and 70 °C for 15 min. The program for strawberry 26 s reverse transcription reactions was as described by Li *et al*.^5^. The PCR reaction was performed with the probe RT-PCR Kit (Tiangen, Beijing, China). The TaqMan probe, forward primer and reverse primer sequences are shown in Table S6. The reactions were incubated in a 96-well plate (Applied Biosystems) at 95 °C for 10 min, followed by 40 cycles of 95 °C/1 s and 60 °C/60 s. Three biological replicates were performed, and all reactions were run in triplicate. The averages of each of the six libraries were used as the final result. The quantification of each miRNA relative to the strawberry 26 s rRNA gene was calculated using the Ct (2^−ΔΔCt^) method.

qRT-PCR of the *PIRL* gene was conducted with SYBR Green II (Takara, Dalian, China) using two primers (Table S6) and the following thermal cycling conditions: 95 °C for 10 min, followed by 40 cycles of 95 °C/10 s, 60 °C/1 min. The results were normalized using strawberry 26 s rRNA as the housekeeping gene. Every experiment of qRT-PCR was three biological replicates.

### Cloning of *PIRL* gene

The full-length sequence of the *PIRL* gene was cloned using the PCR primers in Table S6, and the PCR amplifications were carried out in a 20-μl reaction containing 1 μl of cDNA, 1 μl of each primer, 7 μl of ddH_2_O and 10 μl of 2× Es Taq MasterMix (Cwbio, Jiangsu, China). The reaction was incubated at 95 °C for 5 min, followed by 35 cycles of 95 °C/30 s, 62 °C/30 s, 70 °C/1 min 30 s, and 70 °C for 10 min. The amino acid sequence of *PIRL* we cloned was compared with *PIRL* sequence in the strawberry genomic information (*F*. *vesca* (taxid:57918)) from NCBI. The HMM sequence of PIRL domain was downloaded from the Pfam protein family database (http://pfam.xfam.org/) as the query sequence, the BLAST-P alignment and search in the forest strawberry genome (*F*. *vesca* Annotation Release 101) annotated by NCBI database were made by the default setting. Finally, the NCBI CDD database (http://www.ncbi.nlm.nih.gov/Structure/cdd/wrpsb.cgi/) and the Pfam database (http://pfam.xfam.org/) are used to manually identify all derived PIRL sequences to confirm the existence of the complete PIRL domain. Multiple amino acid sequences of strawberry PIRL domain were identified by ClustalX 2.0.12 software and the phylogenetic tree of PIRL from different plants was constructed using MEGA 5 software.

### Vector construction of *PIRL* gene

The specific target fragment without homologous sequence was selected for the construction of the interference vector. Using pRI101-AN vector as the interference vector frame, a segment of intron was added between the enzyme cutting site Sma I and Kpn I, thus a stem loop was formed to silent the target fragment. The primers for forward and reverse fragments of interference vectors are shown in Table S6. The construction of the interference vector was made by the laboratory of the Institute of horticulture and molecular biology in horticulture.

The mutated PCR of *PIRL* gene was performed using mutated primers in Table S6 as described by Dong^68^ due to the existing target sites of miR5290a was contained in *PIRL* sequence^68^. Site directed mutagenesis was used to change the bases of the *PIRL* sequence binding with miR5290a while the amino acids were unchanged (Fig. S4), and the mutant *PIRL* gene was named *mPIRL*. Then the *mPIRL* overexpression vector was constructed using PCR primers as shown in Table S6 by connecting the full-length *mPIRL* gene to pRI101-AN vector.

### Transient expression of *PIRL* gene and Botrytis Inoculation

*Agrobacterium tumefaciens* liquid of *mPIRL* overexpression vector, *PIRL* interference vector, and the pRI101-AN vector was placed in the YEP liquid medium (including Kan 50 mg·L^−1^, Rif 100 mg·L^−1^) at 28 °C, 180 rpm orbital incubator until the OD value was about 0.8. Then bacteria was collected with centrifuge. The white strawberry fruits with the same phenotypes were selected, and then 1 mL syringe suspension diluted by MMA suspension was injected into the bottom of strawberry fruits.

About 500 µL *B*. *cinerea* bacterial fluid with a concentration of 10^5^ mL^−1^ was evenly coated on the front of the fruits when the white fruits entered the ripening period. The fruit phenotypes were observed to determine whether the gene is resistant to disease after three days. The experiment of treatment of transient expression was three biological replicates.

## Electronic supplementary material

Supplemental Information
