# Supplementary material for: Identification and characterization of known and novel microRNAs in strawberry fruits induced by Botrytis cinerea
Source: Sci Rep. 2018 Jul 19;8:10921. doi: 10.1038/s41598-018-29289-7 (PMC6053406; doi:10.1038/s41598-018-29289-7)
Supplement: Supplementary file 1 — Supplemental Information [file 41598_2018_29289_MOESM1_ESM.pdf]

Identification and characterization of known and novel microRNAs in strawberry fruits induced by *Botrytis cinerea*

Yaoxin Liang • Yuhan Guan • Shaoxi Wang • Yanjun Li • Zhihong Zhang • He Li *

fan-novel-001(5p)　5'-AAGTTTGGCTTCGAATCTGGA-3'

5'-CAAGTAAGGTAAGTTTGGCTTCGAATCTGGACTAAGTAACTAAGTAATATCAGATTCCAAGCCAAACTGACCTTACTTGA-3'

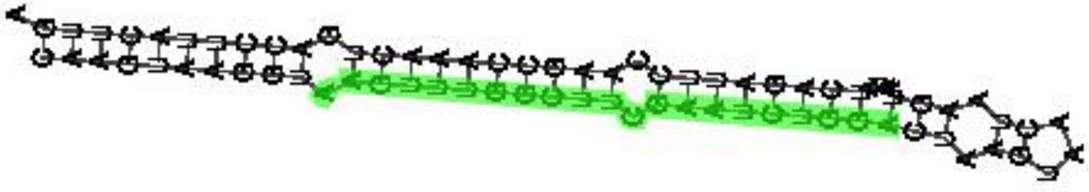

fan-novel-002(3p)　5'- AGGGCGAATACACTCTCAGA-3'

5'-
GGCGTGAAGGCTCTGCGAGTGTCTTCACCTCTGAAGCCTCTTTCTGAAGAAGCTCCTCTTCTTGTTTGGCTTCAGGGCGAATACACTCTCAGACACTAC
ACGC-3'

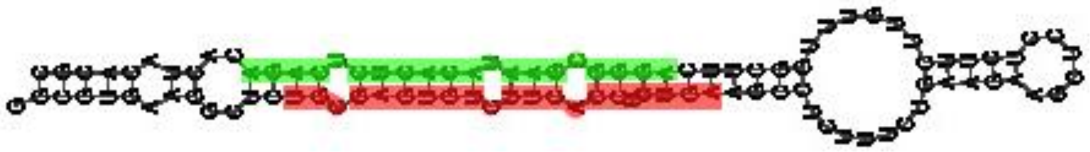

fan-novel-003(5p) 5’-AGTGGTATCAGGGCTATGTT-3’

5’-
GATTCAAACAAGTGGTATCAGGGCTATGTTAAACTAGGATGCATCTTGATCTGGTTGCTTCAAGTCTGACCTAGCTCTGATACCATGTGTTACTGACTTC
A-3’

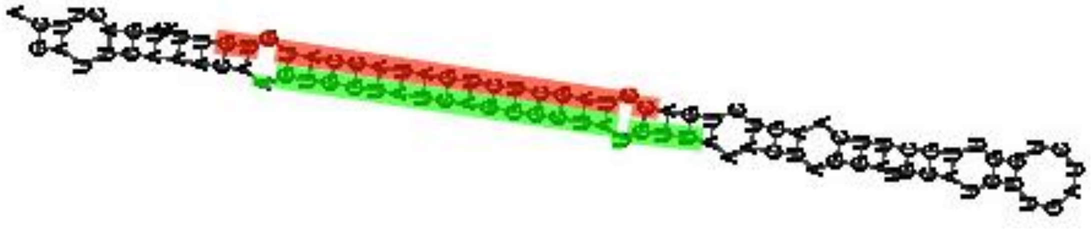

fan-novel-004(3p) 5’- ATTGGATTGAAGGGAGCTCTC-3’

5’-
ATAGAGAGATGAGAGCTTCCTTCGGTCCAAATGCAGGAGTAGAGATGGTGTACACTGCTCCTAATTCATGAATACCTCTGGGTTGCGCAGGATAATGAA
ACACTAATGCGCAGCTTAAGGGTTTGCATGACCTAGGAGCAGAGATTTCCTTCTCATTTCCTTGATTGGATTGAAGGGAGCTCTCATTATTAGTG-3’

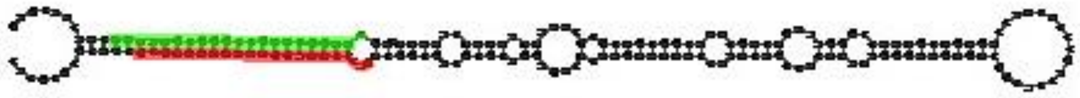

fan-novel-005(3p) 5’-CAGGGCGAATACACTCTCAGA-3’

5’-
GGCGTGAAGGCTCTGCGAGTGTCTTCACCTCTGAAGCCTCTTTCTGAAGAAGCTCCTCTTCTTGTTTGGCTTCAGGGCGAATACACTCTCAGACACTAC
ACGC-3’

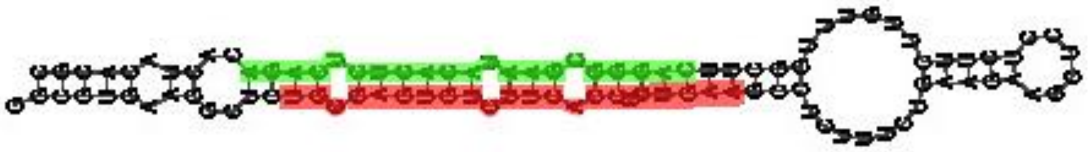

fan-novel-006(5p) 5’-CATGGGAAGTTTGGAAAGAAT-3’

5’-AAGTTTCTGTCATGGGAAGTTTGGAAAGAATTGGTTGTTTGTTTATGACATCAAACTTCTTTCCAATTCCTCCCATGCCAGTGGTTT-3’

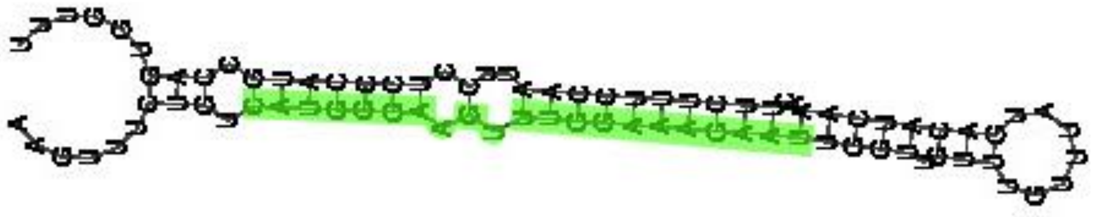

fan-novel-007(3p) 5'-CGGAAAACTGGAATTTGGCGG-3'

5'-
CCCCGACGAGTCCGCCGAATTCCAGTTTTCCGGCCAGATCACCCAAAACTTCAAACAAAGTCAATTTGGCCGGAAAACTGGAATTTGGCGGACTCGC
CTGG-3'

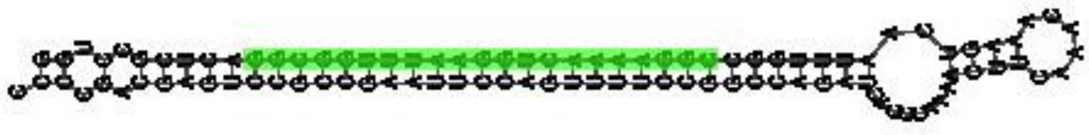

fan-novel-008(3p) 5'- CGGTATAGTCTTTCCTGAGCTCTTG -3'

5'-AGTAGAGACATGAGCCAGGAATGACTTGCCGGCCGCGGGCACATGAGCCGTTGCCGGTATAGTCTTTCCTGAGCTCTTGTTTGCTCTCTT-3'

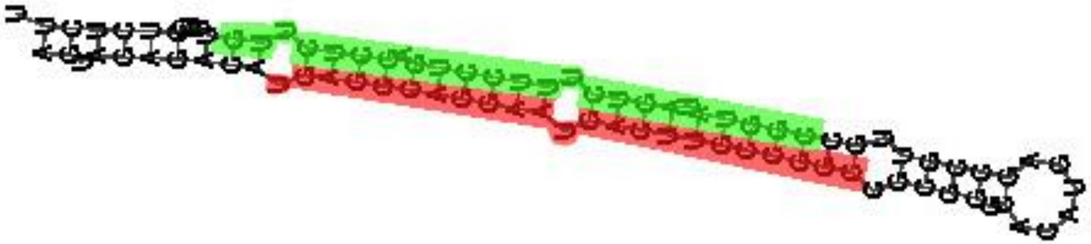

fan-novel-009(3p) 5'-GGAGCGACCTGAGACCACATG-3'

5'-
AAGGGTAGAGGAGTCCCTGAGGAGCGACCTGAGACCACATGTGTGCACCCTCACCACACTCCAAAGTGAGAGTACATAACCACGTGTTCTCAGGTCG

CCCCTGCGAGACTTTCCGTTTACCAC-3’

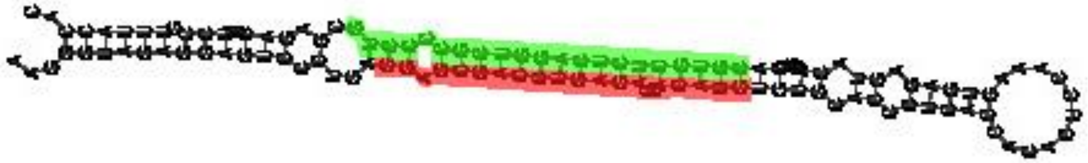

fan-novel-009_1(5p) 5’-CGTGTTCTCAGGTCGCCCCTG-3’

5’-
TTGCATTTCACGTGTTCTCAGGTCGCCCCTGCGGGACTTCCTCTACCACGTAACCGCTCTTATGAAATGATAAGATGAGGAATGTAGAGGTGTGTATATG
TTCACAGCATAAGCCAGCCACAAAGGGTAGAGGAGTCCCTGAGGAGCGACCTGAGACCACATGTGTGCAC-3’

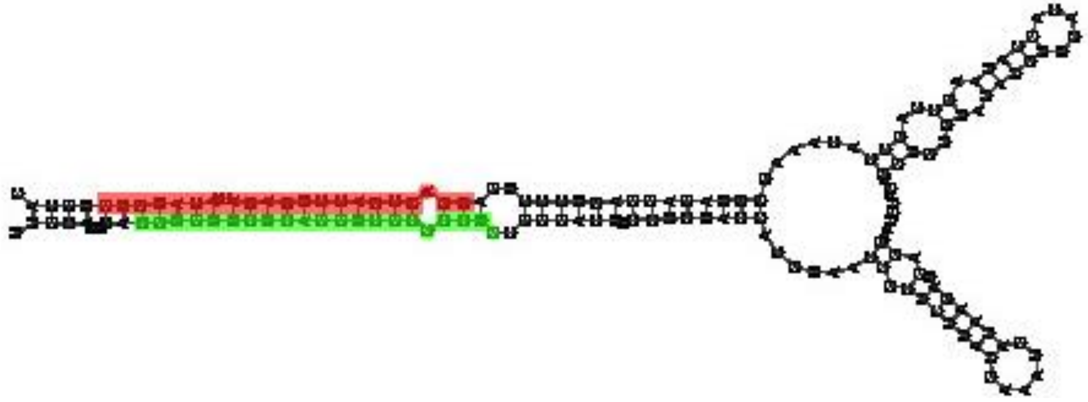

fan-novel-010(3p) 5'-CTTTGATGACTGTGTGATGATG-3'

5'-
CTAGAATTTGATCAAACTCAGCATAAGGGTCTTGACTATCTTCATAATCTGCCCATCTTTATGGTTGATTAGGGGAACTTTGATGACTGTGTGATGATGAT
GATTCTGA-3'

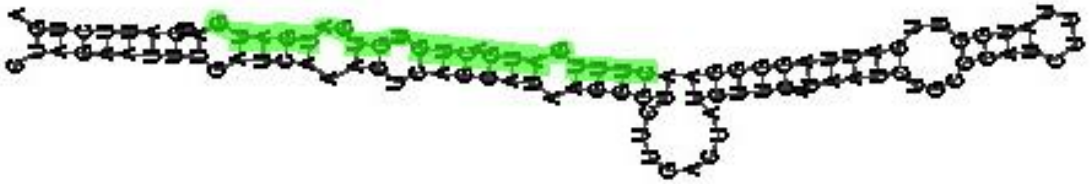

fan-novel-010_1(3p) 5'-CTTTGATGACTGTGTGATGATG-3'

5'-
CTGGAATTTAATCAAACTCAGCATAAAGGTCTTGATTATCTTCATAATCTGCCCATCTTTTTGGTTGATTAGGGGAACTTTGATGACTGTGTGATGATGAT
GATTCTGA-3'

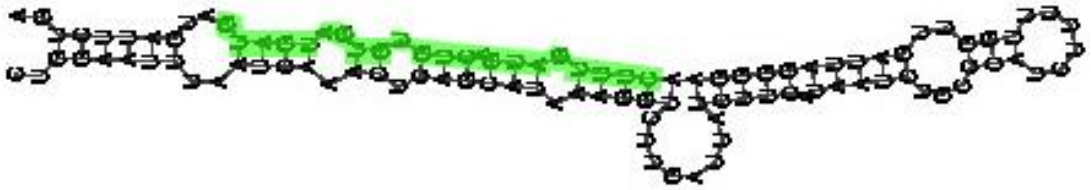

fan-novel-011(3p) 5'-GGAGCGACCTGAGACCACATG-3'

5'-

CCGGTGTTAATTTGCATTTCACGTGTTCTCAGGTCGCCCCTGCGGGACTTCCTCTACCACGTAACCGCTCTTATGAAATGATAAGATGAGGAATGTAGAG
GTGTGTATATGTTCACAGCATAAGCCAGCCACAAAGGGTAGAGGAGTCCCTGAGGAGCGACCTGAGACCACATGTGTGCACCCT-3’

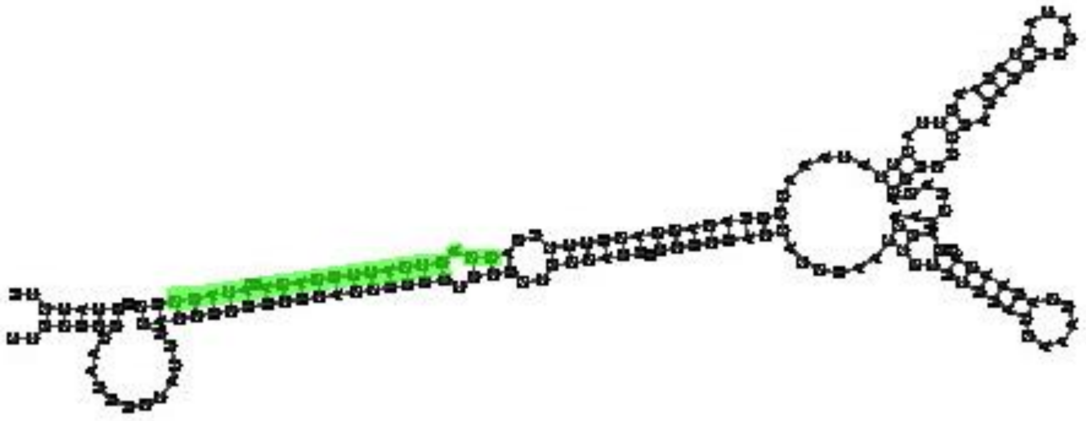

fan-novel-012(5p) 5’-GGAGTGACCTTGAGAACACAGG-3’

5’-
CCATTCCAAAGGAGTGACCTTGAGAACACAGGGTGGTGTTTAGCCACTTACATGTTAATATTAGCGCACCTTGTGTTCCCACGACACCCCTTTGGAACA
C-3’

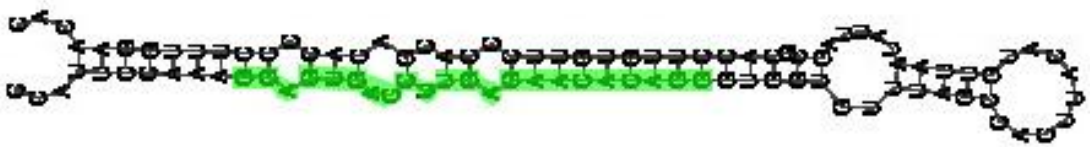

fan-novel-013(5p) 5’-GTCGAGGATCTTGACGAGTTCATC-3’

5’-
TGATTCTTTGCAGTCGAGGATCTTGACGAGTTCATCCAAGCACCATTTGGAGGATGCATAGTTTTCAGAGAGCACAACGATTGATATCTTCGACTCTTCG
ATTGCTTGGAGAAGCGCTGTAGAGATATCTTCTCCTCTTCTGAGCTCGTCATCCATGAATGTGTTGATTCC-3’

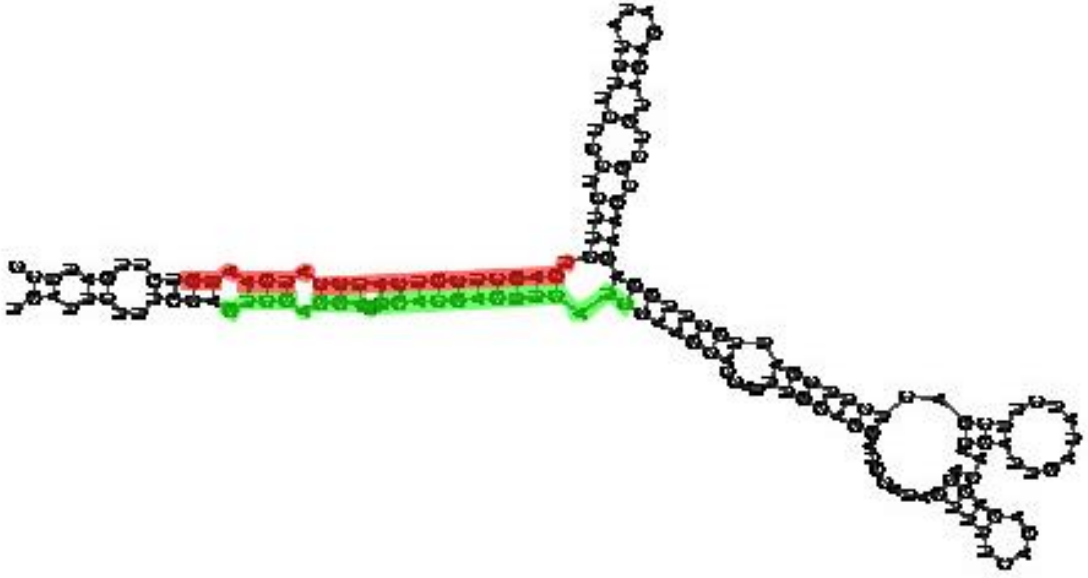

fan-novel-014(3p) 5’-TCATCCAACACATCATCGGCAT-3’ 5’-
GATGTTTACTAAGATGCGGTGACGTGGTGGATGAATTTGCCTTCATAATCAAAGCAAAGGTGCAAACTTGTTTCCACGTTGACAGCAAAGTCACTTGA
AACGTGGAAAACAATTCTTACCTTTCATTTGTAAGTTTTGCAATTTCATCCAACACATCATCGGCATCGTAGGCAAT-3’

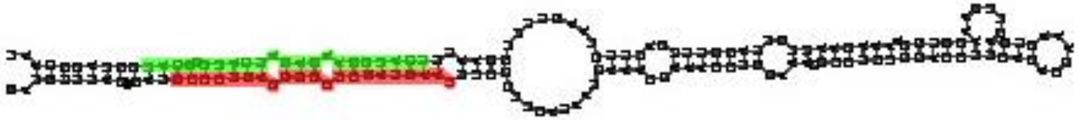

fan-novel-014_1(3p) 5’-TCATCCAACACATCATCGGCAT-3’

5’-

TCAAGGATGTTTACTAAGATGCGGTGACGTGGTGGATGAATTTGCCTTCATAATCAAAGCAAAGGTGCAAACTTGTTTCCACGTTGACAGCAAAGTCA

CTTGAAACGTGGAAAACAATTCTTACCTTTCATTTGTAAGTTTTGCAATTTCATCCAACACATCATCGGCATCGTAGGCAATATCTGCATAT-3’

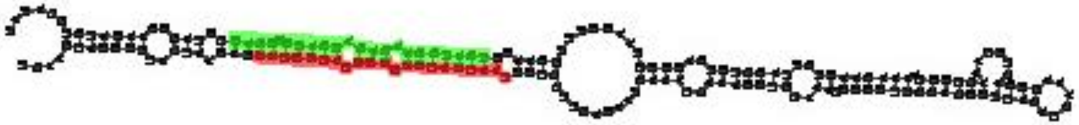

fan-novel-015(3p) 5’-TCCCTATTCCACCTATTCCCCA-3’

5’-

TAGATCCCAAGGAAGTTTTGGGGTATAGGTGGGTAGGATAGAGATCAAAGACTTGTTCTTTCCCTGAACAGTTTCTCTTCCCTATTCCACCTATTCCCCA

TGAGATCCTTTGGTTCCTCC-3’

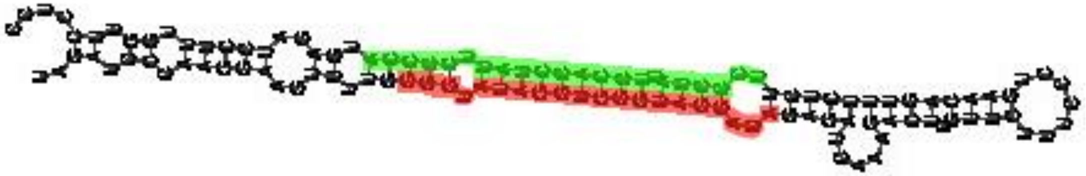

fan-novel-016(3p) 5’-TCCGTTGTAGTCTAGTTGGTC-3’

5’-

AGAGGAACAGGGCAGGCGTCGACTGCAATGGATATGAGAGACGCATAGAAAGAGGAAGACAAACGCTACAACACTGGAAAACCCTCTTTGCTGTGC
CAAAACCAAAAACGGTAGCAAGTAAGTGGACAAATATTGATAACTGTGTTAGTGGACATCGGATCCGTTGTAGTCTAGTTGGTCAGGATATTCG-3’

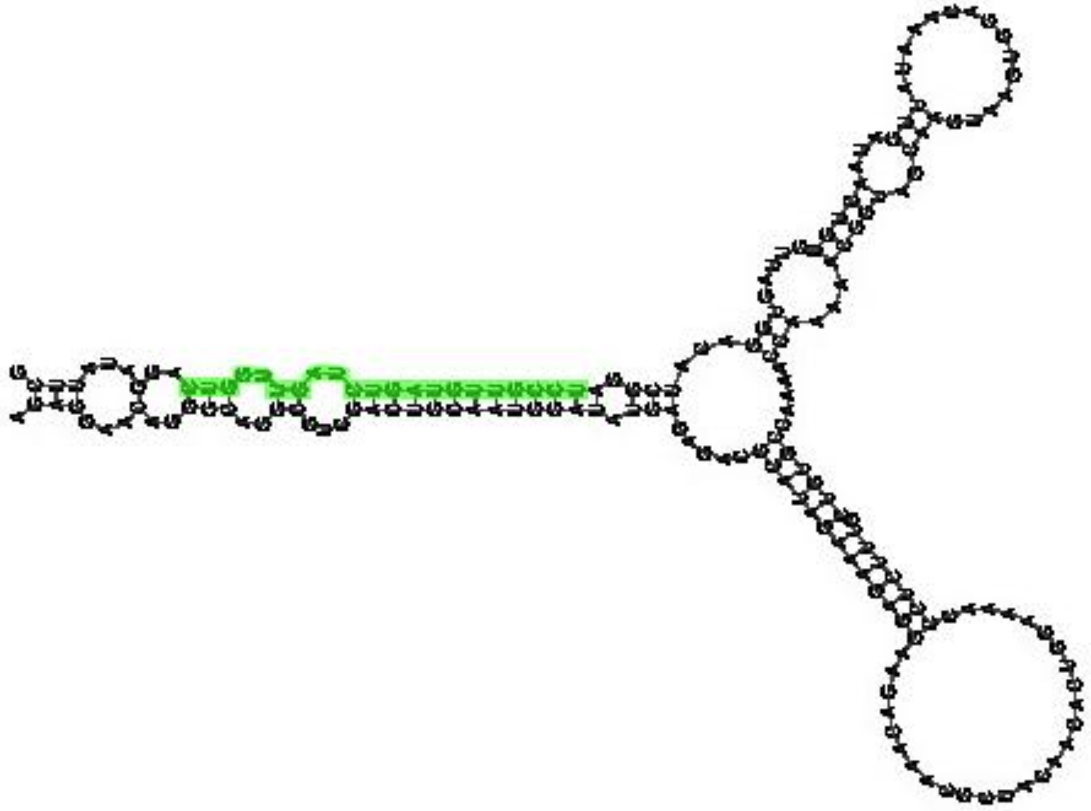

fan-novel-016_1(3p) 5’-TCCGTTGTAGTCTAGTTGGTC-3’

5’-
GCATACTATATAACAGCTAAGACTATAATGATTTGGCTTCCACTAATTCAAGAAGATCATAATCTGAAGATGTAAAAGTAATTAGCTTCAAGCCTTCAAGTTTTTAGCAAACTAAGAACCAATTTAAAGTTTTTAACTTAAGCCTGCTTCCCGTGAATAAAAACAAAGTCAACCTTGAATCACAACTCCGTTGTAGTCTAGTTGGTCAGGATATTCG-3’

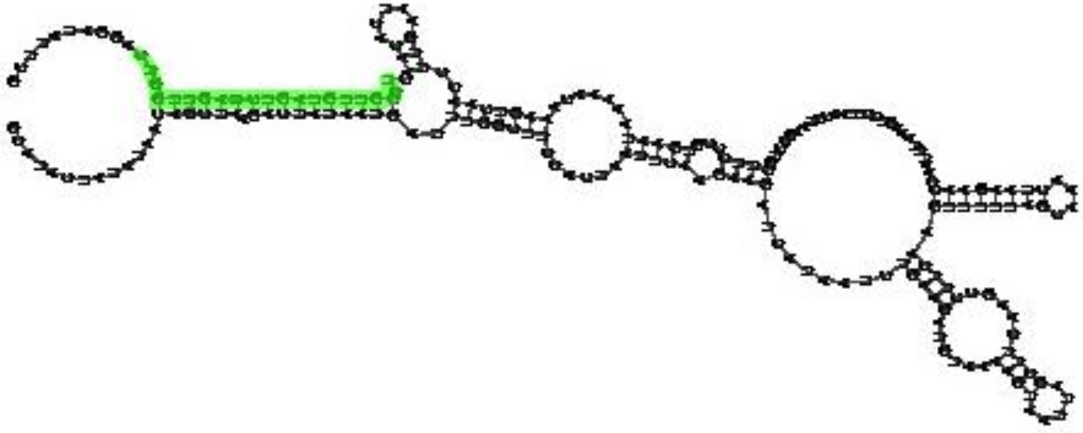

fan-novel-017(5p) 5’-TCGAGGATCTTGACGAGTTCA-3’

5’-
GCTGTTTTGATTCTTTGCAGTCGAGGATCTTGACGAGTTCATCCAAGCACCATTTGGAGGATGCATAGTTTTCAGAGAGCACAACGATTGATATCTTCGACTCTTCGATTGCTTGGAGAAGCGCTGTAGAGATATCTTCTCCTCTTCTGAGCTCGTCATCCATGAATGTGTTGATTCCCTTGTGACGC-3’

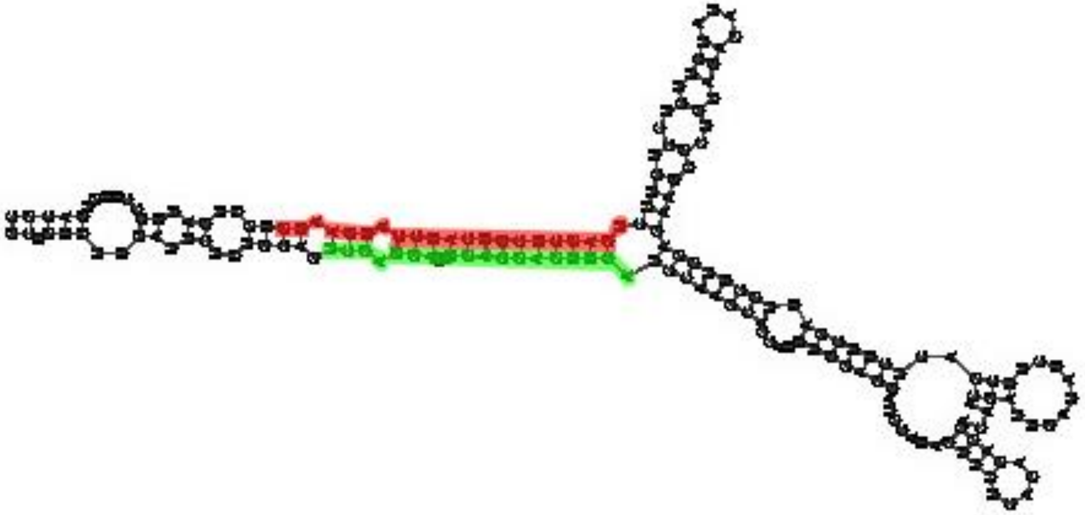

fan-novel-018(5p) 5'-TCTATTCAAAGAGATGACTGTT-3'

5'-
ATTTCATTAGTCTATTCAAAGAGATGACTGTTGAAGAGGAAGAGTCTTAGAGAAAACTCTCAGATTCTTTCTCGACGTTCAGTCTCTCTTTGTATAGACT
AGTGAATT-3'

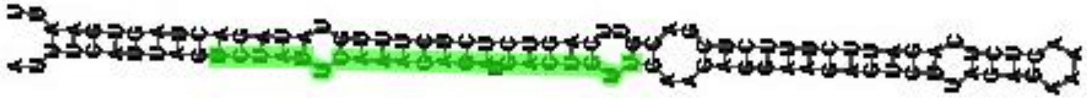

fan-novel-019(3p) 5’-TCTTGCCGATACCTCCCATTCC-3’

5’-
GACTTTGGAAATGTTGTTGGAGTGGGATGGTCGGCAAGAAATGAATAGAGAAAACCCACTCTTGGATTTCTTCCATTTCTTGCCGATACCTCCCATTCC
TGCAACCTTTTCACTACAAG-3’

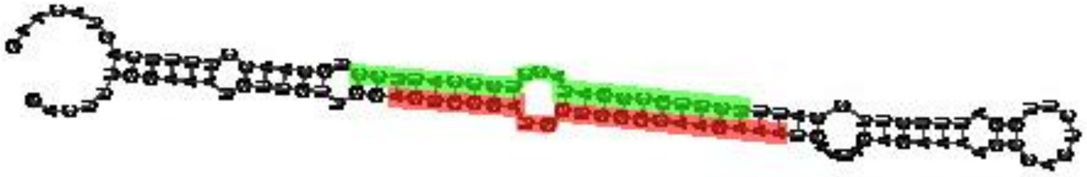

fan-novel-020(3p) 5’-TCTTTCCTAGTCCTGCCATTCC-3’

5’-
GGAGTGGCCGGAAGTTGTGGAGTGGAGTTCTGGGAAAGAAGACCATGGTTTTTCTCGAAGTAATTTGGGAAAAAAACATTGGTTCTTTCCTAGTCCTG
CCATTCCTTGATTTCCTGTCATGCATC -3’

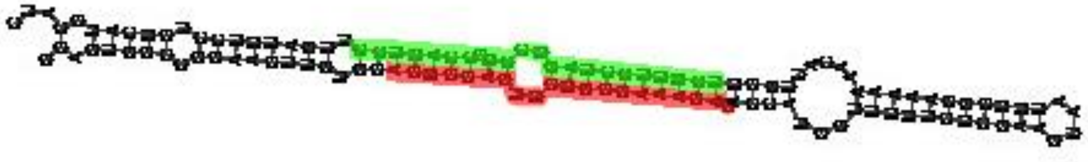

fan-novel-021(3p) 5’-TCTTTCCTATTCCTCCCATCCC-3’

5’-
TGTGAGGAAGTTGTAGTTGGGATGGGAGGCTAGGGAAGAAATTACTGGAAACTCATCCTAATTTCTTTCCTATTCCTCCCATCCCTCCTCAATTTCCCAA
GGTTT-3’

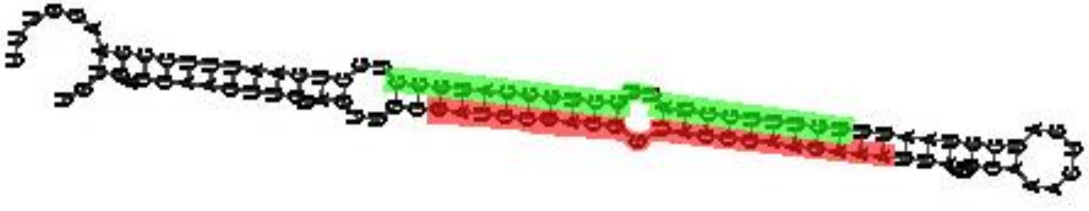

fan-novel-022(3p)   5’-TGAAGTGGGATTTGGCGAATT-3’

5’-
CTAGGGTCTTCTATCAGCGATTCGTCAGATTCCAATTCAAACTGAGTAGTCGGATGGTCGACTGTAAAGAGTTTGAAGTGGGATTTGGCGAATTGAGGG
TCAAGATTAATAGAG-3’

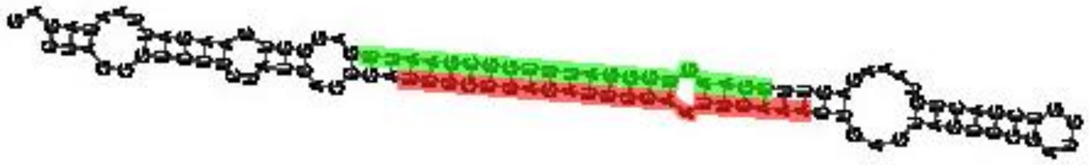

fan-novel-022_1(3p) 5’-TTCGTCAGATTCCAATTCAAA-3’

5’-CTATCAGCGATTCGTCAGATTCCAATTCAAACTGAGTAGTCGGATGGTCGACTGTAAAGAGTTTGAAGTGGGATTTGGCGAATTGAGGGTCA-3’

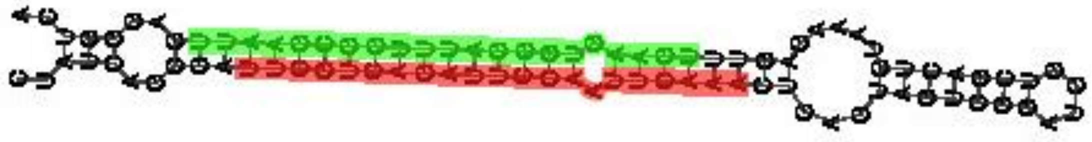

fan-novel-023(5p) 5’-TGAATTGGGATTTGGCGAATT-3’

5’-
GAAGAGAGTCTGAATTGGGATTTGGCGAATTGAGGGTCGCGATTAATAGATGTCCATCGTTTTGAGACACAGGAGAAGCGGGTTCCTTGTTTTGATGG
GTAGTCCGGGAGGATGAAGAGAATGAGAGCTATCATAATTACTATAGATATATTCATGGAAAACGTTTACTCTATTGTGTTCTACGTATATATGTATGTATT
GCTTCATTTGTTTTGTTTGTATTATACAGGTCGGAAATGGTTTGGTTTCAGCCTTTCAGAATGTGAGCAACCAGCTCTTGCTTTGCCAAAACTTTTTTTAT
CCGTTGCGGCTGCGTTTCT-3’

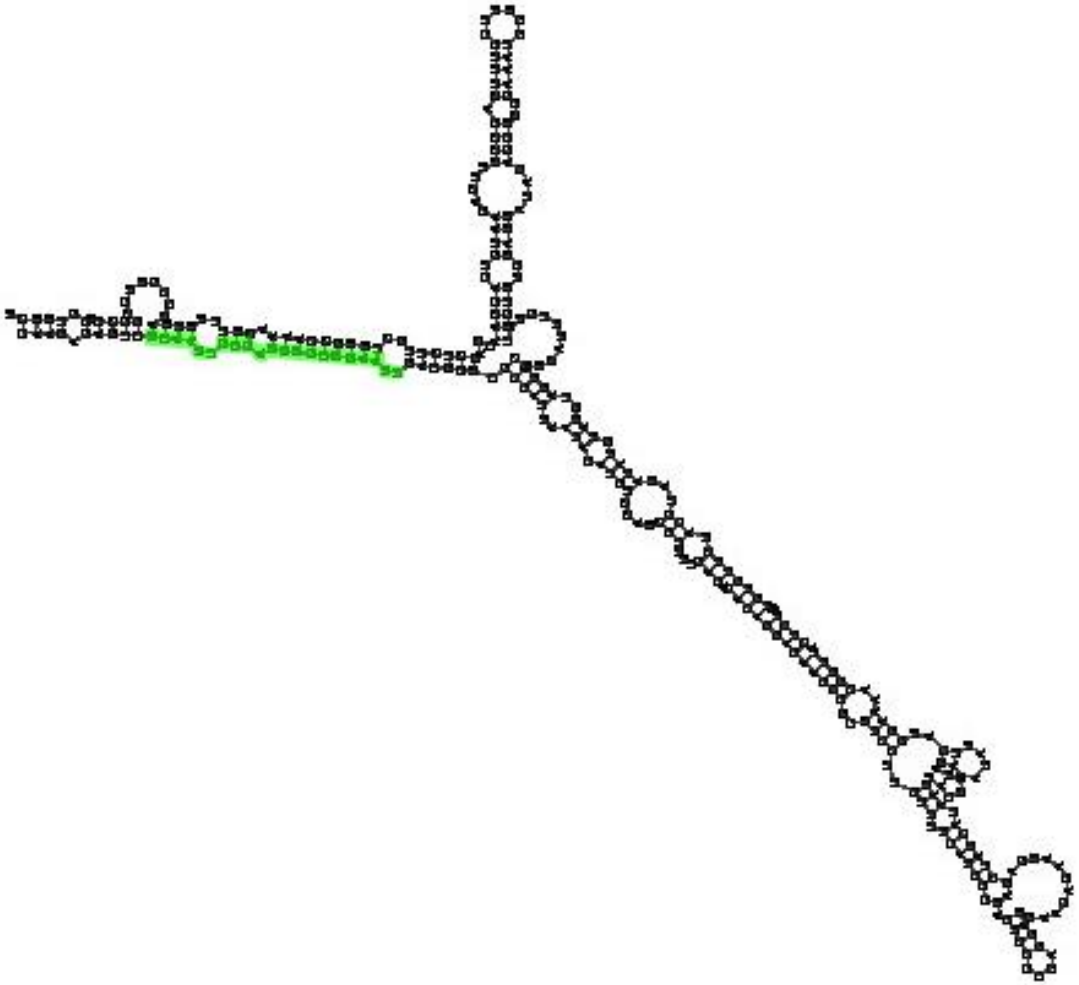

fan-novel-023_1(3p) 5'-TGAATTGGGATTTGGCGAATT-3'

5'-
TTCGATCAGCAATTCGTCACATTCCAGTTCAAACTGAGCAGCAGTCGGATATGGCCGACGTACTGAAGAGAGTCTGAATTGGGATTTGGCGAATTGAG
GGTCGCG-3'

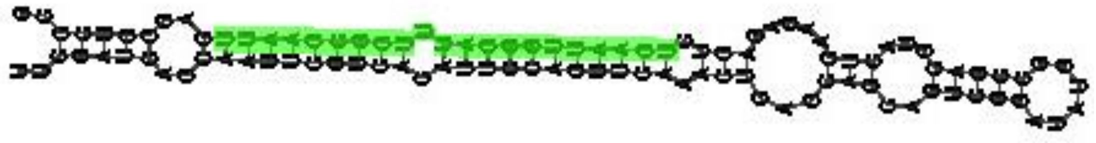

fan-novel-024(5p) 5'-TGACAGAAGAGAGTGAGCTC-3'

5'-
CTAAAGGGGCTGACAGAAGAGAGTGAGCTCAGATTACACATTTGTAATGAGTACTAAGTTTATCATCTTCCTCATGCTACATATTTGTGTAATGTGTGCT
CACTTCTCTATCTGTCAGCTGCAGATC-3'

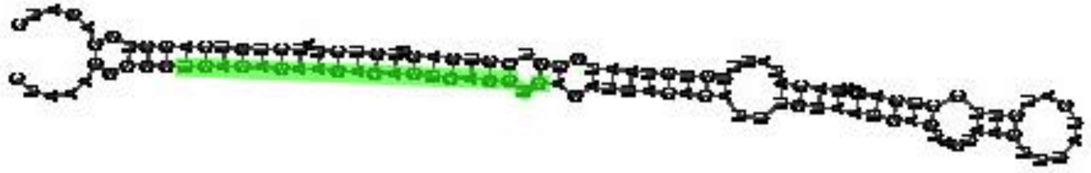

fan-novel-025(3p) 5'-TGGAGCCTGCGAGGGGGAATG-3'

5'-
AAGGCATTGGTTGATCACTCTCCCTCAAGGGCTTCTCGCCAAGGTGAAGTATTATATCCTAGGTCGTCCCCTTCTTAATTCTAAGGGAGACTTGGGGTAC

TTACACAAGGTTGTGGAGCCTGCGAGGGGGAATGACCAAGCTTC-3’

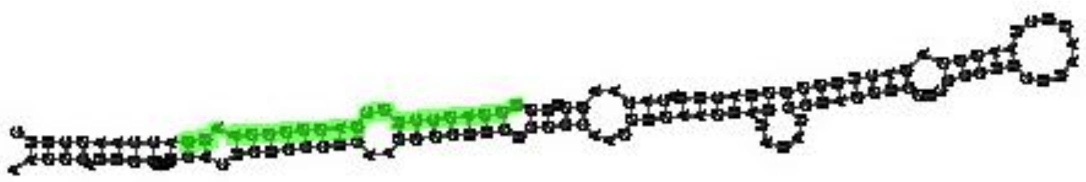

fan-novel-026(3p) 5’-TGGGATTGGGCGAATTTTGGT-3’

5’-
ATCGGTGACGTCTTCCGCATCAAAATTGGCCACCTCCCAATTAAAACTTAGAGTTTGAGATGCTCAAATCCTAGTTTGAACTGGGATTGGGCGAATTTTGGTCGAGATTGATCTACTATGCT -3’

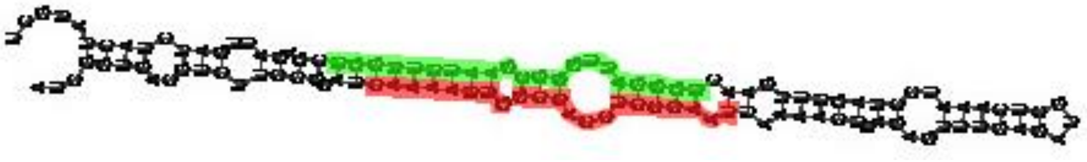

fan-novel-027(3p) 5’-TGGGATTTGGCGAATTGTGGT-3’

5’-
GGGTGAAGTCCTGCACCACAATTGGCCACCTCCCAATTGAAACTTAGGGTTTTAGATGCTCAAATCTTAGTTTGAAATGGGATTTGGCGAATTGTGGTCGAGATCACC-3’

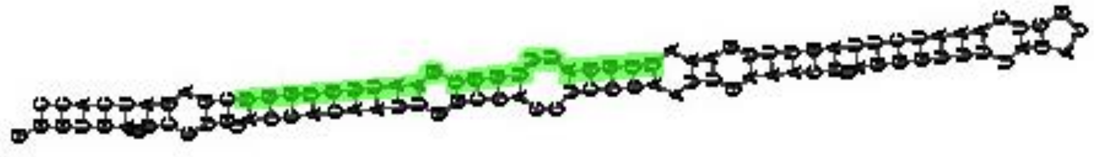

fan-novel-028(3p) 5'-TTACCCTTGCAATATCCGTTG-3'

5'-
AGTTGTAATTGCGTTGGTCAACGGATATTGCTAGGGTAGAGAAGTCAACAAGGCTAAGTTAGCCTTAGGGCTTAGGAACATATTTACCCTTGCAATATCCGTTGATCAATATAATTACTTGTAC -3'

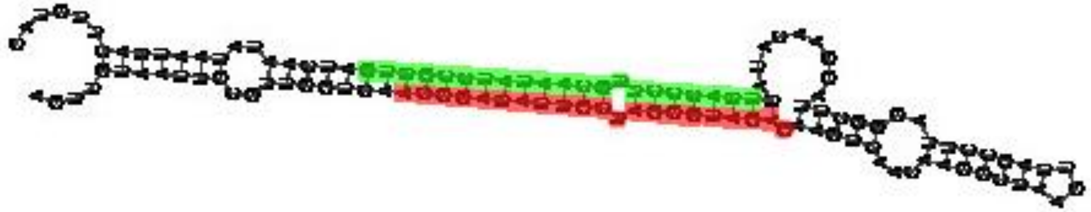

fan-novel-029(5p) 5'-TTCGTGATCTGCGAAAGGCTC-3'

5'-
CAAGAGAAAACTCTCCTGTCTTCGTGATCTGCGAAAGGCTCCTCACAGTCATTGCCCATCTGATTGTGAGACTCAGGCACCTGTCGTAGATCATGAAGATGGGGTATCTTTCTCAAAGC-3'

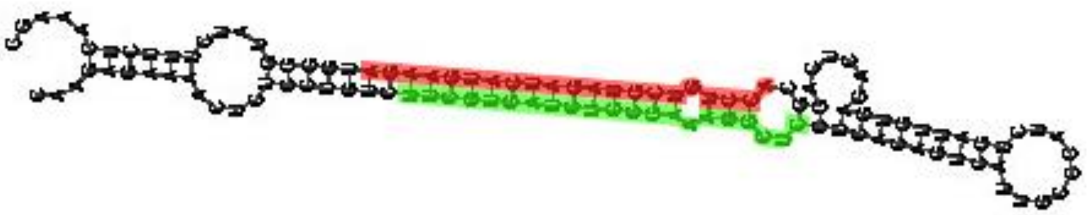

fan-novel-030(3p) 5’-TTGCGGTCTTGTCTCTTCCAAT-3’

5’-

CCCTAATTCGACTGGAAAAAGAAAGACCGCAAAGAAGCCCAGGACTTTCGCCGCCCCTTTCTTCACAGAAAGGCTGGCTCTTGGCGCCCGGGAAATC
CTTTGCGGTCTTGTCTCTTCCAATTCGAATTCGG-3’

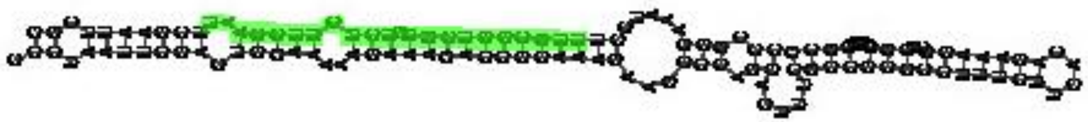

fan-novel-031(3p) 5’-TTGGACTGAAGGGAGCTCCCC-3’

5’-

TGTATCAAGGGAAGGGAGCTTTCTTCAGTCCACTCATGGGTGGCAGTAGGATTTCAATTAGCTGCCGACTCATTCATCCAAATACTGGGTTATTAGATCA
ATCAAGACAAACCCAGTAAATGCGTGAATGATGCGGGAGACAAATTGGATCTTAAGCTTCCTGTACTTGGACTGAAGGGAGCTCCCCTATCTAGTAC-3’

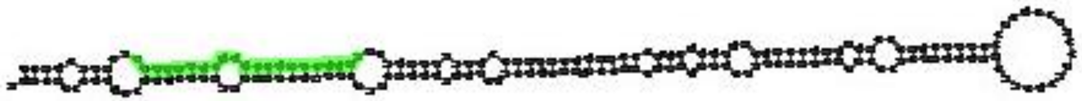

fan-novel-032(3p) 5'-TTGGACTGAAGGGAGCTCCCT-3'

5'-
TATATTAGCAAGAGAGCTTTCTTCAGTCCACTCATGGAAGGGCAAAGGGTTTGGATTAACTGCCAACTCATTCATTCAAACACAGTAGAGAAAACGGA
AGGAAACTCTGCCGCGAATGTGTGAATGATTTGGGAGGTAAATTTCATCCTCTTCTTCTCTGTGCTTGGACTGAAGGGAGCTCCCTTCTTTAATGT-3'

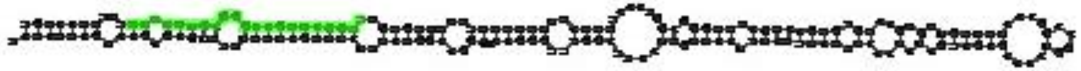

fan-novel-033(3p) 5'-TTGGACTGAAGGGAGCTCCTC-3'

5'-
GAGAGAGAAGGGAGGGAGAGCTTTCTTCAGTCCACTCATGGATAGGATTGGATGGGGTTTGTTGAAGTAACTGCCTACTCATTCATTCAAGCACTCCT
CAGTATCTTATGTTTGCTACTGTGATTGATTGTGTGAATGAAGCGGGAGTTAATTTACATCCCTCTTTCTCTTTGCTTGGACTGAAGGGAGCTCCTCCACT
TTTCCT-3'

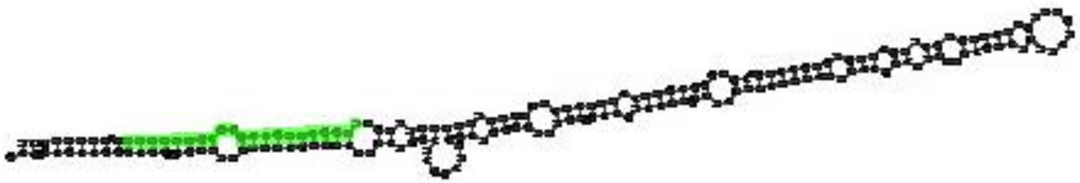

fan-novel-034(5p) 5’-TTGGAGAGAGAGTAGACAATG-3’ 5’-AGTTTCAGAAGCCAAGGTCCTTGGAGAGAGAGTAGACAATGTGGCGAAGTGCCGGTGCGAAGTTCCACATTGTCTACTCTCTCTCGAAAGGACCTCGGCCTTCTGGATTCG-3’

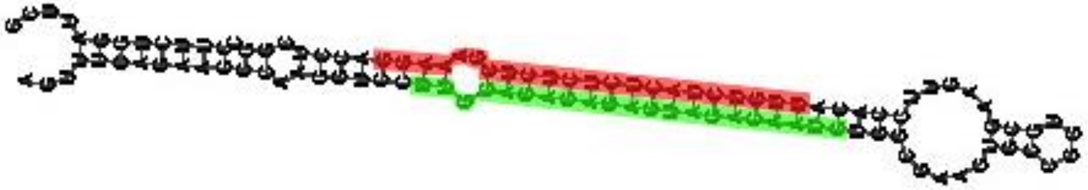

fan-novel-035(3p) 5’-TTGTCTACTCTCTCTCGAAAGG-3’
5’-AAGCCAAGGTCCTTGGAGAGAGAGTAGACAATGTGGCGAAGTGCCGGTGCGAAGTTCCACATTGTCTACTCTCTCTCGAAAGGACCTCGGCCT-3’

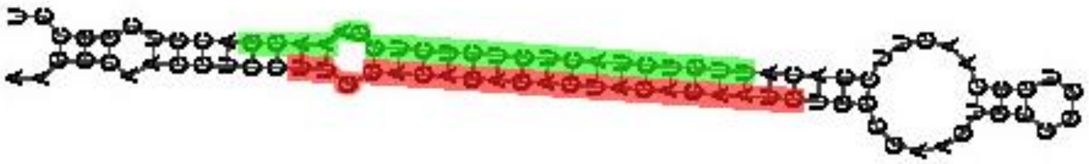

Figure S1 Second structures of novel potential miRNAs in infected strawberry fruits ‘Yanli’ Precursor sequences for novel miRNA from strawberry were shown in black letters with miRNA and miRNA* (The sequence complementary to miRNA in the fold-back structure) sequences highlighted in green and red, respectively. Precursor secondary structures were produced using the mfold software (http://mfold.bioinfo.rpi.edu/).

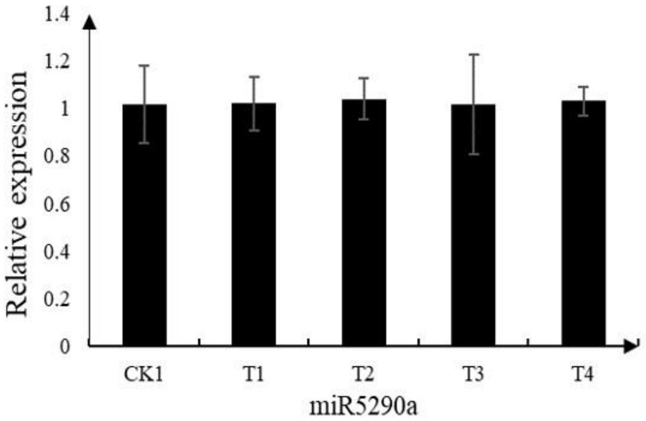

Figure S2 qRT-PCR verification of miR5290a of 'Yanli' strawberry fruits treated with sterile water. CK1, T1, T2, T3, and T4 indicated that after the treatment with sterile water, red ripening fruits were chose and took at 0 h, 48 h, 72 h, 96 h and 120 h, respectively.

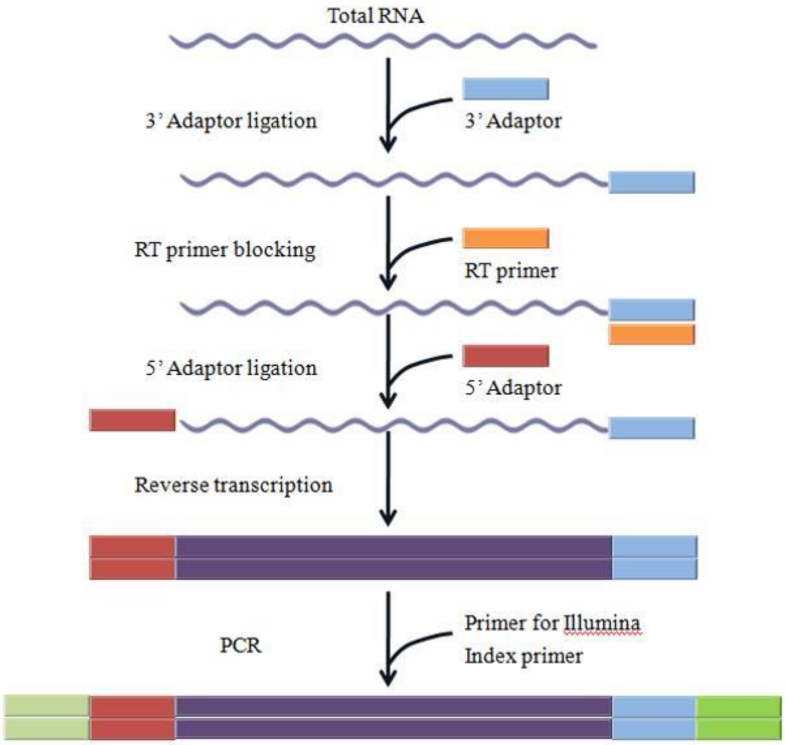

Figure S3 Library construction flow chart.

CTC ATC GAC TCC TCT CTC TCC AA------Original sequence

| | | | | | |

CTG ATT GAT TCG TCG CTG TCG AA------Mutation sequence

| | | | | | |

L I D S S L S ------Amino acid sequence

Figure S4 Base sequence and amino acid sequence before and after mutation of miR5290a and *PIRL* gene binding sites.

Table S1: Conserved strawberry miRNA in six libraries

miRNA family	Name	Sequence (5′-3′)	Length(nt)	Actual MiR read						Normalized MiR read						Ratio	Conserved in other plants							The fan-miRNA location in *F.vesca* genome
				T0	T1	T2	T3	T4	CK	T0	T1	T2	T3	T4	CK	T4/T0	ath	osa	ptc	vvi	ctr	csi	mdm	
miR156	miR156a	UUGACAGAAGAGAGUGAGCAC	21	82714	218466	112806	120253	41963	0	5474.02	16898.79	9054.1	10004.59	2163.67	0	0.4	++	++	++	++	++	++	++	LG2:21698036:21698291:- 256(nt) -51.80(kcal/mol)<br>LG3:29883620:29883738:+ 119(nt) -58.33(kcal/mol)<br>LG3:29884152:29884255:+ 104(nt) -53.50(kcal/mol)<br>LG3:5985875:5986158:- 284(nt) -75.20(kcal/mol)
	miR156b	UUGACAGAAGAGAGUGAGCAU	21	1629	4760	2865	3340	919	0	107.81	368.2	229.95	277.88	47.38	0	0.44	+	+	+	+	+	+	+	no location in *F.vesca* genome
	miR156c	UUGCCAGAAGAGAGUGAGCAC	21	46	2023	984	75	6	0	3.04	156.48	78.98	6.24	0.31	0	0.1	+	+	+	+	+	+	+	no location in *F.vesca* genome
	miR156d	UUGACAGAAGAGAGUGAGCAA	21	557	1637	730	745	92	0	36.86	126.63	58.59	61.98	4.74	0	0.13	+	+	+	+	+	+	+	no location in *F.vesca* genome
	miR156e	UGACAGAAGAGAGUGAGCUC	20	662	1646	776	324	61	0	43.81	127.32	62.28	26.96	3.15	0	0.07	+	+	+	+	+	+	+	LG1:192269:192395:+ 127(nt) -56.50(kcal/mol)
	miR156f	UUGGCAGAAGAGAGUGAGCAC	21	397	1311	609	598	192	0	26.27	101.41	48.88	49.75	9.9	0	0.38	+	+	+	+	+	+	+	no location in *F.vesca* genome
	miR156g	UUGACAGAAGAGAGUGAGCGC	21	437	1439	720	534	143	0	28.92	111.31	57.79	44.43	7.37	0	0.25	+	+	+	+	+	+	+	no location in *F.vesca* genome
miR157	miR157	UUGACAGAAGAUAGUGAGCAC	21	6513	14823	13533	11123	4243	0	431.03	1146.59	1086.19	925.39	218.77	0	0.51	+	-	-	-	-	-	-	no location in *F.vesca* genome

miR159	miR159a	UUUGGAUUGAAGGGAGCUCUA	21	163025	457795	593466	62731	28360	13	10789	35411.38	47633.12	5218.98	1462.28	0.69	0.14	++	+	++	++	-	++	+	LG5:10261604:10261801:- 198(nt) -89.80(kcal/mol)
	miR159b	UUUGGAUUGAAGGGAGCUCUC	21	2795	10892	16269	1713	791	0	184.97	842.52	1305.79	142.52	40.79	0	0.22	+	+	+	+	-	+	+	no location in *F.vesca* genome
	miR159c	UUUGGAUUGAAGGGAGCUCUG	21	1286	6662	9022	251	32	0	85.11	515.32	724.13	20.88	1.65	0	0.02	+	++	+	+	-	+	+	no location in *F.vesca* genome
	miR159d	UUUGGAUUGAAGGGAGCUCCA	21	1926	6328	9839	817	405	0	127.46	489.48	789.7	67.97	20.88	0	0.16	+	+	+	+	-	+	+	no location in *F.vesca* genome
	miR159e	UUUGGAUUGAAGGGAGCUCUU	21	3010	7218	9182	943	622	0	199.2	558.33	736.97	78.45	32.07	0	0.16	++	+	+	+	-	+	+	no location in *F.vesca* genome
	miR159f	CUUGGAUUGAAGGGAGCUCUA	21	622	2442	2921	199	74	0	41.16	188.89	234.45	16.56	3.82	0	0.09	+	++	+	+	-	+	+	no location in *F.vesca* genome
	miR159g	UUUGGAUUGGAGGGAGCUCUA	21	690	2160	2578	222	61	0	45.66	167.08	206.92	18.47	3.15	0	0.07	+	+	+	+	-	+	-	no location in *F.vesca* genome
	miR159h	UUUGGAUUGAAGGGGGCUCUA	21	807	2143	2564	291	98	0	53.41	165.77	205.79	24.21	5.05	0	0.09	+	+	+	+	-	+	-	no location in *F.vesca* genome
	miR159i	UUUGGAUUGAGGGGAGCUCUA	21	545	1891	2456	208	71	0	36.07	146.27	197.12	17.3	3.66	0	0.1	+	+	+	+	-	+	-	no location in *F.vesca* genome
	miR159j	UUUCGAUUGAAGGGAGCUCUA	21	12	1906	2316	14	0	0	0.79	147.43	185.89	1.16	0	0	0	+	+	+	+	-	+	-	no location in *F.vesca* genome
	miR159k	UUUGGAUUGAAGGGAGCCCUA	21	891	2800	3923	248	72	0	58.97	216.59	314.87	20.63	3.71	0	0.06	+	+	+	+	-	+	-	no location in *F.vesca* genome
	miR159l	UUUGGGUUGAAGGGAGCUCUA	21	725	1732	2236	204	67	0	47.98	133.97	179.47	16.97	3.45	0	0.07	+	+	+	+	-	+	-	no location in *F.vesca* genome
	miR159m	AUUGGAUUGAAGGGAGCUCUA	21	352	2058	2253	355	65	0	23.3	159.19	180.83	29.53	3.35	0	0.14	+	+	+	+	-	+	+	no location in *F.vesca* genome
	miR159n	UUCGGAUUGAAGGGAGCUCUA	21	398	1241	1544	140	49	0	26.34	95.99	123.93	11.65	2.53	0	0.1	+	+	+	+	-	+	-	no location in *F.vesca* genome
	miR159o	UUUAGAUUGAAGGGAGCUCUA	21	91	1121	1338	31	6	0	6.02	86.71	107.39	2.58	0.31	0	0.05	+	+	+	+	-	+	-	no location in *F.vesca* genome

	miR159p	UCUGGAUUGAAGGGAGCUCUA	21	342	970	1304	109	37	0	22.63	75.03	104.66	9.07	1.91	0	0.08	+	+	+	+	-	+	-	no location in *F.vesca* genome
	miR159q	UUUGGAUUGAAGGGAGCUCG	20	311	1203	1452	219	64	0	20.58	93.05	116.54	18.22	3.3	0	0.16	+	+	+	+	-	+	+	no location in *F.vesca* genome
miR162	miR162	UCGAUAAACCUCUGCAUCCAG	21	2839	2536	2524	91	59	0	187.89	196.16	202.58	7.57	3.04	0	0.02	++	++	++	++	-	++	++	LG5:8224750:8224870:- 121(nt) -44.20(kcal/mol)
miR164	miR164	UGGAGAAGCAGGGCACGUGCA	21	2752	15480	17513	1173	624	0	182.13	1197.41	1405.64	97.59	32.17	0	0.18	++	++	++	++	++	++	++	LG2:16008062:16008160:- 99(nt) -51.70(kcal/mol)<br>LG6:7425147:7425314:- 168(nt) -59.90(kcal/mol)
miR166	miR166a	UCGGACCAGGCUUCAUUCCCC	21	21582	52767	61916	18875	5854	11	1428.3	4081.64	4969.54	1570.33	301.84	0.59	0.21	++	++	++	++	++	++	++	LG2:12452993:12453096:- 104(nt) -61.90(kcal/mol)<br>LG4:2653251:2653368:- 118(nt) -48.70(kcal/mol)<br>LG5:5707593:5707760:- 168(nt) -67.20(kcal/mol)<br>LG7:1205656:1205830:- 175(nt) -59.00(kcal/mol)<br>LG7:20072843:20072980:- 138(nt) -51.26(kcal/mol)
	miR166b	UCGGACCAGGCUUCAUUCCCU	21	1750	4191	4916	1585	222	0	115.82	324.18	394.57	131.87	11.45	0	0.1	+	++	+	++	+	++	+	no location in *F.vesca* genome

	miR166c	UCGGACCAGGCUUCAUUCCCA	21	355	1062	1339	417	39	0	23.49	82.15	107.47	34.69	2.01	0	0.09	+	+	+	++	+	++	+	no location in *F.vesca* genome
miR167	miR167	UGAAGCUGCCAGCAUGAUCUG	21	17056	32206	37945	2387	502	0	1128.77	2491.2	3045.56	198.59	25.88	0	0.02	++	++	++	++	++	++	+	LG3:7735778:7736045:- 268(nt) -80.97(kcal/mol)
miR319	miR319a	UUGGACUGAAGGGAGCUCCUC	21	8631	18382	12719	3878	847	0	571.2	1421.89	1020.86	322.63	43.67	0	0.08	+	+	++	++	+	++	+	LG7:18365232:18365436:+ 205(nt) -81.51(kcal/mol)
	miR319b	UUGGACUGAAGGGAGCUCCCC	21	6145	21260	14263	5050	2283	0	406.68	1644.5	1144.79	420.14	117.71	0	0.29	+	+	++	+	++	+	+	LG5:2300704:2300900:- 197(nt) -86.87(kcal/mol)
miR395	miR395	CUGAAGUGUUUGGGGGAACUC	21	2345	871	1637	189	180	0	155.19	67.37	131.39	15.72	9.28	0	0.06	++	+	++	++	-	++	++	no location in *F.vesca* genome
miR396	miR396a	UUCCACAGCUUUCUUGAACUU	21	17778	21375	22110	2350	2302	0	1176.55	1653.4	1774.61	195.51	118.69	0	0.1	++	++	++	++	-	+	++	LG1:2526788:2526904:- 117(nt) -50.40(kcal/mol)
	miR396b	UUCCACAGCUUUCUUGAACUG	21	8522	13142	13457	2595	863	0	563.99	1016.56	1080.09	215.89	44.5	0	0.08	++	++	++	++	-	++	++	LG0:9235898:9236019:+ 122(nt) -44.20(kcal/mol) LG1:2522058:2522180:+ 123(nt) -59.70(kcal/mol) LG3:1706980:1707072:- 93(nt) -45.30(kcal/mol)
	miR396c	UUCCACAGCUUUCUUGAACUC	21	927	1278	1656	183	118	0	61.35	98.86	132.91	15.22	6.08	0	0.1	+	+	+	++	-	+	+	no location in *F.vesca* genome
miR398	miR398	GGUGCGACCUGAGAUCACAUG	21	19585	50715	13120	0	1536	0	1296.14	3922.91	1053.05	0	79.2	0	0.06	-	-	+	-	-	-	-	no location in *F.vesca* genome
miR399	miR399	UGCCAAAGGAGAGUUGCCCUG	21	644	916	1506	43	44	0	42.62	70.85	120.88	3.58	2.27	0	0.05	++	++	+	++	-	++	++	LG5:958268:958392:- 125(nt) -55.00(kcal/mol)

miR408	miR408	ACAGGGACGAGGUAGAGCAUG	21	4483	9278	9460	1257	1222	0	296.69	717.67	759.28	104.58	63.01	0	0.21	-	+	-	-	-	-	+	LG1:12223080:12223380:- 301(nt) - 82.30(kcal/mol)
miR477	miR477	CUCUCCCUCAAGGGCUUCUC	20	813	1681	1170	253	279	0	53.8	130.03	93.91	21.05	14.39	0	0.27	-	-	-	-	-	+ +	+	LG5:2676778:2676912:+ 135(nt) - 60.50(kcal/mol)
miR482	miR482	UCUUUCCAAUUCCUCCCAUGCC	22	2098	5187	7002	279	61	0	138.85	401.23	562	23.21	3.15	0	0.02	-	-	-	-	-	-	-	LG4:464029:464131:- 103(nt) - 37.90(kcal/mol)
miR868	miR868	UAUGUCAUAACAGUAGACUACA	22	0	0	0	0	6167	0	0	0	0	0	317.98	0	>> 1	-	-	-	-	-	-	-	no location in *F.vesca* genome
miR948	miR948	UCAGGUUGAUCGUGGAUCCUG	21	0	0	0	0	4834	0	0	0	0	0	249.25	0	>> 1	-	-	-	-	-	-	-	no location in *F.vesca* genome
miR1026	miR1026	UUGUGAAAUGACUUGAGAGGUG	22	0	547	1261	0	0	0	0	42.31	101.21	0	0	0	Both 0	-	-	-	-	-	-	-	no location in *F.vesca* genome
miR1122	miR1122	UAAGAGACAUCCGUUUUGGACU	22	0	0	0	4602	0	0	0	0	0	382.87	0	0	Both 0	-	-	-	-	-	-	-	no location in *F.vesca* genome
miR1511	miR1511a	AACCGGCUCUGAUACCAAUUG	21	917367	1641217	1628276	353514	130689	71	60711.38	126951.48	130689.66	29411.01	6738.5	3.79	0.11	-	-	-	-	-	-	-	LG5:1342711:1342837:- 127(nt) - 58.50(kcal/mol)
	miR1511b	AACCGGCUCUGAUACCAAUUA	21	11504	19481	20532	4557	642	0	761.34	1506.9	1647.95	379.12	33.1	0	0.04	-	-	-	-	-	-	-	no location in *F.vesca* genome
	miR1511c	AACAGGCUCUGAUACCAAUUG	21	441	12415	12922	105	37	0	29.19	960.33	1037.15	8.74	1.91	0	0.07	-	-	-	-	-	-	-	no location in *F.vesca* genome
	miR1511d	CACCGGCUCUGAUACCAAUUG	21	827	12747	12338	178	47	0	54.73	986.01	990.28	14.81	2.42	0	0.04	-	-	-	-	-	-	-	no location in *F.vesca* genome
	miR1511e	GACCGGCUCUGAUACCAAUUG	21	2989	9731	10019	1327	391	0	197.81	752.71	804.15	110.4	20.16	0	0.1	-	-	-	-	-	-	-	no location in *F.vesca* genome
	miR1511f	AGCCGGCUCUGAUACCAAUUG	21	3496	7393	7437	1515	430	0	231.37	571.86	596.91	126.04	22.17	0	0.1	-	-	-	-	-	-	-	no location in *F.vesca* genome
	miR1511g	AACCGGCUCUGGUACCAAUUG	21	3387	6818	6815	1253	366	0	224.15	527.39	546.99	104.24	18.87	0	0.08	-	-	-	-	-	-	-	no location in *F.vesca* genome

	miR1511h	AACCGGCCCUGAUACCAAUUG	21	3056	6730	5882	965	345	0	202.25	520.58	472.1	80.28	17.79	0	0.09	-	-	-	-	-	-	-	no location in *F.vesca* genome
	miR1511i	AACCGGCUCUGAUACCAAUUU	21	6766	7076	7891	2349	1593	0	447.77	547.34	633.35	195.43	82.14	0	0.18	-	-	-	-	-	-	-	no location in *F.vesca* genome
	miR1511j	UACCGGCUCUGAUACCAAUUG	21	1634	5097	4500	769	61	0	108.14	394.26	361.18	63.98	3.15	0	0.03	-	-	-	-	-	-	-	no location in *F.vesca* genome
	miR1511k	AACCGGCUCUGAUACCGAUUG	21	2799	5298	5050	950	263	0	185.24	409.81	405.33	79.04	13.56	0	0.07	-	-	-	-	-	-	-	no location in *F.vesca* genome
	miR1511l	AACCGGCUCCGAUACCAAUUG	21	2678	5266	5080	909	255	0	177.23	407.34	407.73	75.63	13.15	0	0.07	-	-	-	-	-	-	-	no location in *F.vesca* genome
	miR1511m	AACCGGCUCUGAUACCAAUUC	21	3622	5781	5439	1047	386	0	239.7	447.17	436.55	87.11	19.9	0	0.08	-	-	-	-	-	-	-	no location in *F.vesca* genome
	miR1511n	AACCAGCUCUGAUACCAAUUG	21	3382	4380	4466	765	348	0	223.82	338.8	358.45	63.65	17.94	0	0.08	-	-	-	-	-	-	-	no location in *F.vesca* genome
	miR1511o	AACCGGCUCUGAUGCCAAUUG	21	2123	4059	4293	814	239	0	140.5	313.97	344.57	67.72	12.32	0	0.09	-	-	-	-	-	-	-	no location in *F.vesca* genome
	miR1511p	AACCGGCUCUGAUACCAGUUG	21	1798	4038	3934	682	182	0	118.99	312.35	315.75	56.74	9.38	0	0.08	-	-	-	-	-	-	-	no location in *F.vesca* genome
	miR1511q	AACCGGCUCUGACACCAAUUG	21	1837	3524	3321	590	186	0	121.57	272.59	266.55	49.09	9.59	0	0.08	-	-	-	-	-	-	-	no location in *F.vesca* genome
	miR1511r	AACUGGCUCUGAUACCAAUUG	21	1433	3388	3283	508	147	0	94.84	262.07	263.5	42.26	7.58	0	0.08	-	-	-	-	-	-	-	no location in *F.vesca* genome
	miR1511s	AACCGGCUCUGAUACCAAUCG	21	2012	4251	4190	575	187	0	133.15	328.82	336.3	47.84	9.64	0	0.07	-	-	-	-	-	-	-	no location in *F.vesca* genome
	miR1511t	AACGGGCUCUGAUACCAAUUG	21	384	2600	2784	106	39	0	25.41	201.12	223.45	8.82	2.01	0	0.08	-	-	-	-	-	-	-	no location in *F.vesca* genome
	miR1511u	AACCGGCUCUGAUACCAACUG	21	1234	2661	2436	454	72	0	81.67	205.83	195.52	37.77	3.71	0	0.05	-	-	-	-	-	-	-	no location in *F.vesca* genome
	miR1511v	AACCGGCUCUGAUACCCAUUG	21	681	2319	2134	161	37	0	45.07	179.38	171.28	13.39	1.91	0	0.04	-	-	-	-	-	-	-	no location in *F.vesca* genome
	miR1511w	AACCGACUCUGAUACCAAUUG	21	1053	2086	2064	352	137	0	69.69	161.36	165.66	29.29	7.06	0	0.1	-	-	-	-	-	-	-	no location in *F.vesca* genome

	miR1511x	AACCGGCUCUGAUACUAAUUG	21	1024	2031	1775	435	88	0	67.77	157.1	142.47	36.19	4.54	0	0.07	-	-	-	-	-	-	-	no location in *F.vesca* genome
	miR1511y	AAACGGCUCUGAUACCAAUUG	21	137	334	270	1888	111	0	9.07	25.84	21.67	157.07	5.72	0	0.63	-	-	-	-	-	-	-	no location in *F.vesca* genome
	miR1511z	AACCGGUUCUGAUACCAAUUG	21	1154	1852	1676	331	109	0	76.37	143.26	134.52	27.54	5.62	0	0.07	-	-	-	-	-	-	-	no location in *F.vesca* genome
	miR1511aa	AACCCGCUCUGAUACCAAUUG	21	1793	2293	2223	301	194	0	118.66	177.37	178.42	25.04	10	0	0.08	-	-	-	-	-	-	-	no location in *F.vesca* genome
	miR1511ab	AACCGGCUCUGAUAUCAAUUG	21	972	1599	1552	357	93	0	64.33	123.69	124.57	29.7	4.8	0	0.07	-	-	-	-	-	-	-	no location in *F.vesca* genome
	miR1511ac	AACCGGCUCUAAUACCAAUUG	21	710	1544	1407	174	26	0	46.99	119.43	112.93	14.48	1.34	0	0.03	-	-	-	-	-	-	-	no location in *F.vesca* genome
	miR1511ad	AACCGGCUCUGAUACCUAUUG	21	700	1486	1297	209	23	0	46.33	114.95	104.1	17.39	1.19	0	0.03	-	-	-	-	-	-	-	no location in *F.vesca* genome
	miR1511ae	AACCGGCACUGAUACCAAUUG	21	313	1378	1242	109	25	0	20.71	106.59	99.69	9.07	1.29	0	0.06	-	-	-	-	-	-	-	no location in *F.vesca* genome
	miR1511af	AAUCGGCUCUGAUACCAAUUG	21	705	1389	1315	296	95	0	46.66	107.44	105.55	24.63	4.9	0	0.11	-	-	-	-	-	-	-	no location in *F.vesca* genome
miR1513	miR1513	UUAAAUAUGUAAGAAGUCCUUG	22	0	0	0	0	0	3335	0	0	0	0	0	177.94	Both 0	-	-	-	-	-	-	-	no location in *F.vesca* genome
miR1861	miR1861	UCGAUCUUGUGAGCAGACUGU	21	0	0	0	0	7360	0	0	0	0	0	379.49	0	>> 1	-	-	-	-	-	-	-	no location in *F.vesca* genome
miR3437	miR3437	CCUGGACUUGUAUUUUUGUAC	21	5580	6672	3842	1765	194	0	369.28	516.09	308.37	146.84	10	0	0.03	-	-	-	-	-	-	-	no location in *F.vesca* genome
miR3449	miR3449	UGAUAGAACAAUGUAGAUA	19	0	0	0	1806	0	2714	0	0	0	150.25	0	144.81	Both 0	-	-	-	-	-	-	-	no location in *F.vesca* genome
miR3522	miR3522	UAGACCAAUUGACAGCUCUGU	21	0	0	0	5008	0	0	0	0	0	416.65	0	0	Both 0	-	-	-	-	-	-	-	no location in *F.vesca* genome
miR3633	miR3633	UCCCUAUUCCACCUAUUCCCCA	22	1215	2017	2048	288	0	0	80.41	156.02	164.38	23.96	0	0	0	-	-	-	-	-	-	-	LG4:24750978:24751080:- 103(nt) -46.60(kcal/mol)

miR3708	miR3708	UCACAAAGAUGUCGUCGUAUA	21	0	0	0	1515	0	0	0	0	0	126.04	0	0	Both 0	-	-	-	-	-	-	-	no location in *F.vesca* genome
miR4380	miR4380	CGAUUGUUGAUCCGAAUGUUGAUC	24	0	1846	0	0	0	0	0	142.79	0	0	0	0	Both 0	-	-	-	-	-	-	-	no location in *F.vesca* genome
miR4398	miR4398	UCUCAGCGGAGGAGAAAGGAC	21	0	0	0	1468	0	0	0	0	0	122.13	0	0	Both 0	-	-	-	-	-	-	-	no location in *F.vesca* genome
miR4414	miR4414	UCGCAAGGAUGCGGAGCGUGA	21	0	0	0	0	2835	0	0	0	0	0	146.18	0	>> 1	-	-	-	-	-	-	-	no location in *F.vesca* genome
miR5213	miR5213	UGCGAGUGUCUUCACCUCUGAA	22	6672	11410	8441	3284	627	0	441.55	882.59	677.5	273.22	32.33	0	0.07	-	-	-	-	-	-	-	LG0:1732175:1732273:+ 99(nt) -45.92(kcal/mol)
miR5258	miR5258	UCAAGCUGACAAAGAAGACUG	21	0	0	0	2082	0	0	0	0	0	173.21	0	0	Both 0	-	-	-	-	-	-	-	no location in *F.vesca* genome
miR5260	miR5260	UUUGACUGUUGCUCAUGGCCU	21	0	0	0	9956	0	1225	0	0	0	828.3	0	65.36	Both 0	-	-	-	-	-	-	-	no location in *F.vesca* genome
miR5289	miR5289a	CGGAAAACUGAAAUUCGGCGG	21	1789	1966	3100	1830	0	0	118.4	152.07	248.81	152.25	0	0	0	-	-	-	-	-	-	-	no location in *F.vesca* genome
	miR5289b	CGGAAAACUGGAAUUCGGCGG	21	2687	2587	2388	1915	0	0	177.83	200.11	191.67	159.32	0	0	0	-	-	-	-	-	-	-	no location in *F.vesca* genome
miR5290	miR5290a	UUGGAGAGAGAGUAGACAAUG	21	21736	60200	75632	7838	4658	0	1438.49	4656.6	6070.42	652.09	240.17	0	0.17	-	-	-	-	-	-	-	LG6:12165629:12165718:+ 90(nt) -61.00(kcal/mol)
	miR5290b	UUGGAGAGAGAGUAGACAUUG	21	218	1027	1515	37	0	0	14.43	79.44	121.6	3.08	0	0	0	-	-	-	-	-	-	-	no location in *F.vesca* genome
	miR5290c	UUGGAGAGAGAGUAGACAAUA	21	323	713	1249	122	19	0	21.38	55.15	100.25	10.15	0.98	0	0.05	-	-	-	-	-	-	-	no location in *F.vesca* genome
miR5642	miR5642	UCUCGCAGCUUGUAGGUGCU	20	0	0	0	1243	0	0	0	0	0	103.41	0	0	Both 0	-	-	-	-	-	-	-	no location in *F.vesca* genome
miR5643	miR5643	UGGCUCUUUAAGAUCGGCUGG	21	0	0	0	2855	0	0	0	0	0	237.53	0	0	Both 0	-	-	-	-	-	-	-	no location in *F.vesca* genome
miR5656	miR5656	UCGAAGUGGAGAUUGUGUUUU	21	0	0	0	6027	0	0	0	0	0	501.42	0	0	Both 0	-	-	-	-	-	-	-	no location in *F.vesca* genome

miR5663	miR5663	UGAGAAAAUGCAACUCUUAGCG	22	0	0	0	1243	0	0	0	0	0	103.41	0	0	Both 0	-	-	-	-	-	-	-	no location in *F.vesca* genome
miR5813	miR5813	AAGACAGCAGGACGGUGGUCAUG	23	445	976	1953	417	125	0	29.45	75.5	156.75	34.69	6.45	0	0.22	-	-	-	-	-	-	-	LG6:19667808:19668129:- 322(nt) -116.50(kcal/mol)
miR6103	miR6103	UUGUUCAGACAACCCUGGGAAC	22	0	0	0	1554	0	0	0	0	0	129.29	0	0	Both 0	-	-	-	-	-	-	-	no location in *F.vesca* genome
miR6437	miR6437	UCACGGACGGUAGGCUUGAAGC	22	0	0	0	2637	0	0	0	0	0	219.39	0	0	Both 0	-	-	-	-	-	-	-	no location in *F.vesca* genome
miR7484	miR7484	UAGAGUCCGAAUUGUAAUUUGUA	23	0	105	92	1289	1922	11440	0	8.12	7.38	107.24	99.1	610.38	>> 1	-	-	-	-	-	-	-	no location in *F.vesca* genome
miR7708	miR7708	UGUAAUGACUGCACAAGACUGC	22	706	1349	328	389	349	0	46.72	104.35	26.33	32.36	17.99	0	0.39	-	-	-	-	-	-	-	no location in *F.vesca* genome
miR7732	miR7732	UCGAGAUCUUGGAGGGGACCC	21	0	0	0	0	2766	0	0	0	0	0	142.62	0	>> 1	-	-	-	-	-	-	-	no location in *F.vesca* genome
miR7767	miR7767	CCAAGAUGAGUGCUCUCCU	19	826	2702	5262	398	558	0	54.66	209.01	422.34	33.11	28.77	0	0.53	-	-	-	-	-	-	-	no location in *F.vesca* genome
miR7782	miR7782a	ACCUAGCUCUGAUACCAUGUG	21	456224	958281	1152248	174672	47515	25	30192.92	74124.99	92482.41	14532.05	2449.94	1.33	0.08	-	-	-	-	-	-	-	LG5:1343333:1343431:- 99(nt) -40.80(kcal/mol)
	miR7782b	ACCUAGCUCUGAUACCAUGUA	21	102411	23403	31163	3791	611	0	677.75	1810.27	2501.22	315.4	31.5	0	0.05	-	-	-	-	-	-	-	no location in *F.vesca* genome
	miR7782c	ACCCAGCUCUGAUACCAUGUG	21	1539	8316	12863	422	82	0	101.85	643.26	1032.42	35.11	4.23	0	0.04	-	-	-	-	-	-	-	no location in *F.vesca* genome
	miR7782d	CCCUAGCUCUGAUACCAUGUG	21	379	8797	9779	62	18	0	25.08	680.47	784.89	5.16	0.93	0	0.04	-	-	-	-	-	-	-	no location in *F.vesca* genome
	miR7782e	ACCUAGCUCUGAUACCAUGUC	21	4812	8817	10659	1673	251	0	318.46	682.01	855.52	139.19	12.94	0	0.04	-	-	-	-	-	-	-	no location in *F.vesca* genome
	miR7782f	ACCUAGCUCUGAUACCAUGUU	21	3501	5551	9757	1548	485	0	231.7	429.38	783.12	128.79	25.01	0	0.11	-	-	-	-	-	-	-	no location in *F.vesca* genome
	miR7782g	GCCUAGCUCUGAUACCAUGUG	21	1695	7327	8029	600	124	0	112.18	566.76	644.43	49.92	6.39	0	0.06	-	-	-	-	-	-	-	no location in *F.vesca* genome

	miR7782h	ACCUAGCUCUGAUACCAUGCG	21	2416	5701	7540	930	215	0	159.89	440.98	605.18	77.37	11.09	0	0.07	-	-	-	-	-	-	-	no location in *F.vesca* genome
	miR7782i	ACCUAGCUCUGGUACCAUGUG	21	1380	3709	4209	574	94	0	91.33	286.9	337.83	47.75	4.85	0	0.05	-	-	-	-	-	-	-	no location in *F.vesca* genome
	miR7782j	ACCAAGCUCUGAUACCAUGUG	21	366	2573	4166	84	15	0	24.22	199.03	334.37	6.99	0.77	0	0.03	-	-	-	-	-	-	-	no location in *F.vesca* genome
	miR7782k	UCCUAGCUCUGAUACCAUGUG	21	670	4020	4087	274	27	0	44.34	310.96	328.03	22.8	1.39	0	0.03	-	-	-	-	-	-	-	no location in *F.vesca* genome
	miR7782l	ACCUAGCUCUGAUACCACGUG	21	1565	3768	4358	512	87	0	103.57	291.46	349.78	42.6	4.49	0	0.04	-	-	-	-	-	-	-	no location in *F.vesca* genome
	miR7782m	ACCUAGCCCUGAUACCAUGUG	21	1643	3260	4065	526	111	0	108.73	252.17	326.27	43.76	5.72	0	0.05	-	-	-	-	-	-	-	no location in *F.vesca* genome
	miR7782n	ACCUAGCUCCGAUACCAUGUG	21	1504	3114	3798	498	70	0	99.53	240.87	304.84	41.43	3.61	0	0.04	-	-	-	-	-	-	-	no location in *F.vesca* genome
	miR7782o	ACCUAGCUCUGAUACCGUGUG	21	1331	3173	3608	464	83	0	88.09	245.44	289.59	38.6	4.28	0	0.05	-	-	-	-	-	-	-	no location in *F.vesca* genome
	miR7782p	ACCUAGCUCUGAUGCCAUGUG	21	951	2422	2884	389	65	0	62.94	187.35	231.48	32.36	3.35	0	0.05	-	-	-	-	-	-	-	no location in *F.vesca* genome
	miR7782q	ACCUGGCUCUGAUACCAUGUG	21	1208	2264	2785	410	72	0	79.95	175.13	223.53	34.11	3.71	0	0.05	-	-	-	-	-	-	-	no location in *F.vesca* genome
	miR7782r	ACCUAGCUCUGACACCAUGUG	21	817	2028	2394	268	59	0	54.07	156.87	192.15	22.3	3.04	0	0.06	-	-	-	-	-	-	-	no location in *F.vesca* genome
	miR7782s	ACCUAGCUCUGAUACCAUGGG	21	1238	2052	2586	422	63	0	81.93	158.73	207.56	35.11	3.25	0	0.04	-	-	-	-	-	-	-	no location in *F.vesca* genome
	miR7782t	ACCUAGCUCUGAUACCAUAUGU	22	426	1138	1400	1944	27	0	28.19	88.03	112.37	161.73	1.39	0	0.05	-	-	-	-	-	-	-	no location in *F.vesca* genome
	miR7782u	AUCUAGCUCUGAUACCAUGUG	21	663	1311	1517	309	61	0	43.88	101.41	121.76	25.71	3.15	0	0.07	-	-	-	-	-	-	-	no location in *F.vesca* genome
	miR7782v	ACCUAGCUCUGAUACUAUGUG	21	454	1215	1259	817	99	0	30.05	93.98	101.05	67.97	5.1	0	0.17	-	-	-	-	-	-	-	no location in *F.vesca* genome
	miR7782w	ACCUAACUCUGAUACCAUGUG	21	406	1049	1288	139	28	0	26.87	81.14	103.38	11.56	1.44	0	0.05	-	-	-	-	-	-	-	no location in *F.vesca* genome

miR7984	miR7984	UCCGACUUUGUGAAAUGACUU	21	6662	13026	14024	4851	2668	0	440.89	1007.59	1125.6	403.58	137.57	0	0.31	-	-	-	-	-	-	-	no location in *F.vesca* genome
miR8033	miR8033	UCAAAUCUGACUUUAGGGGC	20	0	0	0	7073	0	3024	0	0	0	588.45	0	161.35	Both 0	-	-	-	-	-	-	-	no location in *F.vesca* genome
miR8122	miR8122	UUCCGACAAGACUUUUCUCUU	21	0	0	0	0	2937	0	0	0	0	0	151.44	0	>> 1	-	-	-	-	-	-	-	no location in *F.vesca* genome
miR8175	miR8175	UUCCCGGCAAUGGAACCA	18	143	717	1284	1838	808	0	9.46	55.46	103.06	152.91	41.66	0	4.4	-	-	-	-	-	-	-	no location in *F.vesca* genome
miR8183	miR8183	UUUCAGUUCGAAGGAUUGUG	20	0	0	0	3775	208	524	0	0	0	314.07	10.72	27.96	>> 1	-	-	-	-	-	-	-	no location in *F.vesca* genome
miR8581	miR8581	UGGAAAUUCUUGUAUGCACGACGU	24	2250	1503	0	0	0	0	148.91	116.26	0	0	0	0	0	-	-	-	-	-	-	-	LG3:4764161:4764339:- 179(nt) -27.50(kcal/mol)
miR8666	miR8666	UUUGGUGAUAUAGACGUAAAU	21	0	0	0	4065	0	0	0	0	0	338.19	0	0	Both 0	-	-	-	-	-	-	-	no location in *F.vesca* genome
miR8781	miR8781	UUUGAUUGUGAAGUUUGACGGAGA	24	1734	0	0	0	0	0	114.76	0	0	0	0	0	0	-	-	-	-	-	-	-	LG1:4166047:4166293:+ 247(nt) -53.20(kcal/mol)
miR9472	miR9472	UUCCAAUCUCUGAUACAAUG	20	0	0	0	0	4480	0	0	0	0	0	230.99	0	>> 1	-	-	-	-	-	-	-	no location in *F.vesca* genome
miR9653	miR9653	ACCAAGAUCUCUGAGGUCU	19	0	1703	2576	0	0	0	0	131.73	206.76	0	0	0	Both 0	-	-	-	-	-	-	-	no location in *F.vesca* genome
miR9767	miR9767	UGAAAAGGACUUUGAAAAAAG	21	0	0	3088	0	0	0	0	0	247.85	0	0	0	Both 0	-	-	-	-	-	-	-	no location in *F.vesca* genome

Note： T1, T2, T3, and T4 indicated that after inoculation, red ripening fruits were chose and took at 48 h, 72 h, 96 h and 120 h, respectively. CK1 indicated that red ripening fruits were took from strawberry plants treated with sterile water. CK2 indicates *B. cinerea*. The abbreviations represent: fan, *Fragaria ananassa*; ath, *Arabidopsis thaliana*; osa, *Oryza sativa*; ptc, *Populus trichocarpa*; vvi, *Vitis vinifera*; ctr, *Citrus trifoliata*; csi, *Citrus sinensi*; mdm, *Malus domestic*. The plus symbols indicate: ++, miRNA sequences of strawberry were identical exactly to those in other species; +, miRNA sequences of strawberry were highly conserved in other species but have four or fewer variations in some nucleotide positions.

Table S2 The ratios of the 51 most abundant conserved miRNAs between the infected libraries and CK1

Conserved miRNA Name	Ratio			
	T1/CK1	T2/CK1	T3/CK1	T4/CK1
miR156	3.13	1.68	1.83	0.39
miR157	2.66	2.52	2.15	0.51
miR159	3.36	4.532	0.49	0.14
miR162	1.04	1.08	0.04	0.02
miR164	6.57	7.72	0.54	0.18
miR166	2.86	3.5	1.11	0.2
miR167	2.21	2.7	0.18	0.02
miR319	3.14	2.21	0.76	0.17
miR395	0.43	0.85	0.1	0.06
miR396	1.54	1.66	0.24	0.09
miR398	3.03	0.81	0	0.06
miR399	1.66	2.84	0.08	0.05
miR408	2.42	2.56	0.35	0.21
miR477	2.42	1.75	0.39	0.27
miR482	2.89	4.05	0.17	0.02
miR1026	>> 1	>> 1	Both 0	Both 0
miR1511	2.13	2.19	0.48	0.11
miR1861	Both 0	Both 0	Both 0	>> 1
miR3437	1.4	0.84	0.4	0.03
miR3522	Both 0	Both 0	>> 1	Both 0
miR3633	1.94	2.04	0.3	>> 1
miR3708	Both 0	Both 0	>> 1	Both 0
miR4380	>> 1	Both 0	Both 0	Both 0
miR4398	Both 0	Both 0	>> 1	Both 0
miR4414	Both 0	Both 0	Both 0	>> 1
miR5213	2	1.53	0.62	0.07
miR5258	Both 0	Both 0	>> 1	Both 0
miR5260	Both 0	Both 0	>> 1	Both 0
miR5289	1.19	1.49	1.05	>> 1
miR5290	3.25	4.27	0.45	0.16
miR5642	Both 0	Both 0	>> 1	Both 0
miR5643	Both 0	Both 0	>> 1	Both 0
miR5656	Both 0	Both 0	>> 1	Both 0
miR5663	Both 0	Both 0	>> 1	Both 0
miR5813	2.56	5.32	1.18	0.22
miR6103	Both 0	Both 0	>> 1	Both 0
miR6437	Both 0	Both 0	>> 1	Both 0
miR7708	2.23	0.56	0.7	0.39
miR7732	Both 0	Both 0	Both 0	>> 1
miR7767	3.82	7.73	0.61	0.53
miR7782	2.51	3.15	0.49	0.08

miR7984	2.29	2.55	0.92	0.31
miR8122	Both 0	Both 0	Both 0	>> 1
miR8175	5.86	10.9	16.16	4.4
miR8183	Both 0	Both 0	>> 1	>> 1
miR8581	0.78	0	0	>> 1
miR8666	Both 0	Both 0	>> 1	Both 0
miR8781	0	0	0	>> 1
miR9472	Both 0	Both 0	Both 0	>> 1
miR9653	>> 1	>> 1	Both 0	Both 0
miR9767	Both 0	>> 1	Both 0	Both 0

Table S3 The ratios of 35 novel miRNAs between infected libraries and CK1

Novel miRNA Name	Ratio			
	T1/CK1	T2/CK1	T3/CK1	T4/CK1
fan-novel-001	2.49	1.53	0	0.04
fan-novel-002	Both 0	Both 0	>> 1	Both 0
fan-novel-003	3.3	1	0.8	0.67
fan-novel-004	4.08	5.18	0.66	0
fan-novel-005	4.05	3.83	0	0
fan-novel-006	4.56	1.5	0.43	0
fan-novel-007	0	0	0	0.13
	0.86	0.8	0.7	0.13
	0	0.79	0	0.13
	0	0.8	0	0.13
	0	0	0	0.13
	0	0.8	0	0.13
	0.86	0.8	0.69	0.13
	0	0.83	0	0.14
	0.86	0.8	0.69	0.13
	0.86	0.8	0.69	0.13
	0.88	0.84	0	0
	Both 0	Both 0	>> 1	>> 1
	Both 0	>> 1	Both 0	>> 1
	0	0.8	0	0.13
	0	0.8	0	0.13
fan-novel-008	Both 0	Both 0	Both 0	>> 1
fan-novel-009	1.7	0.7	0	0
	1.7	0.7	0	0
fan-novel-010	2.05	1.16	0.33	0
	2.05	1.16	0.33	0
fan-novel-011	Both 0	Both 0	>> 1	Both 0
fan-novel-012	>> 1	Both 0	Both 0	>> 1

fan-novel-013	Both 0	>> 1	Both 0	Both 0
fan-novel-014	Both 0	>> 1	>> 1	>> 1
	3.62	0	0	0
fan-novel-015	1.8	2	0.24	0.04
fan-novel-016	>> 1	Both 0	Both 0	Both 0
	>> 1	Both 0	Both 0	Both 0
fan-novel-017	Both 0	>> 1	Both 0	Both 0
fan-novel-018	2.16	1	1.19	0
fan-novel-019	2.52	2.43	0.17	0
fan-novel-020	3.19	2.67	0.26	0
fan-novel-021	Both 0	>> 1	Both 0	Both 0
fan-novel-022	1.76	1.83	0	0
	Both 0	Both 0	>> 1	Both 0
fan-novel-023	>> 1	Both 0	Both 0	>> 1
	0	2.11	1.75	0
fan-novel-024	2.73	1.17	0.3	0.03
fan-novel-025	2.51	0.93	0.24	0.22
fan-novel-026	2	1.42	0.52	0
fan-novel-027	2.32	2.32	0.62	0.26
fan-novel-028	2.97	3.22	0	0
fan-novel-029	2.57	1.15	0.66	0.1
fan-novel-030	1.6	2.41	0.5	0.25
fan-novel-031	3.9	2.8	0.63	0
fan-novel-032	3.32	2.53	0	0.16
fan-novel-033	Both 0	Both 0	>> 1	>> 1
fan-novel-034	0	0	0.42	0.15
fan-novel-035	>> 1	>> 1	Both 0	Both 0

Table S4: Putative conserved miRNA target genes in infected strawberry fruits

Name	Target gene No.	Target gene description	Possible function	E value
miR156	gi|470113815	PREDICTED: pentatricopeptide repeat-containing protein At2g22070-like [Fragaria vesca subsp. vesca]	Null	0
	gi|470122372	PREDICTED: squamosa promoter-binding-like protein 6-like [Fragaria vesca subsp. vesca]	Null	0
	gi|470123009	PREDICTED: squamosa promoter-binding-like protein 13A-like [Fragaria vesca subsp. vesca]	Null	0
	gi|470137245	PREDICTED: squamosa promoter-binding-like protein 17-like [Fragaria vesca subsp. vesca]	Null	0
	gi|470137555	PREDICTED: squamosa promoter-binding-like protein 13A-like [Fragaria vesca subsp. vesca]	Null	0
	gi|470138782	PREDICTED: putative ribonuclease H protein At1g65750-like [Fragaria vesca subsp. vesca]	Null	1.02E-153
	gi|470145723	PREDICTED: squamosa promoter-binding-like protein 7-like [Fragaria vesca subsp. vesca]	Null	6.24E-77
	gi|470149113	PREDICTED: squamosa promoter-binding-like protein 12-like [Fragaria vesca subsp. vesca]	Null	0
	gi|470149134	PREDICTED: uncharacterized protein LOC101314819 [Fragaria vesca subsp. vesca]	Null	1.18E-158
miR157	gi|470122372	PREDICTED: squamosa promoter-binding-like protein 6-like [Fragaria vesca subsp. vesca]	Null	0
	gi|470123009	PREDICTED: squamosa promoter-binding-like protein 13A-like [Fragaria vesca subsp. vesca]	Null	0
	gi|470137245	PREDICTED: squamosa promoter-binding-like protein 17-like [Fragaria vesca subsp. vesca]	Null	0
	gi|470137555	PREDICTED: squamosa promoter-binding-like protein 13A-like [Fragaria vesca subsp. vesca]	Null	0
	gi|470145723	PREDICTED: squamosa promoter-binding-like protein 7-like [Fragaria vesca subsp. vesca]	Null	6.24E-77
	gi|470149113	PREDICTED: squamosa promoter-binding-like protein 12-like [Fragaria vesca subsp. vesca]	Null	0
miR159	gi|462407492	hypothetical protein PRUPE_ppa005691mg [Prunus persica]	Null	2.34E-116
	gi|470103239	PREDICTED: patatin group A-3-like [Fragaria vesca subsp. vesca]	catalytic activity	0
	gi|470111534	PREDICTED: uncharacterized protein LOC101312896 [Fragaria vesca subsp. vesca]	Null	3.32E-107
	gi|470120371	PREDICTED: uncharacterized protein LOC101301378 [Fragaria vesca subsp. vesca]	Null	0
	gi|470141497	PREDICTED: transcription factor GAMYB-like [Fragaria vesca subsp. vesca]	Null	0
miR162	no target in F.vesca			
miR164	gi|462414719	hypothetical protein PRUPE_ppa008154mg [Prunus persica]	binding	0
	gi|470108395	PREDICTED: protein SET DOMAIN GROUP 41-like [Fragaria vesca subsp. vesca]	Null	0

	gi|470110456	PREDICTED: protein CUP-SHAPED COTYLEDON 2-like [Fragaria vesca subsp. vesca]	Null	0
	gi|470116068	PREDICTED: NAC domain-containing protein 21/22-like [Fragaria vesca subsp. vesca]	multicellular organismal process	0
	gi|470126738	PREDICTED: NAC domain-containing protein 100-like isoform 2 [Fragaria vesca subsp. vesca]	nucleic acid binding transcription factor activity	0
	gi|470137039	PREDICTED: NAC domain-containing protein 100-like [Fragaria vesca subsp. vesca]	Null	1.62E-177
	gi|470138483	PREDICTED: protein CUP-SHAPED COTYLEDON 2-like [Fragaria vesca subsp. vesca]	Null	3.16E-169
	gi|470143742	PREDICTED: UDP-glucuronic acid decarboxylase 1-like [Fragaria vesca subsp. vesca]	single-organism process	5.57E-41
miR166	gi|470109778	PREDICTED: homeobox-leucine zipper protein ATHB-8-like [Fragaria vesca subsp. vesca]	cell	0
	gi|470110617	PREDICTED: homeobox-leucine zipper protein REVOLUTA-like [Fragaria vesca subsp. vesca]	cell	0
	gi|470115610	PREDICTED: homeobox-leucine zipper protein HOX32-like [Fragaria vesca subsp. vesca]	reproductive process	0
	gi|470134042	PREDICTED: homeobox-leucine zipper protein ATHB-15-like [Fragaria vesca subsp. vesca]	cell	0
miR167	gi|470104378	PREDICTED: auxin response factor 8-like isoform 1 [Fragaria vesca subsp. vesca]	cell	0
	gi|470109879	PREDICTED: flavin-containing monooxygenase YUCCA6-like [Fragaria vesca subsp. vesca]	binding	0
	gi|470124343	PREDICTED: mediator of RNA polymerase II transcription subunit 33A-like [Fragaria vesca subsp. vesca]	Null	0
	gi|470132859	PREDICTED: AP-3 complex subunit mu-1-like [Fragaria vesca subsp. vesca]	transporter activity	0
miR319	gi|470115874	PREDICTED: transcription factor TCP4-like [Fragaria vesca subsp. vesca]	cellular process	0
	gi|470134668	PREDICTED: transcription factor TCP4-like [Fragaria vesca subsp. vesca]	Null	8.46E-175
	gi|470141497	PREDICTED: transcription factor GAMYB-like [Fragaria vesca subsp. vesca]	Null	0
miR395	gi|470119657	PREDICTED: uncharacterized protein LOC101293764 [Fragaria vesca subsp. vesca]	Null	0
	gi|470119659	PREDICTED: uncharacterized protein LOC101294054 [Fragaria vesca subsp. vesca]	catalytic activity	0
	gi|470121039	PREDICTED: ATP sulfurylase 1, chloroplastic-like [Fragaria vesca subsp. vesca]	catalytic activity	0
	gi|470138171	PREDICTED: sulfate transporter 2.1-like [Fragaria vesca subsp. vesca]	transporter activity	0
miR396	gi|462408375	hypothetical protein PRUPE_ppa017820mg, partial [Prunus persica]	Null	2.84E-32

	gi|462417055	hypothetical protein PRUPE_ppa000616mg [Prunus persica]	Null	0
	gi|470103015	PREDICTED: 50S ribosomal protein L6, chloroplastic-like [Fragaria vesca subsp. vesca]	structural molecule activity	1.05E-95
	gi|470103122	PREDICTED: probable cadmium/zinc-transporting ATPase HMA1, chloroplastic-like [Fragaria vesca subsp. vesca]	cellular process	0
	gi|470103469	PREDICTED: uncharacterized protein LOC101308800 [Fragaria vesca subsp. vesca]	Null	3.30E-27
	gi|470106672	PREDICTED: E3 ubiquitin-protein ligase RMA3-like isoform 1 [Fragaria vesca subsp. vesca]	Null	9.85E-135
	gi|470112243	PREDICTED: uncharacterized protein LOC101299265 [Fragaria vesca subsp. vesca]	Null	2.76E-66
	gi|470118296	PREDICTED: F-box/kelch-repeat protein At3g06240-like [Fragaria vesca subsp. vesca]	Null	1.55E-70
	gi|470135722	PREDICTED: putative phospholipid-transporting ATPase 4-like [Fragaria vesca subsp. vesca]	binding	0
	gi|470136387	PREDICTED: sulfite oxidase-like [Fragaria vesca subsp. vesca]	cell	7.06E-107
	gi|470138838	PREDICTED: uncharacterized protein LOC101300171 [Fragaria vesca subsp. vesca]	Null	5.95E-39
	gi|470146177	PREDICTED: pentatricopeptide repeat-containing protein At3g16010-like [Fragaria vesca subsp. vesca]	Null	1.10E-30
	gi|470147065	PREDICTED: uncharacterized protein LOC101291584 [Fragaria vesca subsp. vesca]	biological regulation	5.24E-144
miR398	no target in F.vesca			
miR399	gi|470107193	PREDICTED: probable inorganic phosphate transporter 1-7-like [Fragaria vesca subsp. vesca]	transporter activity	0
	gi|470111967	PREDICTED: inorganic phosphate transporter 1-4-like [Fragaria vesca subsp. vesca]	localization	0
	gi|470123620	PREDICTED: probable inorganic phosphate transporter 1-7-like [Fragaria vesca subsp. vesca]	localization	0
	gi|470146771	PREDICTED: inorganic phosphate transporter 1-1-like [Fragaria vesca subsp. vesca]	transporter activity	0
miR408	gi|470125412	PREDICTED: SNARE-interacting protein KEULE-like [Fragaria vesca subsp. vesca]	membrane	4.95E-88
miR477	gi|470117875	PREDICTED: huntingtin-interacting protein K-like [Fragaria vesca subsp. vesca]	Null	8.73E-55
	gi|470146242	PREDICTED: probable replication factor C subunit 3-like [Fragaria vesca subsp. vesca]	binding	0
	gi|470148493	PREDICTED: DELLA protein GAI1-like [Fragaria vesca subsp. vesca]	Null	0
	gi|470149296	PREDICTED: DELLA protein GAI1-like [Fragaria vesca subsp. vesca]	Null	0
miR482	gi|470105594	PREDICTED: uncharacterized protein LOC101298839 [Fragaria vesca subsp. vesca]	Null	6.43E-24
	gi|470107037	PREDICTED: uncharacterized protein LOC101309407 [Fragaria vesca subsp. vesca]	Null	1.43E-92

	gi|470133027	PREDICTED: uncharacterized protein LOC101304885 [Fragaria vesca subsp. vesca]	Null	0
	gi|470138241	PREDICTED: cyclic phosphodiesterase-like [Fragaria vesca subsp. vesca]	cell	2.31E-107
	gi|470146236	PREDICTED: DNA repair helicase XPB2-like [Fragaria vesca subsp. vesca]	Null	3.77E-06
miR1026	no target in F.vesca			
miR1511	gi|470111852	PREDICTED: heat shock cognate 70 kDa protein-like [Fragaria vesca subsp. vesca]	response to stimulus	0
miR1861	no target in F.vesca			
miR3437	gi|470118310	PREDICTED: paired amphipathic helix protein Sin3-like 1-like [Fragaria vesca subsp. vesca]	Null	3.99E-37
	gi|470148599	PREDICTED: uncharacterized protein LOC101295192 [Fragaria vesca subsp. vesca]	Null	0
	gi|470149050	PREDICTED: geraniol 8-hydroxylase-like [Fragaria vesca subsp. vesca]	Null	2.69E-57
miR3522	no target in F.vesca			
miR3633	gi|470105114	PREDICTED: DNA ligase 1-like [Fragaria vesca subsp. vesca]	response to stimulus	2.05E-47
	gi|470109667	PREDICTED: UDP-glucuronate:xylan alpha-glucuronosyltransferase 2-like [Fragaria vesca subsp. vesca]	cell	0
miR3708	gi|470111765	PREDICTED: uncharacterized protein LOC101308638 [Fragaria vesca subsp. vesca]	Null	0
miR4380	no target in F.vesca			
miR4398	gi|470117825	PREDICTED: uncharacterized protein LOC101297816 [Fragaria vesca subsp. vesca]	binding	0
	gi|470131108	PREDICTED: putative ribonuclease H protein At1g65750-like [Fragaria vesca subsp. vesca]	Null	2.23E-65
miR4414	gi|470134006	PREDICTED: uncharacterized protein LOC101301403 [Fragaria vesca subsp. vesca]	Null	1.58E-39
miR5213	gi|462421411	hypothetical protein PRUPE_ppa022336mg [Prunus persica]	binding	8.46E-60
	gi|470104981	PREDICTED: TMV resistance protein N-like [Fragaria vesca subsp. vesca]	response to stimulus	0
	gi|470105935	PREDICTED: TMV resistance protein N-like [Fragaria vesca subsp. vesca]	Null	0
	gi|470105945	PREDICTED: protein SUPPRESSOR OF npr1-1, CONSTITUTIVE 1-like [Fragaria vesca subsp. vesca]	Null	1.29E-51
	gi|470116781	PREDICTED: uncharacterized protein LOC101310969 [Fragaria vesca subsp. vesca]	Null	5.54E-88
	gi|470117728	PREDICTED: TMV resistance protein N-like [Fragaria vesca subsp. vesca]	Null	1.92E-76
	gi|470118016	PREDICTED: uncharacterized protein LOC101302247 [Fragaria vesca subsp. vesca]	catalytic activity	0
	gi|470119624	PREDICTED: TMV resistance protein N-like [Fragaria vesca subsp. vesca]	Null	0
	gi|470119639	PREDICTED: disease resistance protein At4g27190-like [Fragaria vesca subsp. vesca]	Null	8.93E-58

	gi|470119641	PREDICTED: TMV resistance protein N-like [Fragaria vesca subsp. vesca]	Null	6.86E-103
	gi|470119655	PREDICTED: protein SUPPRESSOR OF npr1-1, CONSTITUTIVE 1-like [Fragaria vesca subsp. vesca]	Null	4.68E-85
	gi|470120199	PREDICTED: TMV resistance protein N-like [Fragaria vesca subsp. vesca]	binding	7.27E-87
	gi|470120447	PREDICTED: disease resistance protein RPS2-like [Fragaria vesca subsp. vesca]	Null	2.35E-122
	gi|470120449	PREDICTED: formin-like protein 20-like [Fragaria vesca subsp. vesca]	binding	0
	gi|470120526	PREDICTED: TMV resistance protein N-like [Fragaria vesca subsp. vesca]	Null	1.17E-87
	gi|470125210	PREDICTED: TMV resistance protein N-like [Fragaria vesca subsp. vesca]	catalytic activity	0
	gi|470130300	PREDICTED: TMV resistance protein N-like [Fragaria vesca subsp. vesca]	Null	3.96E-75
	gi|470131156	PREDICTED: TMV resistance protein N-like [Fragaria vesca subsp. vesca]	Null	0
	gi|470131186	PREDICTED: TMV resistance protein N-like [Fragaria vesca subsp. vesca]	binding	3.06E-93
	gi|470131383	PREDICTED: disease resistance protein At4g27190-like [Fragaria vesca subsp. vesca]	Null	0
	gi|470138421	PREDICTED: TMV resistance protein N-like [Fragaria vesca subsp. vesca]	response to stimulus	1.02E-28
	gi|470138529	PREDICTED: TMV resistance protein N-like [Fragaria vesca subsp. vesca]	Null	1.87E-108
	gi|470144488	PREDICTED: TMV resistance protein N-like [Fragaria vesca subsp. vesca]	Null	0
	gi|470145292	PREDICTED: uncharacterized protein LOC101307637 [Fragaria vesca subsp. vesca]	Null	0
	gi|470145730	PREDICTED: uncharacterized protein LOC101311138 [Fragaria vesca subsp. vesca]	Null	0
	gi|470145781	PREDICTED: TMV resistance protein N-like [Fragaria vesca subsp. vesca]	membrane part	6.07E-63
	gi|470146189	PREDICTED: TMV resistance protein N-like [Fragaria vesca subsp. vesca]	Null	4.40E-90
	gi|470146761	PREDICTED: TMV resistance protein N-like [Fragaria vesca subsp. vesca]	Null	0
	gi|470146936	PREDICTED: uncharacterized protein LOC101294615 [Fragaria vesca subsp. vesca]	cellular process	4.06E-116
	gi|470147294	PREDICTED: protein SUPPRESSOR OF npr1-1, CONSTITUTIVE 1-like [Fragaria vesca subsp. vesca]	Null	0
	gi|470147298	PREDICTED: TMV resistance protein N-like [Fragaria vesca subsp. vesca]	Null	1.14E-175
miR5258	gi|470109018	PREDICTED: uncharacterized protein LOC101309412 [Fragaria vesca subsp. vesca]	organelle part	0
	gi|470129065	PREDICTED: probable glutamyl endopeptidase, chloroplastic-like [Fragaria vesca subsp. vesca]	catalytic activity	0
miR5260	gi|21672041	Putative retroelement [Oryza sativa Japonica Group]	Null	1.81E-24
	gi|62701853	retrotransposon protein, putative, unclassified [Oryza sativa Japonica Group]	Null	9.91E-31

miR5289	gi|147804870	hypothetical protein VITISV_005133 [Vitis vinifera]	Null	8.61E-09
	gi|147820415	hypothetical protein VITISV_008274 [Vitis vinifera]	Null	3.90E-16
	gi|156763844	putative reductase [Nicotiana tabacum]	cellular process	2.27E-90
	gi|225437408	PREDICTED: uncharacterized protein LOC100246950 [Vitis vinifera]	Null	5.24E-19
	gi|225443998	PREDICTED: ABC transporter C family member 2-like [Vitis vinifera]	catalytic activity	0
	gi|255578422	monoxygenase, putative [Ricinus communis]	catalytic activity	2.16E-35
	gi|357479451	hypothetical protein MTR_4g126940 [Medicago truncatula]	Null	2.19E-07
	gi|388497358	unknown [Lotus japonicus]	cell	6.36E-140
	gi|403330330	B-class lysophosphatidate acyltransferase [Ricinus communis]	Null	1.38E-48
	gi|408384444	sucrose transporter 1 [Fragaria x ananassa]	Null	8.06E-06
	gi|462402785	hypothetical protein PRUPE_ppa001632mg [Prunus persica]	cell	2.41E-159
	gi|462424288	hypothetical protein PRUPE_ppa001149mg [Prunus persica]	metabolic process	2.49E-90
	gi|470102552	PREDICTED: glutamate receptor 3.3-like [Fragaria vesca subsp. vesca]	transporter activity	0
	gi|470103475	PREDICTED: LMBR1 domain-containing protein 2 homolog A-like [Fragaria vesca subsp. vesca]	membrane part	0
	gi|470104046	PREDICTED: uncharacterized protein LOC101295564 [Fragaria vesca subsp. vesca]	Null	7.17E-92
	gi|470104123	PREDICTED: major facilitator superfamily domain-containing protein 5-like [Fragaria vesca subsp. vesca]	Null	3.91E-10
	gi|470104232	PREDICTED: mechanosensitive ion channel protein 2, chloroplastic-like [Fragaria vesca subsp. vesca]	cell	0
	gi|470104313	PREDICTED: agamous-like MADS-box protein AGL21-like [Fragaria vesca subsp. vesca]	metabolic process	1.29E-77
	gi|470104580	PREDICTED: CST complex subunit CTC1-like [Fragaria vesca subsp. vesca]	organelle part	0
	gi|470105207	PREDICTED: auxin-binding protein ABP19a-like [Fragaria vesca subsp. vesca]	Null	4.62E-101
	gi|470105348	PREDICTED: probable acyl-activating enzyme 1, peroxisomal-like [Fragaria vesca subsp. vesca]	catalytic activity	7.38E-28
	gi|470106375	PREDICTED: long chain acyl-CoA synthetase 1-like [Fragaria vesca subsp. vesca]	cell	0
	gi|470107392	PREDICTED: 26S proteasome regulatory subunit 4 homolog A-like [Fragaria vesca subsp. vesca]	binding	0
	gi|470107767	PREDICTED: uncharacterized protein LOC101295670 [Fragaria vesca subsp. vesca]	Null	5.50E-64
	gi|470107938	PREDICTED: fatty acid amide hydrolase-like [Fragaria vesca subsp. vesca]	cell	7.06E-134
	gi|470108286	PREDICTED: putative laccase-9-like [Fragaria vesca subsp. vesca]	catalytic activity	0

gi|470108351	PREDICTED: uncharacterized protein LOC101309208 [Fragaria vesca subsp. vesca]	Null	3.62E-68
gi|470110283	PREDICTED: uncharacterized protein LOC101298087 [Fragaria vesca subsp. vesca]	reproductive process	0
gi|470110450	PREDICTED: transcriptional corepressor LEUNIG-like [Fragaria vesca subsp. vesca]	biological regulation	0
gi|470110548	PREDICTED: pleiotropic drug resistance protein 2-like [Fragaria vesca subsp. vesca]	membrane	0
gi|470110562	PREDICTED: tRNA dimethylallyltransferase 2-like [Fragaria vesca subsp. vesca]	binding	5.19E-160
gi|470111010	PREDICTED: chromodomain-helicase-DNA-binding protein 2-like [Fragaria vesca subsp. vesca]	binding	0
gi|470111415	PREDICTED: LOW QUALITY PROTEIN: CHD3-type chromatin-remodeling factor PICKLE-like [Fragaria vesca subsp. vesca]	Null	7.16E-09
gi|470111572	PREDICTED: E3 SUMO-protein ligase MMS21-like [Fragaria vesca subsp. vesca]	binding	5.91E-58
gi|470111832	PREDICTED: agamous-like MADS-box protein AGL62-like [Fragaria vesca subsp. vesca]	Null	5.13E-14
gi|470112000	PREDICTED: phospholipid--sterol O-acyltransferase-like [Fragaria vesca subsp. vesca]	single-organism process	0
gi|470112161	PREDICTED: niemann-Pick C1 protein-like [Fragaria vesca subsp. vesca]	receptor activity	0
gi|470112557	PREDICTED: uncharacterized protein LOC101304542 [Fragaria vesca subsp. vesca]	Null	3.01E-10
gi|470112626	PREDICTED: SKP1-interacting partner 15-like [Fragaria vesca subsp. vesca]	binding	3.39E-32
gi|470113467	PREDICTED: uncharacterized protein LOC101308350 [Fragaria vesca subsp. vesca]	Null	0
gi|470113876	PREDICTED: uncharacterized protein LOC101310963 [Fragaria vesca subsp. vesca]	Null	6.43E-117
gi|470114978	PREDICTED: serine/threonine-protein kinase CBK1-like [Fragaria vesca subsp. vesca]	binding	6.83E-177
gi|470115141	PREDICTED: mediator of RNA polymerase II transcription subunit 15a-like [Fragaria vesca subsp. vesca]	cellular process	1.60E-98
gi|470115548	PREDICTED: probable LRR receptor-like serine/threonine-protein kinase At2g24230-like [Fragaria vesca subsp. vesca]	catalytic activity	3.11E-21
gi|470115822	PREDICTED: cyclin-T1-3-like [Fragaria vesca subsp. vesca]	Null	0
gi|470116593	PREDICTED: zinc finger CCCH domain-containing protein 18-like [Fragaria vesca subsp. vesca]	catalytic activity	1.52E-57
gi|470117274	PREDICTED: rab3 GTPase-activating protein catalytic subunit-like [Fragaria vesca subsp. vesca]	cell	0
gi|470117847	PREDICTED: probable prefoldin subunit 3-like isoform 1 [Fragaria vesca subsp. vesca]	cell	7.25E-95
gi|470118066	PREDICTED: omega-6 fatty acid desaturase, endoplasmic reticulum isozyme 2-like [Fragaria vesca subsp. vesca]	catalytic activity	0

gi|470118839	PREDICTED: peroxidase 7-like [Fragaria vesca subsp. vesca]	antioxidant activity	0
gi|470119045	PREDICTED: CASP-like protein POPTRDRAFT_810994-like [Fragaria vesca subsp. vesca]	Null	1.05E-42
gi|470119329	PREDICTED: alternative NAD(P)H dehydrogenase 2, mitochondrial-like [Fragaria vesca subsp. vesca]	catalytic activity	0
gi|470119480	PREDICTED: 1,4-alpha-glucan-branching enzyme 3, chloroplastic/amyloplastic-like [Fragaria vesca subsp. vesca]	cell	0
gi|470120335	PREDICTED: uncharacterized protein LOC101294161 [Fragaria vesca subsp. vesca]	Null	6.67E-15
gi|470120371	PREDICTED: uncharacterized protein LOC101301378 [Fragaria vesca subsp. vesca]	Null	1.08E-95
gi|470120412	PREDICTED: pentatricopeptide repeat-containing protein At1g63130, mitochondrial-like [Fragaria vesca subsp. vesca]	Null	1.71E-38
gi|470120476	PREDICTED: cellulose synthase-like protein E1-like [Fragaria vesca subsp. vesca]	membrane	0
gi|470120882	PREDICTED: uncharacterized protein LOC101302153 [Fragaria vesca subsp. vesca]	cell	0
gi|470121136	PREDICTED: uncharacterized protein LOC101292007 [Fragaria vesca subsp. vesca]	Null	0
gi|470121443	PREDICTED: squamosa promoter-binding-like protein 7-like [Fragaria vesca subsp. vesca]	biological regulation	0
gi|470121947	PREDICTED: pentatricopeptide repeat-containing protein At5g18950-like [Fragaria vesca subsp. vesca]	Null	3.28E-07
gi|470122093	PREDICTED: squamosa promoter-binding-like protein 12-like [Fragaria vesca subsp. vesca]	Null	0
gi|470122923	PREDICTED: protein MEI2-like 4-like [Fragaria vesca subsp. vesca]	binding	0
gi|470122977	PREDICTED: RNA pseudourine synthase 7-like [Fragaria vesca subsp. vesca]	catalytic activity	1.07E-178
gi|470123448	PREDICTED: uncharacterized protein YpgQ-like [Fragaria vesca subsp. vesca]	catalytic activity	6.08E-115
gi|470123487	PREDICTED: pre-mRNA-processing protein 40C-like [Fragaria vesca subsp. vesca]	binding	0
gi|470123941	PREDICTED: uncharacterized protein LOC101305167 [Fragaria vesca subsp. vesca]	Null	6.36E-145
gi|470124027	PREDICTED: sulfate transporter 3.1-like [Fragaria vesca subsp. vesca]	transporter activity	6.10E-19
gi|470124060	PREDICTED: uncharacterized protein LOC101304182 [Fragaria vesca subsp. vesca]	Null	0
gi|470124141	PREDICTED: uncharacterized protein LOC101298599 [Fragaria vesca subsp. vesca]	Null	1.21E-23
gi|470124193	PREDICTED: CASP-like protein 6-like [Fragaria vesca subsp. vesca]	Null	2.20E-10
gi|470124363	PREDICTED: putative ribonuclease H protein At1g65750-like [Fragaria vesca subsp. vesca]	Null	2.50E-35
gi|470124647	PREDICTED: importin-9-like [Fragaria vesca subsp. vesca]	localization	0

gi|470125065	PREDICTED: F-box/kelch-repeat protein At3g06240-like [Fragaria vesca subsp. vesca]	Null	0
gi|470125132	PREDICTED: uncharacterized protein LOC101298513 [Fragaria vesca subsp. vesca]	Null	0
gi|470125487	PREDICTED: SPX domain-containing membrane protein At4g22990-like [Fragaria vesca subsp. vesca]	localization	0
gi|470125561	PREDICTED: uncharacterized protein LOC101297726 [Fragaria vesca subsp. vesca]	Null	6.48E-07
gi|470127533	PREDICTED: universal stress protein A-like protein-like [Fragaria vesca subsp. vesca]	response to stimulus	4.65E-73
gi|470127670	PREDICTED: ABC transporter I family member 17-like [Fragaria vesca subsp. vesca]	binding	4.37E-130
gi|470127956	PREDICTED: L-ascorbate oxidase-like [Fragaria vesca subsp. vesca]	catalytic activity	1.75E-76
gi|470127997	PREDICTED: phosphatidylinositol 4-phosphate 5-kinase 8-like [Fragaria vesca subsp. vesca]	binding	0
gi|470128218	PREDICTED: tuftelin-interacting protein 11-like [Fragaria vesca subsp. vesca]	Null	3.90E-20
gi|470128576	PREDICTED: uncharacterized protein LOC101299868 [Fragaria vesca subsp. vesca]	catalytic activity	9.37E-145
gi|470128592	PREDICTED: uncharacterized protein LOC101302362 [Fragaria vesca subsp. vesca]	membrane part	2.40E-112
gi|470128687	PREDICTED: uncharacterized protein LOC101292215 [Fragaria vesca subsp. vesca]	Null	4.96E-40
gi|470129551	PREDICTED: serine/threonine-protein kinase PBS1-like [Fragaria vesca subsp. vesca]	binding	3.37E-130
gi|470130900	PREDICTED: uncharacterized protein LOC101299872 [Fragaria vesca subsp. vesca]	Null	4.42E-39
gi|470131227	PREDICTED: ATP-dependent DNA helicase Q-like 4A-like [Fragaria vesca subsp. vesca]	response to stimulus	0
gi|470131247	PREDICTED: anaphase-promoting complex subunit 2-like [Fragaria vesca subsp. vesca]	cell	0
gi|470131291	PREDICTED: purple acid phosphatase 23-like [Fragaria vesca subsp. vesca]	catalytic activity	0
gi|470131344	PREDICTED: uncharacterized protein LOC101305082 [Fragaria vesca subsp. vesca]	Null	0
gi|470131673	PREDICTED: ATP synthase subunit delta', mitochondrial-like [Fragaria vesca subsp. vesca]	macromolecular complex	9.77E-95
gi|470131796	PREDICTED: putative F-box protein At1g67390-like [Fragaria vesca subsp. vesca]	Null	0
gi|470132062	PREDICTED: uncharacterized protein LOC101293797 [Fragaria vesca subsp. vesca]	Null	1.49E-47
gi|470132102	PREDICTED: uncharacterized protein LOC101300455 [Fragaria vesca subsp. vesca]	Null	0
gi|470132228	PREDICTED: decapping 5-like protein-like [Fragaria vesca subsp. vesca]	Null	0
gi|470132258	PREDICTED: WD repeat-containing protein 48 homolog [Fragaria vesca subsp. vesca]	catalytic activity	0
gi|470134516	PREDICTED: GTP-binding protein TypA/BipA homolog [Fragaria vesca subsp. vesca]	catalytic activity	2.46E-115

gi|470134556	PREDICTED: transcription activator GLK1-like [Fragaria vesca subsp. vesca]	Null	0
gi|470134558	PREDICTED: serine carboxypeptidase-like 40-like [Fragaria vesca subsp. vesca]	catalytic activity	0
gi|470135119	PREDICTED: protein RRP5 homolog [Fragaria vesca subsp. vesca]	organelle part	0
gi|470135695	PREDICTED: uncharacterized protein At2g41620-like [Fragaria vesca subsp. vesca]	organelle part	0
gi|470136327	PREDICTED: probable S-acyltransferase At4g15080-like [Fragaria vesca subsp. vesca]	membrane	0
gi|470136500	PREDICTED: cyclin-G-associated kinase-like [Fragaria vesca subsp. vesca]	multi-organism process	9.14E-167
gi|470137167	PREDICTED: LOW QUALITY PROTEIN: ABC transporter A family member 1-like [Fragaria vesca subsp. vesca]	binding	0
gi|470137560	PREDICTED: transcription factor TCP20-like [Fragaria vesca subsp. vesca]	Null	3.34E-117
gi|470137699	PREDICTED: uncharacterized protein LOC101313912 [Fragaria vesca subsp. vesca]	cellular process	0
gi|470138030	PREDICTED: PHD finger protein MALE MEIOCYTE DEATH 1-like [Fragaria vesca subsp. vesca]	cellular component organization or biogenesis	0
gi|470139380	PREDICTED: double-stranded RNA-binding protein 1-like [Fragaria vesca subsp. vesca]	Null	1.61E-06
gi|470139591	PREDICTED: E3 ubiquitin-protein ligase SINAT2-like [Fragaria vesca subsp. vesca]	catalytic activity	4.95E-143
gi|470139593	PREDICTED: probable protein kinase At2g41970-like [Fragaria vesca subsp. vesca]	binding	0
gi|470139694	PREDICTED: uncharacterized protein LOC101301607 [Fragaria vesca subsp. vesca]	Null	0
gi|470140857	PREDICTED: uncharacterized protein LOC101301323 [Fragaria vesca subsp. vesca]	binding	1.48E-18
gi|470140965	PREDICTED: cytoplasmic tRNA 2-thiolation protein 2-like [Fragaria vesca subsp. vesca]	Null	0
gi|470141148	PREDICTED: F-box/kelch-repeat protein At3g23880-like [Fragaria vesca subsp. vesca]	Null	5.43E-15
gi|470141550	PREDICTED: callose synthase 2-like [Fragaria vesca subsp. vesca]	membrane	0
gi|470141620	PREDICTED: thylakoid lumenal 19 kDa protein, chloroplastic-like [Fragaria vesca subsp. vesca]	cell	3.13E-71
gi|470141927	PREDICTED: histone-lysine N-methyltransferase ATX3-like [Fragaria vesca subsp. vesca]	catalytic activity	0
gi|470143095	PREDICTED: cytochrome b5 reductase 4-like [Fragaria vesca subsp. vesca]	multicellular organismal process	3.91E-97

	gi|470143162	PREDICTED: rac-like GTP-binding protein 7-like [Fragaria vesca subsp. vesca]	cell	1.97E-58
	gi|470143700	PREDICTED: protein kinase APK1B, chloroplastic-like [Fragaria vesca subsp. vesca]	Null	2.48E-18
	gi|470145221	PREDICTED: G-type lectin S-receptor-like serine/threonine-protein kinase B120-like [Fragaria vesca subsp. vesca]	catalytic activity	1.42E-37
	gi|470146022	PREDICTED: uncharacterized protein LOC101315004 [Fragaria vesca subsp. vesca]	Null	1.25E-59
	gi|470146181	PREDICTED: putative ribonuclease H protein At1g65750-like [Fragaria vesca subsp. vesca]	Null	9.58E-11
	gi|470147163	PREDICTED: LOW QUALITY PROTEIN: ferric reduction oxidase 4-like, partial [Fragaria vesca subsp. vesca]	binding	1.59E-18
	gi|470147363	PREDICTED: NAC transcription factor ONAC010-like [Fragaria vesca subsp. vesca]	Null	1.21E-100
	gi|470147736	PREDICTED: LOW QUALITY PROTEIN: probable aspartyl aminopeptidase-like [Fragaria vesca subsp. vesca]	cell	1.48E-62
	gi|470148182	PREDICTED: helicase protein MOM1-like [Fragaria vesca subsp. vesca]	binding	0
	gi|470148343	PREDICTED: LOW QUALITY PROTEIN: CBS domain-containing protein CBSCBSPB3-like [Fragaria vesca subsp. vesca]	Null	0
	gi|470148479	PREDICTED: enzymatic polyprotein-like [Fragaria vesca subsp. vesca]	Null	7.47E-43
	gi|470148519	PREDICTED: F-box protein SKIP16-like [Fragaria vesca subsp. vesca]	binding	0
	gi|470148781	PREDICTED: uncharacterized protein LOC101304245 [Fragaria vesca subsp. vesca]	Null	0
	gi|62319939	hypothetical protein [Arabidopsis thaliana]	Null	3.68E-12
miR5290	gi|470112365	PREDICTED: leucine-rich repeat and death domain-containing protein 1-like [Fragaria vesca subsp. vesca]	Null	0
	gi|470113133	PREDICTED: 1-aminocyclopropane-1-carboxylate synthase CMA101-like [Fragaria vesca subsp. vesca]	cellular process	0
	gi|470129094	PREDICTED: probable beta-D-xylosidase 2-like [Fragaria vesca subsp. vesca]	cell	0
	gi|470132879	PREDICTED: GDSL esterase/lipase At1g09390-like [Fragaria vesca subsp. vesca]	organelle part	2.55E-176
miR5642	no target in F.vesca			
miR5643	gi|470148152	PREDICTED: uncharacterized protein LOC101311721 [Fragaria vesca subsp. vesca]	multicellular organismal process	1.83E-112
miR5656	no target in F.vesca			
miR5663	no target in F.vesca			
miR5813	no target in F.vesca			

miR6103	gi|470147075	PREDICTED: probable phosphatidylinositol 4-kinase type 2-beta At1g26270-like [Fragaria vesca subsp. vesca]	catalytic activity	4.42E-78
miR6437	no target in F.vesca			
miR7708	no target in F.vesca			
miR7732	no target in F.vesca			
miR7767	gi|470112213	PREDICTED: linoleate 9S-lipoxygenase 6-like [Fragaria vesca subsp. vesca]	catalytic activity	0
	gi|470125218	PREDICTED: uncharacterized protein LOC101312724 [Fragaria vesca subsp. vesca]	Null	0
	gi|470136547	PREDICTED: protein unc-50 homolog [Fragaria vesca subsp. vesca]	Null	2.74E-13
	gi|470141108	PREDICTED: serine carboxypeptidase II-3-like [Fragaria vesca subsp. vesca]	Null	0
miR7782	no target in F.vesca			
miR7984	gi|470118845	PREDICTED: pre-mRNA-splicing factor ATP-dependent RNA helicase PRP16-like [Fragaria vesca subsp. vesca]	binding	0
	gi|470148846	PREDICTED: uncharacterized protein LOC101292659 [Fragaria vesca subsp. vesca]	Null	5.19E-162
miR8122	gi|470110995	PREDICTED: uncharacterized protein LOC101306203 [Fragaria vesca subsp. vesca]	binding	0
	gi|470113449	PREDICTED: subtilisin-like protease-like [Fragaria vesca subsp. vesca]	catalytic activity	0
	gi|470114425	PREDICTED: nuclear inhibitor of protein phosphatase 1-like [Fragaria vesca subsp. vesca]	Null	0
miR8175	gi|255561482	Squalene monooxygenase, putative [Ricinus communis]	binding	5.67E-129
	gi|470105547	PREDICTED: ATP-dependent DNA helicase DDM1-like [Fragaria vesca subsp. vesca]	catalytic activity	0
	gi|470126560	PREDICTED: putative phospholipid-transporting ATPase 9-like [Fragaria vesca subsp. vesca]	binding	0
	gi|470129464	PREDICTED: geraniol 8-hydroxylase-like [Fragaria vesca subsp. vesca]	catalytic activity	0
	gi|470132307	PREDICTED: uncharacterized protein LOC101296394 [Fragaria vesca subsp. vesca]	Null	3.27E-171
	gi|470136597	PREDICTED: serine/threonine-protein kinase WNK8-like [Fragaria vesca subsp. vesca]	catalytic activity	0
	gi|470143625	PREDICTED: uncharacterized protein LOC101299814 [Fragaria vesca subsp. vesca]	Null	0
	gi|470146462	PREDICTED: uncharacterized protein LOC101314710 [Fragaria vesca subsp. vesca]	catalytic activity	0
	gi|470147629	PREDICTED: aquaporin NIP1-2-like [Fragaria vesca subsp. vesca]	membrane part	7.49E-153
	gi|470149290	PREDICTED: uncharacterized protein LOC101314053 [Fragaria vesca subsp. vesca]	Null	0
miR8183	gi|470127869	PREDICTED: threonylcarbamoyladenosine tRNA methylthiotransferase-like [Fragaria vesca subsp. vesca]	cellular process	0
miR8581	no target in F.vesca			

miR8666	no target in F.vesca			
miR8781	no target in F.vesca			
miR9472	gi|241993361	putative gag-pol polyprotein [Fragaria x ananassa]	binding	8.33E-70
	gi|462407656	hypothetical protein PRUPE_ppa006343mg [Prunus persica]	Null	3.17E-06
	gi|470102876	PREDICTED: GTPase Era-like [Fragaria vesca subsp. vesca]	membrane	0
	gi|470105505	PREDICTED: putative pentatricopeptide repeat-containing protein At3g47840-like [Fragaria vesca subsp. vesca]	binding	9.36E-33
	gi|470116581	PREDICTED: protein TORNADO 1-like [Fragaria vesca subsp. vesca]	cell	1.93E-106
	gi|470130008	PREDICTED: trichome differentiation protein GL1-like [Fragaria vesca subsp. vesca]	cell	8.37E-132
miR9653	gi|462409529	hypothetical protein PRUPE_ppa001072mg [Prunus persica]	Null	0
	gi|470105765	PREDICTED: bifunctional 3-dehydroquinate dehydratase/shikimate dehydrogenase, chloroplastic-like [Fragaria vesca subsp. vesca]	catalytic activity	0
	gi|470110844	PREDICTED: uncharacterized protein LOC101308047 [Fragaria vesca subsp. vesca]	binding	2.47E-128
	gi|470113023	PREDICTED: histone-lysine N-methyltransferase ASHH2-like [Fragaria vesca subsp. vesca]	biological regulation	0
	gi|470131876	PREDICTED: protein NLP7-like [Fragaria vesca subsp. vesca]	cell	0
	gi|470147169	PREDICTED: protein TRANSPARENT TESTA 12-like [Fragaria vesca subsp. vesca]	multicellular organismal process	0
	gi|470147298	PREDICTED: TMV resistance protein N-like [Fragaria vesca subsp. vesca]	Null	1.14E-175
miR9767	gi|422293454	transcript antisense to ribosomal rna protein [Nannochloropsis gaditana CCMP526]	Null	1.02E-26

Table S5: Targets of identified novel miRNAs in infected strawberry fruits

Name	Target gene No.	Target gene description	Possible function	E value
fan-novel-001	gi|147860024	hypothetical protein VITISV_015109 [Vitis vinifera]	Null	0
	gi|359485158	PREDICTED: serine/threonine-protein phosphatase 5-like [Vitis vinifera]	cellular process	0
	gi|470121825	PREDICTED: LOW QUALITY PROTEIN: DNA repair protein RAD50-like [Fragaria vesca subsp. vesca]	cellular component organization or biogenesis	0
	gi|470125477	PREDICTED: putative copper-transporting ATPase HMA5-like [Fragaria vesca subsp. vesca]	binding	0
	gi|470145158	PREDICTED: putative disease resistance protein At3g14460-like [Fragaria vesca subsp. vesca]	cell	1.93E-43
fan-novel-002	no target in F.vesca			
fan-novel-003	gi|470133245	PREDICTED: uncharacterized protein LOC101312361 [Fragaria vesca subsp. vesca]	Null	2.19E-152
	gi|470146238	PREDICTED: MLO-like protein 1-like [Fragaria vesca subsp. vesca]	membrane	0
	gi|470147427	PREDICTED: capsid protein VP1-like, partial [Fragaria vesca subsp. vesca]	Null	0
fan-novel-004	gi|470141497	PREDICTED: transcription factor GAMYB-like [Fragaria vesca subsp. vesca]	Null	0
fan-novel-005	no target in F.vesca			
fan-novel-006	gi|462403251	hypothetical protein PRUPE_ppa018907mg, partial [Prunus persica]	Null	3.12E-42
	gi|470141705	PREDICTED: ATP-dependent zinc metalloprotease FTSH 12, chloroplastic-like [Fragaria vesca subsp. vesca]	cell	0
fan-novel-007	gi|147804870	hypothetical protein VITISV_005133 [Vitis vinifera]	Null	8.61E-09
	gi|147820415	hypothetical protein VITISV_008274 [Vitis vinifera]	Null	3.90E-16
	gi|156763844	putative reductase [Nicotiana tabacum]	cellular process	2.27E-90
	gi|225437408	PREDICTED: uncharacterized protein LOC100246950 [Vitis vinifera]	Null	5.24E-19
	gi|225443998	PREDICTED: ABC transporter C family member 2-like [Vitis vinifera]	catalytic activity	0
	gi|255578422	monoxygenase, putative [Ricinus communis]	catalytic activity	2.16E-35
	gi|356535141	PREDICTED: protein MON2 homolog isoform 2 [Glycine max]	Null	0

gi|357479451	hypothetical protein MTR_4g126940 [Medicago truncatula]	Null	2.19E-07
gi|388497358	unknown [Lotus japonicus]	cell	6.36E-140
gi|403330330	B-class lysophosphatidate acyltransferase [Ricinus communis]	Null	1.38E-48
gi|408384444	sucrose transporter 1 [Fragaria x ananassa]	Null	2.54E-08
gi|462399497	hypothetical protein PRUPE_ppa023647mg [Prunus persica]	transporter activity	1.10E-169
gi|462399766	hypothetical protein PRUPE_ppa002529mg [Prunus persica]	cell	1.63E-178
gi|462402785	hypothetical protein PRUPE_ppa001632mg [Prunus persica]	cell	2.41E-159
gi|462404779	hypothetical protein PRUPE_ppb020052mg [Prunus persica]	Null	7.54E-36
gi|462424288	hypothetical protein PRUPE_ppa001149mg [Prunus persica]	metabolic process	2.49E-90
gi|470102552	PREDICTED: glutamate receptor 3.3-like [Fragaria vesca subsp. vesca]	transporter activity	0
gi|470103475	PREDICTED: LMBR1 domain-containing protein 2 homolog A-like [Fragaria vesca subsp. vesca]	membrane part	0
gi|470104046	PREDICTED: uncharacterized protein LOC101295564 [Fragaria vesca subsp. vesca]	Null	7.17E-92
gi|470104123	PREDICTED: major facilitator superfamily domain-containing protein 5-like [Fragaria vesca subsp. vesca]	Null	3.91E-10
gi|470104232	PREDICTED: mechanosensitive ion channel protein 2, chloroplastic-like [Fragaria vesca subsp. vesca]	cell	0
gi|470104313	PREDICTED: agamous-like MADS-box protein AGL21-like [Fragaria vesca subsp. vesca]	metabolic process	1.29E-77
gi|470104580	PREDICTED: CST complex subunit CTC1-like [Fragaria vesca subsp. vesca]	organelle part	0
gi|470104869	PREDICTED: receptor-like serine/threonine-protein kinase SD1-8-like [Fragaria vesca subsp. vesca]	Null	7.88E-13
gi|470105207	PREDICTED: auxin-binding protein ABP19a-like [Fragaria vesca subsp. vesca]	Null	4.62E-101
gi|470105348	PREDICTED: probable acyl-activating enzyme 1, peroxisomal-like [Fragaria vesca subsp. vesca]	catalytic activity	7.38E-28
gi|470105901	PREDICTED: probable LRR receptor-like serine/threonine-protein kinase At1g56130-like [Fragaria vesca subsp. vesca]	membrane	0
gi|470106216	PREDICTED: transcription factor WER-like [Fragaria vesca subsp. vesca]	Null	2.57E-145
gi|470106375	PREDICTED: long chain acyl-CoA synthetase 1-like [Fragaria vesca subsp. vesca]	cell	0
gi|470107178	PREDICTED: hyoscyamine 6-dioxygenase-like [Fragaria vesca subsp. vesca]	Null	0
gi|470107392	PREDICTED: 26S proteasome regulatory subunit 4 homolog A-like [Fragaria vesca subsp. vesca]	binding	0
gi|470107767	PREDICTED: uncharacterized protein LOC101295670 [Fragaria vesca subsp. vesca]	Null	5.50E-64

gi|470108224	PREDICTED: uncharacterized membrane protein At4g09580-like [Fragaria vesca subsp. vesca]	cell	1.35E-138
gi|470108277	PREDICTED: probable serine/threonine-protein kinase At1g18390-like isoform 1 [Fragaria vesca subsp. vesca]	binding	0
gi|470108286	PREDICTED: putative laccase-9-like [Fragaria vesca subsp. vesca]	catalytic activity	0
gi|470108351	PREDICTED: uncharacterized protein LOC101309208 [Fragaria vesca subsp. vesca]	Null	3.62E-68
gi|470108469	PREDICTED: DNA-directed RNA polymerases I, II, and III subunit RPABC2-like [Fragaria vesca subsp. vesca]	organelle part	2.62E-58
gi|470110283	PREDICTED: uncharacterized protein LOC101298087 [Fragaria vesca subsp. vesca]	reproductive process	0
gi|470110450	PREDICTED: transcriptional corepressor LEUNIG-like [Fragaria vesca subsp. vesca]	biological regulation	0
gi|470110513	PREDICTED: ubiquitin carboxyl-terminal hydrolase 8-like [Fragaria vesca subsp. vesca]	catalytic activity	0
gi|470110548	PREDICTED: pleiotropic drug resistance protein 2-like [Fragaria vesca subsp. vesca]	membrane	0
gi|470110562	PREDICTED: tRNA dimethylallyltransferase 2-like [Fragaria vesca subsp. vesca]	binding	5.19E-160
gi|470111010	PREDICTED: chromodomain-helicase-DNA-binding protein 2-like [Fragaria vesca subsp. vesca]	binding	0
gi|470111415	PREDICTED: LOW QUALITY PROTEIN: CHD3-type chromatin-remodeling factor PICKLE-like [Fragaria vesca subsp. vesca]	Null	7.16E-09
gi|470111572	PREDICTED: E3 SUMO-protein ligase MMS21-like [Fragaria vesca subsp. vesca]	binding	5.91E-58
gi|470111832	PREDICTED: agamous-like MADS-box protein AGL62-like [Fragaria vesca subsp. vesca]	Null	5.13E-14
gi|470112000	PREDICTED: phospholipid--sterol O-acyltransferase-like [Fragaria vesca subsp. vesca]	single-organism process	0
gi|470112161	PREDICTED: niemann-Pick C1 protein-like [Fragaria vesca subsp. vesca]	receptor activity	0
gi|470112557	PREDICTED: uncharacterized protein LOC101304542 [Fragaria vesca subsp. vesca]	Null	3.01E-10
gi|470112626	PREDICTED: SKP1-interacting partner 15-like [Fragaria vesca subsp. vesca]	binding	3.39E-32
gi|470113467	PREDICTED: uncharacterized protein LOC101308350 [Fragaria vesca subsp. vesca]	Null	0
gi|470113876	PREDICTED: uncharacterized protein LOC101310963 [Fragaria vesca subsp. vesca]	Null	6.43E-117
gi|470114493	PREDICTED: uncharacterized protein LOC101314729 [Fragaria vesca subsp. vesca]	binding	0
gi|470114978	PREDICTED: serine/threonine-protein kinase CBK1-like [Fragaria vesca subsp. vesca]	binding	6.83E-177
gi|470115141	PREDICTED: mediator of RNA polymerase II transcription subunit 15a-like [Fragaria vesca subsp. vesca]	cellular process	1.60E-98

gi|470115548	PREDICTED: probable LRR receptor-like serine/threonine-protein kinase At2g24230-like [Fragaria vesca subsp. vesca]	catalytic activity	3.11E-21
gi|470115822	PREDICTED: cyclin-T1-3-like [Fragaria vesca subsp. vesca]	Null	0
gi|470115985	PREDICTED: bidirectional sugar transporter SWEET2-like [Fragaria vesca subsp. vesca]	membrane part	7.48E-80
gi|470116593	PREDICTED: zinc finger CCCH domain-containing protein 18-like [Fragaria vesca subsp. vesca]	catalytic activity	1.52E-57
gi|470117274	PREDICTED: rab3 GTPase-activating protein catalytic subunit-like [Fragaria vesca subsp. vesca]	cell	0
gi|470117847	PREDICTED: probable prefoldin subunit 3-like isoform 1 [Fragaria vesca subsp. vesca]	cell	7.25E-95
gi|470118066	PREDICTED: omega-6 fatty acid desaturase, endoplasmic reticulum isozyme 2-like [Fragaria vesca subsp. vesca]	catalytic activity	0
gi|470118132	PREDICTED: uncharacterized protein LOC101294639 [Fragaria vesca subsp. vesca]	Null	5.43E-10
gi|470118839	PREDICTED: peroxidase 7-like [Fragaria vesca subsp. vesca]	antioxidant activity	0
gi|470118983	PREDICTED: uncharacterized protein LOC101293382 [Fragaria vesca subsp. vesca]	Null	0
gi|470119045	PREDICTED: CASP-like protein POPTRDRAFT_810994-like [Fragaria vesca subsp. vesca]	Null	1.05E-42
gi|470119329	PREDICTED: alternative NAD(P)H dehydrogenase 2, mitochondrial-like [Fragaria vesca subsp. vesca]	catalytic activity	0
gi|470119480	PREDICTED: 1,4-alpha-glucan-branching enzyme 3, chloroplastic/amyloplastic-like [Fragaria vesca subsp. vesca]	cell	0
gi|470120335	PREDICTED: uncharacterized protein LOC101294161 [Fragaria vesca subsp. vesca]	Null	6.67E-15
gi|470120371	PREDICTED: uncharacterized protein LOC101301378 [Fragaria vesca subsp. vesca]	Null	1.08E-95
gi|470120412	PREDICTED: pentatricopeptide repeat-containing protein At1g63130, mitochondrial-like [Fragaria vesca subsp. vesca]	Null	1.71E-38
gi|470120476	PREDICTED: cellulose synthase-like protein E1-like [Fragaria vesca subsp. vesca]	membrane	0
gi|470120826	PREDICTED: uncharacterized protein LOC101293768 [Fragaria vesca subsp. vesca]	cell	6.72E-47
gi|470120882	PREDICTED: uncharacterized protein LOC101302153 [Fragaria vesca subsp. vesca]	cell	0
gi|470121136	PREDICTED: uncharacterized protein LOC101292007 [Fragaria vesca subsp. vesca]	Null	0
gi|470121443	PREDICTED: squamosa promoter-binding-like protein 7-like [Fragaria vesca subsp. vesca]	biological regulation	0
gi|470121947	PREDICTED: pentatricopeptide repeat-containing protein At5g18950-like [Fragaria vesca subsp. vesca]	Null	3.28E-07

gi|470122093	PREDICTED: squamosa promoter-binding-like protein 12-like [Fragaria vesca subsp. vesca]	Null	0
gi|470122923	PREDICTED: protein MEI2-like 4-like [Fragaria vesca subsp. vesca]	binding	0
gi|470122977	PREDICTED: RNA pseudourine synthase 7-like [Fragaria vesca subsp. vesca]	catalytic activity	1.07E-178
gi|470123353	PREDICTED: phospholipid-transporting ATPase 3-like [Fragaria vesca subsp. vesca]	binding	0
gi|470123448	PREDICTED: uncharacterized protein YpgQ-like [Fragaria vesca subsp. vesca]	catalytic activity	6.08E-115
gi|470123487	PREDICTED: pre-mRNA-processing protein 40C-like [Fragaria vesca subsp. vesca]	binding	0
gi|470123941	PREDICTED: uncharacterized protein LOC101305167 [Fragaria vesca subsp. vesca]	Null	6.36E-145
gi|470124027	PREDICTED: sulfate transporter 3.1-like [Fragaria vesca subsp. vesca]	transporter activity	6.10E-19
gi|470124060	PREDICTED: uncharacterized protein LOC101304182 [Fragaria vesca subsp. vesca]	Null	0
gi|470124141	PREDICTED: uncharacterized protein LOC101298599 [Fragaria vesca subsp. vesca]	Null	1.21E-23
gi|470124193	PREDICTED: CASP-like protein 6-like [Fragaria vesca subsp. vesca]	Null	2.20E-10
gi|470124363	PREDICTED: putative ribonuclease H protein At1g65750-like [Fragaria vesca subsp. vesca]	Null	2.50E-35
gi|470124647	PREDICTED: importin-9-like [Fragaria vesca subsp. vesca]	localization	0
gi|470125065	PREDICTED: F-box/kelch-repeat protein At3g06240-like [Fragaria vesca subsp. vesca]	Null	0
gi|470125132	PREDICTED: uncharacterized protein LOC101298513 [Fragaria vesca subsp. vesca]	Null	0
gi|470125487	PREDICTED: SPX domain-containing membrane protein At4g22990-like [Fragaria vesca subsp. vesca]	localization	0
gi|470125561	PREDICTED: uncharacterized protein LOC101297726 [Fragaria vesca subsp. vesca]	Null	6.48E-07
gi|470125778	PREDICTED: protein SPA1-RELATED 2-like [Fragaria vesca subsp. vesca]	cell	0
gi|470126429	PREDICTED: callose synthase 10-like [Fragaria vesca subsp. vesca]	Null	0
gi|470127533	PREDICTED: universal stress protein A-like protein-like [Fragaria vesca subsp. vesca]	response to stimulus	4.65E-73
gi|470127670	PREDICTED: ABC transporter I family member 17-like [Fragaria vesca subsp. vesca]	binding	4.37E-130
gi|470127956	PREDICTED: L-ascorbate oxidase-like [Fragaria vesca subsp. vesca]	Null	7.87E-122
gi|470127997	PREDICTED: phosphatidylinositol 4-phosphate 5-kinase 8-like [Fragaria vesca subsp. vesca]	binding	0
gi|470128047	PREDICTED: mitogen-activated protein kinase kinase kinase 1-like [Fragaria vesca subsp. vesca]	binding	3.66E-158
gi|470128218	PREDICTED: tuftelin-interacting protein 11-like [Fragaria vesca subsp. vesca]	Null	3.90E-20
gi|470128576	PREDICTED: uncharacterized protein LOC101299868 [Fragaria vesca subsp. vesca]	catalytic activity	9.37E-145

gi|470128592	PREDICTED: uncharacterized protein LOC101302362 [Fragaria vesca subsp. vesca]	membrane part	2.40E-112
gi|470128687	PREDICTED: uncharacterized protein LOC101292215 [Fragaria vesca subsp. vesca]	Null	4.96E-40
gi|470129551	PREDICTED: serine/threonine-protein kinase PBS1-like [Fragaria vesca subsp. vesca]	binding	3.37E-130
gi|470130900	PREDICTED: uncharacterized protein LOC101299872 [Fragaria vesca subsp. vesca]	Null	4.42E-39
gi|470131227	PREDICTED: ATP-dependent DNA helicase Q-like 4A-like [Fragaria vesca subsp. vesca]	response to stimulus	0
gi|470131247	PREDICTED: anaphase-promoting complex subunit 2-like [Fragaria vesca subsp. vesca]	cell	0
gi|470131291	PREDICTED: purple acid phosphatase 23-like [Fragaria vesca subsp. vesca]	catalytic activity	0
gi|470131344	PREDICTED: uncharacterized protein LOC101305082 [Fragaria vesca subsp. vesca]	Null	0
gi|470131363	PREDICTED: putative ribonuclease H protein At1g65750-like [Fragaria vesca subsp. vesca]	Null	2.04E-154
gi|470131673	PREDICTED: ATP synthase subunit delta', mitochondrial-like [Fragaria vesca subsp. vesca]	macromolecular complex	9.77E-95
gi|470131796	PREDICTED: putative F-box protein At1g67390-like [Fragaria vesca subsp. vesca]	Null	0
gi|470132062	PREDICTED: uncharacterized protein LOC101293797 [Fragaria vesca subsp. vesca]	Null	1.49E-47
gi|470132102	PREDICTED: uncharacterized protein LOC101300455 [Fragaria vesca subsp. vesca]	Null	0
gi|470132228	PREDICTED: decapping 5-like protein-like [Fragaria vesca subsp. vesca]	Null	0
gi|470132258	PREDICTED: WD repeat-containing protein 48 homolog [Fragaria vesca subsp. vesca]	catalytic activity	0
gi|470133347	PREDICTED: regulatory-associated protein of TOR 1-like [Fragaria vesca subsp. vesca]	growth	0
gi|470134024	PREDICTED: uncharacterized protein LOC101304108 [Fragaria vesca subsp. vesca]	Null	0
gi|470134504	PREDICTED: serine/threonine-protein kinase-like protein At3g51990-like [Fragaria vesca subsp. vesca]	binding	0
gi|470134516	PREDICTED: GTP-binding protein TypA/BipA homolog [Fragaria vesca subsp. vesca]	catalytic activity	2.46E-115
gi|470134556	PREDICTED: transcription activator GLK1-like [Fragaria vesca subsp. vesca]	Null	0
gi|470134558	PREDICTED: serine carboxypeptidase-like 40-like [Fragaria vesca subsp. vesca]	catalytic activity	0
gi|470135119	PREDICTED: protein RRP5 homolog [Fragaria vesca subsp. vesca]	organelle part	0
gi|470135695	PREDICTED: uncharacterized protein At2g41620-like [Fragaria vesca subsp. vesca]	organelle part	0
gi|470136327	PREDICTED: probable S-acyltransferase At4g15080-like [Fragaria vesca subsp. vesca]	membrane	0
gi|470136500	PREDICTED: cyclin-G-associated kinase-like [Fragaria vesca subsp. vesca]	multi-organism process	9.14E-167

gi|470137167	PREDICTED: LOW QUALITY PROTEIN: ABC transporter A family member 1-like [Fragaria vesca subsp. vesca]	binding	2.70E-74
gi|470137560	PREDICTED: transcription factor TCP20-like [Fragaria vesca subsp. vesca]	Null	3.34E-117
gi|470137699	PREDICTED: uncharacterized protein LOC101313912 [Fragaria vesca subsp. vesca]	cellular process	0
gi|470138030	PREDICTED: PHD finger protein MALE MEIOCYTE DEATH 1-like [Fragaria vesca subsp. vesca]	cellular component organization or biogenesis	0
gi|470139380	PREDICTED: double-stranded RNA-binding protein 1-like [Fragaria vesca subsp. vesca]	Null	1.61E-06
gi|470139591	PREDICTED: E3 ubiquitin-protein ligase SINAT2-like [Fragaria vesca subsp. vesca]	catalytic activity	4.95E-143
gi|470139593	PREDICTED: probable protein kinase At2g41970-like [Fragaria vesca subsp. vesca]	binding	0
gi|470139694	PREDICTED: uncharacterized protein LOC101301607 [Fragaria vesca subsp. vesca]	Null	0
gi|470139948	PREDICTED: cytokinin dehydrogenase 1-like [Fragaria vesca subsp. vesca]	binding	0
gi|470139954	PREDICTED: probable sugar phosphate/phosphate translocator At3g10290-like [Fragaria vesca subsp. vesca]	membrane part	3.08E-93
gi|470140611	PREDICTED: tricyclene synthase EBOS, chloroplastic-like [Fragaria vesca subsp. vesca]	cell	0
gi|470140857	PREDICTED: uncharacterized protein LOC101301323 [Fragaria vesca subsp. vesca]	binding	1.48E-18
gi|470140965	PREDICTED: cytoplasmic tRNA 2-thiolation protein 2-like [Fragaria vesca subsp. vesca]	Null	0
gi|470141148	PREDICTED: F-box/kelch-repeat protein At3g23880-like [Fragaria vesca subsp. vesca]	Null	5.43E-15
gi|470141526	PREDICTED: major facilitator superfamily domain-containing protein 12-like [Fragaria vesca subsp. vesca]	Null	1.19E-38
gi|470141550	PREDICTED: callose synthase 2-like [Fragaria vesca subsp. vesca]	membrane	0
gi|470141620	PREDICTED: thylakoid lumenal 19 kDa protein, chloroplastic-like [Fragaria vesca subsp. vesca]	cell	3.13E-71
gi|470141927	PREDICTED: histone-lysine N-methyltransferase ATX3-like [Fragaria vesca subsp. vesca]	catalytic activity	0
gi|470143095	PREDICTED: cytochrome b5 reductase 4-like [Fragaria vesca subsp. vesca]	multicellular organismal process	3.91E-97
gi|470143162	PREDICTED: rac-like GTP-binding protein 7-like [Fragaria vesca subsp. vesca]	cell	1.97E-58
gi|470143700	PREDICTED: protein kinase APK1B, chloroplastic-like [Fragaria vesca subsp. vesca]	Null	2.48E-18

	gi|470145221	PREDICTED: G-type lectin S-receptor-like serine/threonine-protein kinase B120-like [Fragaria vesca subsp. vesca]	catalytic activity	1.42E-37
	gi|470145936	PREDICTED: beta-galactosidase-like [Fragaria vesca subsp. vesca]	cell	0
	gi|470146022	PREDICTED: uncharacterized protein LOC101315004 [Fragaria vesca subsp. vesca]	Null	1.25E-59
	gi|470146095	PREDICTED: DNA-directed RNA polymerase III subunit RPC2-like [Fragaria vesca subsp. vesca]	catalytic activity	0
	gi|470146181	PREDICTED: putative ribonuclease H protein At1g65750-like [Fragaria vesca subsp. vesca]	Null	9.58E-11
	gi|470146700	PREDICTED: cation/H(+) antiporter 15-like [Fragaria vesca subsp. vesca]	Null	0
	gi|470147163	PREDICTED: LOW QUALITY PROTEIN: ferric reduction oxidase 4-like, partial [Fragaria vesca subsp. vesca]	binding	1.59E-18
	gi|470147363	PREDICTED: NAC transcription factor ONAC010-like [Fragaria vesca subsp. vesca]	Null	1.21E-100
	gi|470147736	PREDICTED: LOW QUALITY PROTEIN: probable aspartyl aminopeptidase-like [Fragaria vesca subsp. vesca]	cell	1.48E-62
	gi|470148182	PREDICTED: helicase protein MOM1-like [Fragaria vesca subsp. vesca]	binding	0
	gi|470148343	PREDICTED: LOW QUALITY PROTEIN: CBS domain-containing protein CBSCBSPB3-like [Fragaria vesca subsp. vesca]	Null	0
	gi|470148479	PREDICTED: enzymatic polyprotein-like [Fragaria vesca subsp. vesca]	Null	7.47E-43
	gi|470148519	PREDICTED: F-box protein SKIP16-like [Fragaria vesca subsp. vesca]	binding	0
	gi|470148781	PREDICTED: uncharacterized protein LOC101304245 [Fragaria vesca subsp. vesca]	Null	0
	gi|62319939	hypothetical protein [Arabidopsis thaliana]	Null	3.68E-12
fan-novel-008	no target in F.vesca			
fan-novel-009	no target in F.vesca			
fan-novel-010	gi|24461860	CTV.20 [Citrus trifoliata]	Null	3.90E-08
	gi|470142145	PREDICTED: probable aquaporin PIP1-4-like [Fragaria vesca subsp. vesca]	cell	2.92E-158
	gi|470146285	PREDICTED: monocopper oxidase-like protein SKU5-like [Fragaria vesca subsp. vesca]	binding	0
fan-novel-011	no target in F.vesca			
fan-novel-012	gi|470116204	PREDICTED: acyl-coenzyme A thioesterase 13-like [Fragaria vesca subsp. vesca]	Null	2.10E-82

fan-novel-013	gi|470105088	PREDICTED: replication protein A 70 kDa DNA-binding subunit-like [Fragaria vesca subsp. vesca]	Null	9.69E-98
	gi|470118068	PREDICTED: uncharacterized protein LOC101309521 [Fragaria vesca subsp. vesca]	Null	0
	gi|470124410	PREDICTED: TMV resistance protein N-like [Fragaria vesca subsp. vesca]	binding	8.89E-63
	gi|470124412	PREDICTED: leucine-rich repeat protein soc-2 homolog [Fragaria vesca subsp. vesca]	Null	0
	gi|470147937	PREDICTED: casein kinase I-like [Fragaria vesca subsp. vesca]	binding	0
fan-novel-014	gi|462404060	hypothetical protein PRUPE_ppa000373mg [Prunus persica]	localization	0
	gi|470112426	PREDICTED: zinc finger CCCH domain-containing protein 19-like [Fragaria vesca subsp. vesca]	Null	0
	gi|470126453	PREDICTED: DNA replication licensing factor mcm2-like [Fragaria vesca subsp. vesca]	binding	0
	gi|470133161	PREDICTED: root phototropism protein 3-like [Fragaria vesca subsp. vesca]	Null	6.39E-142
	gi|470138821	PREDICTED: putative disease resistance protein RGA1-like [Fragaria vesca subsp. vesca]	Null	0
	gi|470139011	PREDICTED: putative disease resistance protein RGA3-like [Fragaria vesca subsp. vesca]	Null	0
	gi|470141018	PREDICTED: putative disease resistance protein RGA3-like [Fragaria vesca subsp. vesca]	Null	0
fan-novel-015	gi|470105114	PREDICTED: DNA ligase 1-like [Fragaria vesca subsp. vesca]	response to stimulus	2.05E-47
	gi|470109667	PREDICTED: UDP-glucuronate:xylan alpha-glucuronosyltransferase 2-like [Fragaria vesca subsp. vesca]	cell	0
fan-novel-016	gi|462423111	hypothetical protein PRUPE_ppa015437mg, partial [Prunus persica]	Null	8.92E-07
fan-novel-017	gi|470105088	PREDICTED: replication protein A 70 kDa DNA-binding subunit-like [Fragaria vesca subsp. vesca]	Null	9.69E-98
	gi|470105935	PREDICTED: TMV resistance protein N-like [Fragaria vesca subsp. vesca]	Null	0
	gi|470105945	PREDICTED: protein SUPPRESSOR OF npr1-1, CONSTITUTIVE 1-like [Fragaria vesca subsp. vesca]	Null	0
	gi|470108366	PREDICTED: phytosulfokine receptor 2-like [Fragaria vesca subsp. vesca]	binding	0
	gi|470113037	PREDICTED: chalcone synthase 1-like [Fragaria vesca subsp. vesca]	cellular process	0
	gi|470118007	PREDICTED: serine/threonine-protein kinase TOUSLED-like [Fragaria vesca subsp. vesca]	cell	0
	gi|470119003	PREDICTED: protein lap4-like [Fragaria vesca subsp. vesca]	cell	0
	gi|470124410	PREDICTED: TMV resistance protein N-like [Fragaria vesca subsp. vesca]	cell	0
	gi|470124412	PREDICTED: leucine-rich repeat protein soc-2 homolog [Fragaria vesca subsp. vesca]	Null	0
fan-novel-018	gi|470119338	PREDICTED: pentatricopeptide repeat-containing protein At1g12300, mitochondrial-like [Fragaria vesca subsp. vesca]	Null	0

fan-novel-019	no target in F.vesca			
fan-novel-020	gi|470128495	PREDICTED: disease resistance protein RPM1-like [Fragaria vesca subsp. vesca]	Null	0
	gi|470140800	PREDICTED: putative disease resistance protein RGA3-like [Fragaria vesca subsp. vesca]	Null	0
	gi|470140824	PREDICTED: putative disease resistance protein RGA3-like [Fragaria vesca subsp. vesca]	Null	0
	gi|470147333	PREDICTED: putative disease resistance protein At3g14460-like [Fragaria vesca subsp. vesca]	Null	9.60E-167
fan-novel-021	gi|462412288	hypothetical protein PRUPE_ppa014994mg, partial [Prunus persica]	Null	3.55E-17
	gi|470128501	PREDICTED: disease resistance protein RPM1-like isoform 1 [Fragaria vesca subsp. vesca]	binding	0
	gi|470130928	PREDICTED: TMV resistance protein N-like [Fragaria vesca subsp. vesca]	Null	0
	gi|470131535	PREDICTED: disease resistance protein RPM1-like [Fragaria vesca subsp. vesca]	binding	0
	gi|470131568	PREDICTED: disease resistance protein RPM1-like [Fragaria vesca subsp. vesca]	binding	0
	gi|470133068	PREDICTED: protein RALF-like 33-like isoform 2 [Fragaria vesca subsp. vesca]	Null	3.44E-43
	gi|470146236	PREDICTED: DNA repair helicase XPB2-like [Fragaria vesca subsp. vesca]	Null	3.77E-06
fan-novel-022	gi|470110018	PREDICTED: F-box protein CPR30-like [Fragaria vesca subsp. vesca]	Null	0
	gi|470113332	PREDICTED: F-box protein CPR30-like [Fragaria vesca subsp. vesca]	Null	0
	gi|470120096	PREDICTED: F-box/kelch-repeat protein At3g06240-like [Fragaria vesca subsp. vesca]	Null	1.03E-26
	gi|470120240	PREDICTED: F-box/kelch-repeat protein At3g06240-like [Fragaria vesca subsp. vesca]	Null	0
	gi|470120242	PREDICTED: putative F-box protein At1g33530-like [Fragaria vesca subsp. vesca]	Null	1.83E-116
	gi|470124780	PREDICTED: F-box protein CPR30-like [Fragaria vesca subsp. vesca]	Null	0
	gi|470132776	PREDICTED: uncharacterized protein LOC101292514 [Fragaria vesca subsp. vesca]	Null	4.33E-91
	gi|470133967	PREDICTED: F-box/kelch-repeat protein At3g06240-like [Fragaria vesca subsp. vesca]	Null	0
	gi|470138987	PREDICTED: F-box protein CPR30-like [Fragaria vesca subsp. vesca]	Null	0
fan-novel-023	gi|470110018	PREDICTED: F-box protein CPR30-like [Fragaria vesca subsp. vesca]	Null	0
	gi|470113332	PREDICTED: F-box protein CPR30-like [Fragaria vesca subsp. vesca]	Null	0
	gi|470113334	PREDICTED: F-box protein CPR30-like [Fragaria vesca subsp. vesca]	Null	0
	gi|470113336	PREDICTED: F-box protein CPR30-like [Fragaria vesca subsp. vesca]	Null	0
	gi|470120240	PREDICTED: F-box/kelch-repeat protein At3g06240-like [Fragaria vesca subsp. vesca]	Null	0

	gi|470120242	PREDICTED: putative F-box protein At1g33530-like [Fragaria vesca subsp. vesca]	Null	1.83E-116
	gi|470124780	PREDICTED: F-box protein CPR30-like [Fragaria vesca subsp. vesca]	Null	0
	gi|470125267	PREDICTED: TIMELESS-interacting protein-like [Fragaria vesca subsp. vesca]	binding	2.40E-129
	gi|470130608	PREDICTED: putative pentatricopeptide repeat-containing protein At5g47460-like [Fragaria vesca subsp. vesca]	Null	0
	gi|470133965	PREDICTED: F-box/kelch-repeat protein At3g06240-like [Fragaria vesca subsp. vesca]	Null	0
	gi|470133967	PREDICTED: F-box/kelch-repeat protein At3g06240-like [Fragaria vesca subsp. vesca]	Null	0
	gi|470138983	PREDICTED: F-box protein CPR30-like [Fragaria vesca subsp. vesca]	Null	0
	gi|470138987	PREDICTED: F-box protein CPR30-like [Fragaria vesca subsp. vesca]	Null	0
	gi|470138995	PREDICTED: F-box protein CPR30-like [Fragaria vesca subsp. vesca]	Null	0
	gi|470138997	PREDICTED: F-box protein CPR30-like [Fragaria vesca subsp. vesca]	Null	0
	gi|470139003	PREDICTED: F-box protein CPR30-like [Fragaria vesca subsp. vesca]	Null	4.08E-174
	gi|470139005	PREDICTED: uncharacterized protein LOC101302966 [Fragaria vesca subsp. vesca]	Null	4.26E-69
fan-novel-024	gi|449446564	PREDICTED: 40S ribosomal protein S30-like isoform 1 [Cucumis sativus]	structural molecule activity	5.98E-22
	gi|470113815	PREDICTED: pentatricopeptide repeat-containing protein At2g22070-like [Fragaria vesca subsp. vesca]	Null	0
	gi|470122372	PREDICTED: squamosa promoter-binding-like protein 6-like [Fragaria vesca subsp. vesca]	Null	0
	gi|470123009	PREDICTED: squamosa promoter-binding-like protein 13A-like [Fragaria vesca subsp. vesca]	Null	0
	gi|470126622	PREDICTED: uncharacterized protein LOC101306334 [Fragaria vesca subsp. vesca]	Null	0
	gi|470129652	PREDICTED: probable leucine-rich repeat receptor-like protein kinase At5g63930-like [Fragaria vesca subsp. vesca]	membrane	0
	gi|470137245	PREDICTED: squamosa promoter-binding-like protein 17-like [Fragaria vesca subsp. vesca]	Null	0
	gi|470137555	PREDICTED: squamosa promoter-binding-like protein 13A-like [Fragaria vesca subsp. vesca]	Null	0
	gi|470138782	PREDICTED: putative ribonuclease H protein At1g65750-like [Fragaria vesca subsp. vesca]	Null	1.02E-153
	gi|470142120	PREDICTED: uncharacterized protein LOC101294106 [Fragaria vesca subsp. vesca]	organelle part	2.00E-145
	gi|470145723	PREDICTED: squamosa promoter-binding-like protein 7-like [Fragaria vesca subsp. vesca]	Null	6.24E-77

	gi|470149113	PREDICTED: squamosa promoter-binding-like protein 12-like [Fragaria vesca subsp. vesca]	Null	0
	gi|470149134	PREDICTED: uncharacterized protein LOC101314819 [Fragaria vesca subsp. vesca]	Null	1.18E-158
fan-novel-025	no target in F.vesca			
fan-novel-026	gi|470133967	PREDICTED: F-box/kelch-repeat protein At3g06240-like [Fragaria vesca subsp. vesca]	Null	0
	gi|470138983	PREDICTED: F-box protein CPR30-like [Fragaria vesca subsp. vesca]	Null	0
	gi|470139005	PREDICTED: uncharacterized protein LOC101302966 [Fragaria vesca subsp. vesca]	Null	4.26E-69
	gi|470139796	PREDICTED: F-box/kelch-repeat protein At3g06240-like [Fragaria vesca subsp. vesca]	Null	0
fan-novel-027	gi|470113332	PREDICTED: F-box protein CPR30-like [Fragaria vesca subsp. vesca]	Null	0
	gi|470113336	PREDICTED: F-box protein CPR30-like [Fragaria vesca subsp. vesca]	Null	0
	gi|470120096	PREDICTED: F-box/kelch-repeat protein At3g06240-like [Fragaria vesca subsp. vesca]	Null	1.03E-26
	gi|470120098	PREDICTED: F-box/kelch-repeat protein At3g06240-like [Fragaria vesca subsp. vesca]	Null	1.20E-22
	gi|470124780	PREDICTED: F-box protein CPR30-like [Fragaria vesca subsp. vesca]	Null	0
	gi|470139796	PREDICTED: F-box/kelch-repeat protein At3g06240-like [Fragaria vesca subsp. vesca]	Null	0
fan-novel-028	gi|470111693	PREDICTED: receptor-like protein kinase 2-like [Fragaria vesca subsp. vesca]	binding	0
	gi|470114901	PREDICTED: probable serine/threonine-protein kinase drkB-like isoform 2 [Fragaria vesca subsp. vesca]	binding	0
	gi|470118878	PREDICTED: putative pentatricopeptide repeat-containing protein At1g12700, mitochondrial-like [Fragaria vesca subsp. vesca]	Null	0
	gi|470125059	PREDICTED: pentatricopeptide repeat-containing protein At1g12300, mitochondrial-like [Fragaria vesca subsp. vesca]	Null	0
fan-novel-029	gi|470110004	PREDICTED: uncharacterized protein LOC101306694 [Fragaria vesca subsp. vesca]	Null	5.75E-123
	gi|470131289	PREDICTED: protein ROS1-like [Fragaria vesca subsp. vesca]	catalytic activity	0
fan-novel-030	gi|462395657	hypothetical protein PRUPE_ppa015021mg [Prunus persica]	Null	9.49E-32
fan-novel-031	gi|470115874	PREDICTED: transcription factor TCP4-like [Fragaria vesca subsp. vesca]	cellular process	0
	gi|470134668	PREDICTED: transcription factor TCP4-like [Fragaria vesca subsp. vesca]	Null	8.46E-175
	gi|470140559	PREDICTED: formimidoyltransferase-cyclodeaminase-like [Fragaria vesca subsp. vesca]	binding	4.29E-94
	gi|470141497	PREDICTED: transcription factor GAMYB-like [Fragaria vesca subsp. vesca]	Null	0

	gi|470143710	PREDICTED: glucan endo-1,3-beta-glucosidase-like [Fragaria vesca subsp. vesca]	binding	3.57E-35
fan-novel-032	gi|470115874	PREDICTED: transcription factor TCP4-like [Fragaria vesca subsp. vesca]	cellular process	0
	gi|470134668	PREDICTED: transcription factor TCP4-like [Fragaria vesca subsp. vesca]	Null	8.46E-175
	gi|470140559	PREDICTED: formimidoyltransferase-cyclodeaminase-like [Fragaria vesca subsp. vesca]	binding	4.29E-94
	gi|470141497	PREDICTED: transcription factor GAMYB-like [Fragaria vesca subsp. vesca]	Null	0
fan-novel-033	gi|470115874	PREDICTED: transcription factor TCP4-like [Fragaria vesca subsp. vesca]	cellular process	0
	gi|470134668	PREDICTED: transcription factor TCP4-like [Fragaria vesca subsp. vesca]	Null	8.46E-175
	gi|470141497	PREDICTED: transcription factor GAMYB-like [Fragaria vesca subsp. vesca]	Null	0
fan-novel-034	gi|470112365	PREDICTED: leucine-rich repeat and death domain-containing protein 1-like [Fragaria vesca subsp. vesca]	Null	0
	gi|470113133	PREDICTED: 1-aminocyclopropane-1-carboxylate synthase CMA101-like [Fragaria vesca subsp. vesca]	cellular process	0
	gi|470129094	PREDICTED: probable beta-D-xylosidase 2-like [Fragaria vesca subsp. vesca]	cell	0
	gi|470132879	PREDICTED: GDSL esterase/lipase At1g09390-like [Fragaria vesca subsp. vesca]	organelle part	2.55E-176
fan-novel-035	no target in F.vesca			

Table S6: Primer sequence of miRNA and *PIRL*

**miR166a**	RT-primer	ctcaactggtgtcgtggagtccggcaattcagttgagggggaatg
	RT-PCR primer	Forward：acactccagctgggtcggaccagg
		Reverse：aactggtgtcgtggag
	Probe	FAM-ttcagttgagggggaatg-TAMRA
**miR167**	RT-primer	ctcaactggtgtcgtggagtccggcaattcagttgagcagatcat
	RT-PCR primer	Forward: acactccagctgggtgaagctgcc
		Reverse: aactggtgtcgtggag
	Probe	FAM-ttcagttgagtagatcat-TAMRA
**miR5290a**	RT-primer	ctcaactggtgtcgtggagtccggcaattcagttgagcattgtct
	RT-PCR primer	Forward: acactccagctgggttggagagag
		Reverse: aactggtgtcgtggag
	Probe	FAM-ttcagttgagcattgtct-TAMRA
**fan-novel-018**	RT-primer	ctcaactggtgtcgtggagtccggcaattcagttgagaacagtca
	RT-PCR primer	Forward: acactccagctgggtctattcaaa
		Reverse: aactggtgtcgtggag
	Probe	FAM-ttcagttgagaacagtca-TAMRA
**fan-novel-020**	RT-primer	ctcaactggtgtcgtggagtccggcaattcagttgagggaatggc
	RT-PCR primer	Forward: acactccagctgggtctttcctag
		Reverse: aactggtgtcgtggag
	Probe	FAM-ttcagttgagggaatggc-TAMRA
**fan-novel-024**	RT-primer	ctcaactggtgtcgtggagtccggcaattcagttgaggagctcac
	RT-PCR primer	Forward: acactccagctgggtgacagaaga
		Reverse: aactggtgtcgtggag
	Probe	FAM-ttcagttgaggagctcac-TAMRA
**26s rRNA**	RT-primer	oligo d(T), random 9
	RT-PCR primer	Forward:taaccgcatcaggtctccaa
		Reverse:ctcgagcagttctccgacag
	Probe	ctacattgttccatcgacca
**PIRL**	RT-primer	oligo dT, random 9
	qRT-PCR primer; Construction of forward fragment of interference vector	Forward:gctctagactgatccgacctcaacaacaaccct
		Reverse: tcccccgggggacgaggtcctcgagcttctt
	PCR primer	Forward:ggaattccatatgatggatcccaaccc
		Reverse:ggggtaccaaactgctgatcaagagc
	mutated PCR	Forward:caagctcgccctgattgattcgtcgctgtcgaagaagctcgaggacctcgtcctctc
		Reverse:ggtcctcgagcttcttcgacagcgacgaatcaatcagggcgagcttggccttggcggtg
	Construction of reverse fragment of interference vector	Forward:ggggtaccggacgaggtcctcgagcttctt
		Reverse:ggaattcctgatccgacctcaacaacaaccct
